# Albumin‐Bound STING Agonist Reprograms HSPCs to Antitumor Neutrophils Enhancing CD8^+^ T Cell Immunity

**DOI:** 10.1002/advs.202523603

**Published:** 2026-03-19

**Authors:** Jinsong Tao, Hong‐Yi Zhao, Chengyi Li, Hanning Wen, Fang Ke, Qiuxia Li, Miao He, Bo Wen, Zhongwei Liu, Kai Sun, Wei Gao, Duxin Sun

**Affiliations:** ^1^ Department of Pharmaceutical Sciences College of Pharmacy Rogel Cancer Center University of Michigan Ann Arbor Michigan USA; ^2^ Department of Materials Science and Engineering College of Engineering University of Michigan Ann Arbor Michigan USA; ^3^ Department of Pharmacology and Pharmaceutical Sciences College of Pharmacy University of Houston Houston Texas USA

**Keywords:** antigen presentation, antitumor neutrophils, cancer immunotherapy, CD8^+^ T cell immunity, hematopoietic stem and progenitor cells, STING agonist

## Abstract

Tumor‐associated immunosuppressive neutrophils, termed polymorphonuclear myeloid‐derived suppressor cells (PMN‐MDSCs), compromise cancer immunotherapy. Emerging evidence indicates that neutrophil fate can be programmed as early as the hematopoietic stem and progenitor cell (HSPC) stage. Reprogramming HSPCs toward antitumor neutrophils offers a promising therapeutic strategy. Here, we demonstrate that an albumin‐bound STING agonist (Nano ZSA‐51D) reprograms HSPCs to generate antitumor neutrophils, enhancing MHC I‐mediated CD8^+^ T cell immunity and sensitizing tumors to α‐PD1 immunotherapy. Nano ZSA‐51D expands HSPCs and reprograms them toward granulocyte‐monocyte progenitors for neutrophil development. It further converts immature (CD101^−^) and mature (CD101^+^) neutrophils into a CD14^+^ICAM‐1^+^ subset through STING‐NF‐κB–TNF‐α signaling, enhancing tumor infiltration and antitumor activity. These neutrophils upregulate interferon signaling and MHC I antigen presentation, thereby boosting tumor‐specific CD8^+^ T cell responses. Notably, both adoptive transfer of Nano ZSA‐51D‐reprogrammed neutrophils and systemic Nano ZSA‐51D treatment synergizes with α‐PD1 therapy to achieve complete remission of colon tumors through neutrophil‐ and CD8^+^ T cell‐dependent mechanisms, with potent efficacy also validated in otherwise immune‐resistant pancreatic cancer models. Our findings establish a therapeutic strategy to reprogram HSPCs toward antitumor neutrophils, highlighting the potential of targeting early hematopoiesis to rewire neutrophil fate in cancer immunotherapy.

## Graphical abstract

This study demonstrates that an albumin‐bound STING agonist (Nano ZSA‐51D) reprograms HSPCs to produce antitumor neutrophils with enhanced MHC I–mediated CD8^+^ T cell activation, thereby sensitizing tumors to α‐PD1 therapy. These findings highlight a strategy to target early hematopoiesis for shaping neutrophil fate and potentiating cancer immunotherapy.

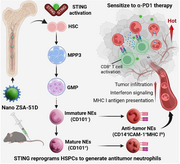

## 1 Introduction

Neutrophils (NEs), the most abundant leukocytes in human blood (50%–70%), play crucial roles in innate immunity and have emerged as key regulators of cancer immunotherapy. Tumor‐associated immunosuppressive neutrophils, often referred to as polymorphonuclear myeloid‐derived suppressor cells (PMN‐MDSCs), are frequently enriched in solid tumors and substantially compromise the efficacy of cancer immunotherapy [1, 2]. Clinical studies have shown that high neutrophil infiltration in tumors prior to treatment correlates with poor prognosis, underscoring their immunosuppressive role in the tumor microenvironment (TME) [3, 4]. These heterogeneous neutrophil populations are pathologically programmed by cancer‐derived signals to suppress cytotoxic T cell responses and promote tumor progression [5, 6].

Despite their established immunosuppressive functions, neutrophils display remarkable plasticity. Depending on the surrounding cytokine milieu and activation context, they can polarize into either antitumor (N1) or protumor (N2) subsets that exert opposing effects on tumor immunity [7, 8, 9]. Intriguingly, several immunotherapies, including immune checkpoint blockade (ICB), have been reported to induce high neutrophil infiltration associated with improved therapeutic outcomes, suggesting that neutrophils can also acquire antitumor properties under appropriate stimuli [10, 11, 12, 13, 14]. These antitumor neutrophils can directly kill tumor cells, promote antigen presentation, and enhance T cell activation, thereby suppressing tumor growth [15, 16, 17, 18, 19]. For instance, interferon‐stimulated neutrophils exhibit potent antitumor activity and enhance the efficacy of α‐PD1 therapy in both mice and humans [10, 15].

Earlier strategies primarily focused on blocking neutrophil recruitment or depleting neutrophils to alleviate immunosuppression of PMN‐MDSCs [20]. However, such approaches are clinically impractical due to neutrophils’ essential role in host defense against infections. Moreover, depletion or inhibition can transiently reduce tumor‐promoting activity but also eliminate the beneficial immune functions of neutrophils [21]. Other approaches have sought to modulate neutrophils within the TME, attempting to convert terminally differentiated neutrophils into antitumor phenotypes [22, 23, 24]. Yet, such late‐stage reprogramming is often inefficient or even irreversible due to the short lifespan and fixed differentiation state of mature neutrophils. Thus, how to effectively reprogram neutrophils into durable antitumor phenotypes to reverse their immunosuppressive functions remains a key challenge in cancer immunotherapy.

Recent evidence indicates that neutrophil functional fates are established as early as the hematopoietic stem and progenitor cell (HSPC) stage, rather than solely through interconversion within the TME [25, 26, 27, 28, 29, 30]. Tumor‐derived signals can remodel hematopoiesis by suppressing interferon signaling and imprint immunosuppressive programming onto HSPCs, leading to the development of pro‐tumor neutrophils [25, 26, 27]. This early reprogramming event provides a persistent source of immunosuppressive myeloid progeny, which continuously replenish the TME and sustain systemic immune suppression. By intervening at the progenitor level, it may be possible to rewire the developmental trajectory of neutrophils from an immunosuppressive toward an antitumor phenotype. This evidence highlights the therapeutic potential of reprogramming HSPCs toward antitumor neutrophils before exposure to cancer‐derived signals. However, whether and how HSPCs can be therapeutically reprogrammed to antitumor neutrophils for enhanced cancer immunotherapy remains largely unexplored.

**FIGURE 1:**
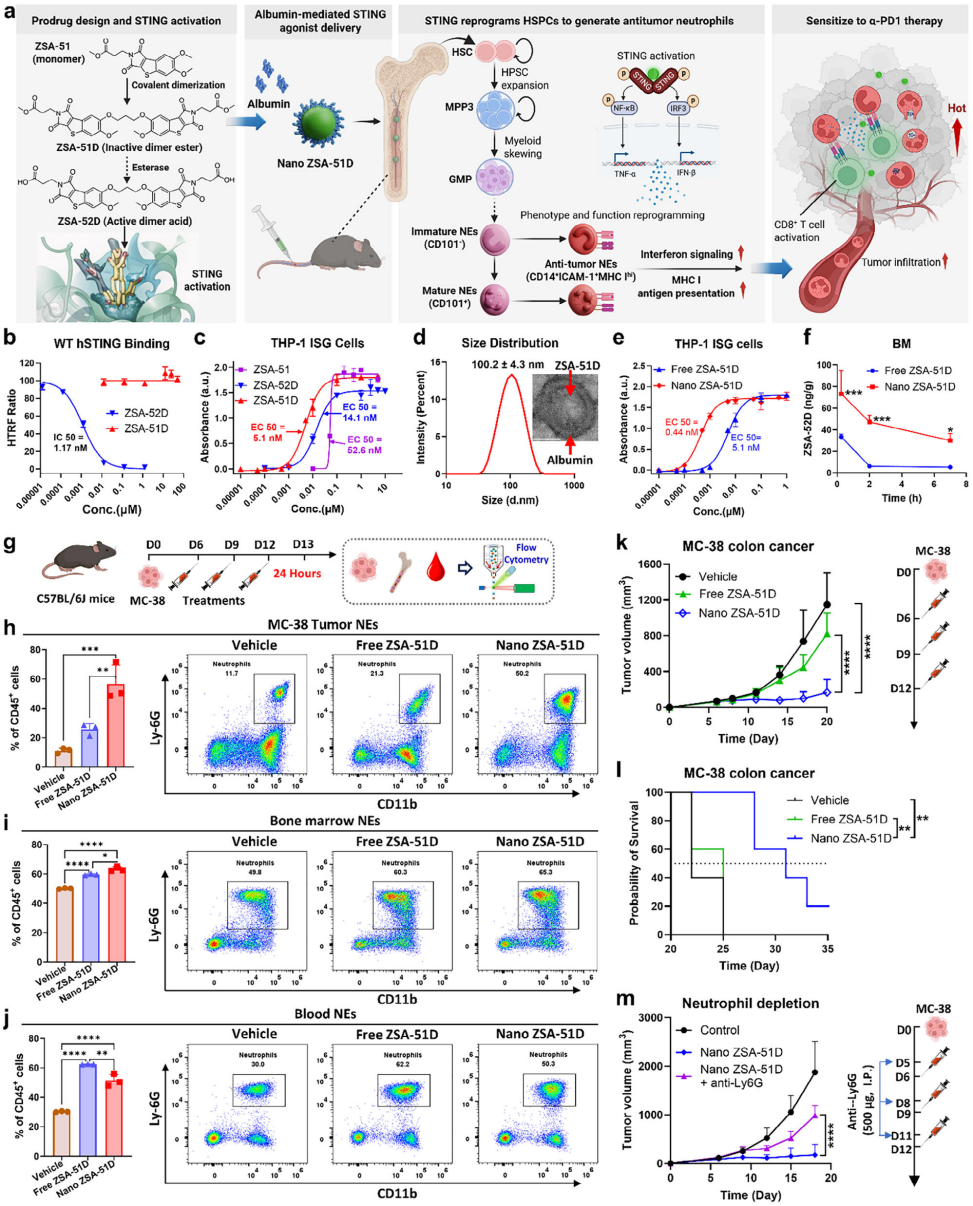
Nano ZSA‐51D potently activates STING pathway to drive neutrophil‐mediated antitumor activity. (a) Schematic illustration of Nano ZSA‐51D reprograms HSPCs into antitumor neutrophils for cancer immunotherapy. Dimeric STING agonist ZSA‐51D derived from monomeric STING agonist ZSA‐51 was encapsulated into mouse albumin to form Nano ZSA‐51D. Chemical structures of ZSA‐51, ZSA‐51D and its acid form, ZSA‐52D, along with molecular docking simulation showing ZSA‐52D (yellow) binding to the STING protein (green, PDB: 6UKZ), performed using AutoDock. Nano ZSA‐51D expands HSPCs and skews GMP differentiation, then reprograms immature (CD101^−^) and mature (CD101^+^) bone marrow neutrophils into CD14^+^ICAM‐1^+^ subsets through STING–NF‐κB–TNF‐α signaling, upregulating interferon signaling and MHC I antigen presentation to promote CD8^+^ T cell antitumor responses. Schematic created with BioRender.com. (b) Binding affinity of ZSA‐51D and ZSA‐52D to wild‐type (WT) human STING (hSTING) protein, determined by Homogeneous Time‐Resolved Fluorescence (HTRF) assay (*n* = 3, mean ± SD). (c) STING pathway activation was assessed in THP1‐Blue ISG reporter cells with virous concentrations of ZSA‐51D and ZSA‐52D for 24 h, demonstrating induction of the STING–IRF signaling cascade (*n* = 3, mean ± SD). (d) Particle size distribution and TEM image of Nano ZSA‐51D. Scare bar: 50 nm. (e) Comparison of STING‐IRF pathway activation by free and Nano ZSA‐51D in THP1‐Blue ISG reporter cells after 24‐h treatment (*n* = 3, mean ± SD). (f) Bone marrow pharmacokinetic of Free and Nano ZSA‐51D (1 mg/kg, I.V.) showing ZSA‐52D levels at 15 min, 2 h and 7 h post‐injection (*n* = 3, mean ± SD). Two‐way ANOVA with Sidak's tests: *p < 0.05, ***p < 0.001 vs. Free ZSA‐51D. (g) Schematic of treatment schedule and flow cytometry analysis to assess neutrophil populations in tumors, bone marrow and blood of MC‐38 tumor‐bearing C57BL/6J mice at 24 h post‐treatment. (h–j) Quantification (left) and representative flow cytometry plots (right) of neutrophils (NEs, CD11b^+^Ly‐6G^+^) within CD45^+^ immune cells in tumors (h), bone marrow(i) and blood (j) following free or Nano ZSA‐51D treatment (*n* = 3, mean ± SD). One‐way ANOVA with Tukey's tests: *p < 0.05, **p < 0.01, ***p < 0.001, ****p < 0.0001. (k‐l) Tumor growth curves (k) and Kaplan‐Meier survival curves (l) of MC‐38 colon cancer model following indicated treatments (right). ZSA‐51D was administered at 1 mg/kg (I.V.) (*n* = 5, mean ± SD). (k) Two‐way ANOVA with Tukey's tests: **p < 0.01, ****p < 0.001. (l) Log‐rank (Mantel–Cox) test: **p < 0.01. (m) Tumor growth curves of MC‐38 colon cancer model following the indicated treatment regimens with neutrophil depletion (right). Anti‐Ly6G (500 µg) antibody was intraperitoneally administered 1 day before each treatment with Nano ZSA‐51D (1 mg/kg, I.V.) (*n* = 5, mean ± SD). Two‐way ANOVA with Tukey's tests: ****p < 0.0001.

In this study, we demonstrate that activation of the stimulator of interferon genes (STING) pathway can reprogram HSPCs to generate antitumor neutrophils with enhanced MHC I antigen presentation, thereby promoting robust CD8^+^ T cell immunity and sensitizing tumors to α‐PD1 immunotherapy (Figure 1a). We developed an albumin‐bound STING agonist (Nano ZSA‐51D), which efficiently delivers a dimeric STING agonist to the bone marrow. Nano ZSA‐51D activates STING–interferon signaling, expands HSPCs, and reprograms toward granulocyte‐monocyte progenitors (GMPs) to drive neutrophil development. Nano ZSA‐51D further promotes immature (CD101^−^) and mature (CD101^+^) neutrophils into CD14^+^ICAM‐1^+^ subsets via STING–NF‐κB–TNF‐α signaling, thereby enhancing tumor infiltration and antitumor activity. Transcriptomic and immune profiling revealed that these neutrophils upregulated interferon signaling and MHC I antigen presentation, effectively boosting tumor‐specific CD8^+^ T cell responses. Notably, both adoptive transfer of Nano ZSA‐51D‐reprogrammed neutrophils and systemic Nano ZSA‐51D treatment synergized with α‐PD1 therapy to achieve complete remission of colon tumors through neutrophil‐ and CD8^+^ T cell‐dependent mechanisms, with potent efficacy also validated in otherwise immune‐resistant pancreatic cancer models. Together, these findings establish Nano ZSA‐51D as an effective immunotherapeutic platform that reprograms hematopoiesis to generate antitumor neutrophils, revealing the underlying mechanism to rewire neutrophil fate and potentiate cancer immunotherapy.

## 2 Results

### 2.1 Albumin‐Bound STING Agonist (Nano ZSA‐51D) Activates STING Pathway to Drive Neutrophil‐Mediated Antitumor Activity

Building on our previously developed monomeric STING agonist ZSA‐51 [31], we synthesized a novel non‐CDN dimeric STING agonist, ZSA‐51D, which exhibited superior STING activation (Figure 1a, Left and Figure S1). ZSA‐51D is an ester‐based prodrug that requires intracellular hydrolysis by esterases to convert into its active acid form, ZSA‐52D, before binding to STING protein. Due to enhanced cell permeability, ZSA‐51D readily enters cells and is converted to ZSA‐52D, resulting in potent and sustained STING activation. As anticipated, ZSA‐51D itself did not bind human STING, whereas ZSA‐52D showed potent binding (IC_50_ = 1.17 nm) (Figure 1b). In THP1‐Blue ISG reporter cells, ZSA‐51D potently activated the STING pathway (EC_50_ = 5.1 nm), outperforming ZSA‐52D (EC_50_ = 14.1 nm) and monomeric ZSA‐51 (EC_50_ = 52.6 nm) (Figure 1c).

To enable systemic delivery, ZSA‐51D was encapsulated into an albumin nanoparticle (Nano ZSA‐51D) [32, 33, 34]. The average particle size of Nano ZSA‐51D was 100.2 ± 4.3 nm (Figure 1d). Transmission electron microscopy (TEM) confirmed the formation of uniform, spherical nanoparticles in which ZSA‐51D was encapsulated within an albumin matrix (Figure 1d; Figure S2). Nano ZSA‐51D enhanced STING activation by 11.6‐fold (EC_50_ = 0.44 nm) compared to free ZSA‐51D (EC_50_ = 5.1 nm) in THP1‐Blue ISG cells (Figure 1e). Pharmacokinetic analysis showed that Nano ZSA‐51D enhanced bone marrow delivery compared to free ZSA‐51D (Figure 1f), while tumor and blood distribution remained comparable (Figure S3). Western blotting confirmed that Nano ZSA‐51D significantly enhanced phosphorylation of STING, IRF3 and NF‐κB p65 in bone marrow cells, thereby promoting the production of IFN‐β, TNF‐α, and IL‐6 (Figure S4) [35], indicating activation of both the STING‐IRF3 and STING‐NF‐κB signaling axes.

To further examine neutrophil responses following systemic administration of Nano ZSA‐51D (1 mg/kg, I.V.), we analyzed the neutrophil populations in tumors, bone marrow, and peripheral blood of MC‐38 tumor‐bearing mice 24 h after treatment (Figure 1g). Nano ZSA‐51D dramatically increased neutrophils within tumors from 11.2% to 56.7% of CD45^+^ immune cells, a substantially greater increasement than free ZSA‐51D (Figure 1h). In parallel, both free and Nano ZSA‐51D elevated neutrophil frequencies in bone marrow and peripheral blood (Figure 1i,j). Together, these data demonstrate that Nano ZSA‐51D drives neutrophil expansion and preferential recruitment into tumors.

To determine whether Nano ZSA‐51D‐induced neutrophil infiltration contributes to antitumor activity, we evaluated the therapeutic efficacy in the MC‐38 colon cancer model. Nano ZSA‐51D markedly suppressed tumor growth and extended survival compared with free ZSA‐51D, correlating with a significant increase in tumor‐infiltrating neutrophils (Figure 1k,l). Notably, depletion of neutrophils using anti‐Ly6G antibody dramatically abolished the therapeutic benefit of Nano ZSA‐51D (Figure 1m), demonstrating that these neutrophils possess antitumor activity and are critical mediators of Nano ZSA‐51D–induced tumor control.

### 2.2 Nano ZSA‐51D Expands HSPCs and Reprograms toward GMPs to Promote Neutrophil Development

**FIGURE 2:**
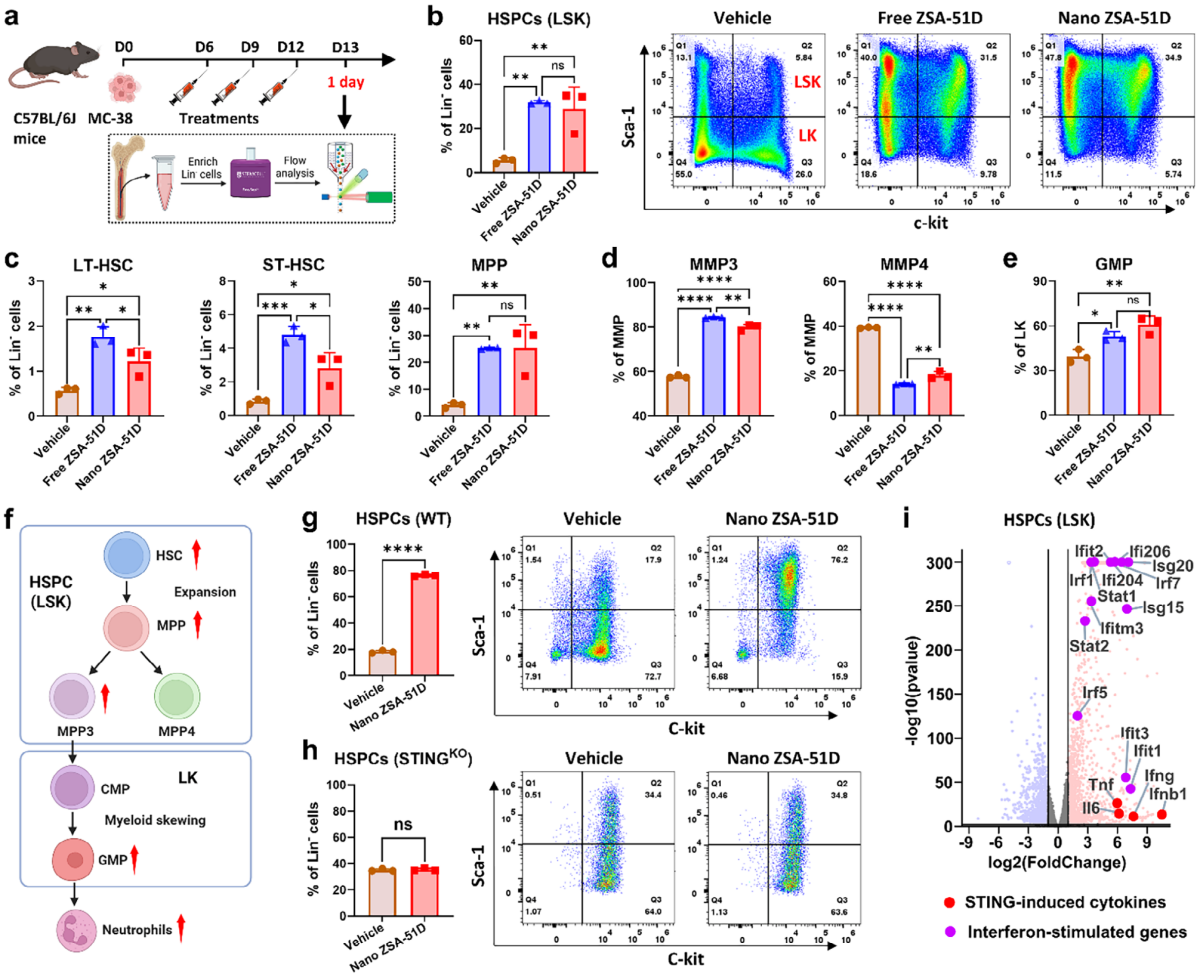
Nano ZSA‐51D expands HSPCs and reprograms toward GMPs to promote neutrophil development. (a) Schematic illustration of treatment schedule and flow cytometry analysis to examine the composition of HSPC subsets in bone marrow of MC‐38 tumor bearing mice 24 h post‐treatment. The flow cytometry analysis of HSPCs was performed after enrichment of Lin^−^ cells. (b) Quantification (left) and representative flow cytometry plots (right) of HSPCs (Lin^−^Sca‐1^+^c‐Kit^+^, LSK) within Lin^−^ cells in bone marrow following free or Nano ZSA‐51D treatment (*n* = 3, mean ± SD). One‐way ANOVA with Tukey's tests: **p < 0.01; ns, not significant. (c) Quantification (left) of LT‐HSC (CD150^+^CD48^−^), ST‐HSC (CD150^−^CD48^−^) and MPP (CD150^−/+^CD48^+^) within Lin^−^ cells in bone marrow following free or Nano ZSA‐51D treatment (*n* = 3, mean ± SD). One‐way ANOVA with Tukey's tests: *p < 0.05, **p < 0.01, ***p < 0.001; ns, not significant. (d) Quantification of myeloid‐biased multipotent progenitor 3 (MPP3, CD34^+^CD135^−^) and lymphoid‐biased multipotent progenitor 4 (MPP4, CD34^+^CD135^+^) within MMP (Lin^−^Sca‐1^+^c‐Kit^+^CD150^−/+^CD48^+^) in bone marrow following free or Nano ZSA‐51D treatment (*n* = 3, mean ± SD). One‐way ANOVA with Tukey's tests: **p < 0.01, ****p < 0.0001. (e) Quantification of granulocyte‐monocyte progenitor (GMP, CD150^−^CD16/32^+^) within LK (Lin^−^Sca‐1^−^c‐Kit^+^) in bone marrow following free or Nano ZSA‐51D treatment (*n* = 3, mean ± SD). One‐way ANOVA with Tukey's tests: *p < 0.05, **p < 0.01; ns, not significant. (f) Schematic illustration of HSPCs expansion and reprogramming toward GMP after STING agonist treatments. HSC, hematopoietic stem cell; MPP, multipotent progenitor; MPP3, myeloid‐biased multipotent progenitor 3; MPP4, lymphoid‐biased multipotent progenitor 4; CMP, common myeloid progenitor; GMP, granulocyte‐monocyte progenitor. Schematic created with BioRender.com. (g‐h) Quantification (left) and representative flow cytometry plots (right) of HSPCs (LSK) within Lin^−^ cells from WT or STING‐deficient (C57BL/6J‐Sting1^gt/J) mice after 3 days of in vitro treatment with Nano ZSA‐51D (*n* = 3, mean ± SD). Unpaired two‐tailed Student's t‐tests: ****p < 0.0001, ns, not significant. (i) Volcano plot of differentially expressed gene analysis between vehicle and Nano ZSA‐51D treated HSPCs. Significance threshold for P adjusted value was 0.01 and foldchange was 2. The STING‐induced cytokines (red dots) and interferon‐stimulated genes (purple dots) were highlighted.

Neutrophils arise from HSPCs in the bone marrow through a series of lineage‐restricted differentiation stages (Figure S5A). To investigate how Nano ZSA‐51D drives neutrophil development in vivo, we analyzed the composition of HSPC subsets in bone marrow following treatment (Figure 2a).

Interestingly, both free and Nano ZSA‐51D markedly expanded the HSPC compartment (Lin^−^Sca‐1^+^c‐Kit^+^, LSK) (Figure 2b), which includes long‐term hematopoietic stem cells (LT‐HSCs), short‐term HSC (ST‐HSCs), and multipotent progenitor (MPPs) (Figure 2c; Figure S5B) [36]. Notably, MPPs exhibited the most dramatic expansion, rising from 4.1% to 25.3% of Lin^−^ cells. Among the MPPs, the myeloid‐biased MPP3 subset increased highly, while the lymphoid‐biased MPP4 decreased (Figure 2d; Figure S5C). In downstream progenitors, we also observed an increase in granulocyte‐monocyte progenitors (GMPs) (Figure 2e; Figure S5D). These results suggest that Nano ZSA‐51D expands HSPCs and reprograms them toward GMPs, thereby potentially enhancing myelopoiesis to drive neutrophil production in bone marrow (Figure 2f).

To determine whether STING agonist directly drives HSPC expansion and reprograms toward GMPs, we isolated the HSPCs (LSK) from the bone marrow of MC‐38 tumor‐bearing C57BL/6J mice and treated them with Nano ZSA‐51D (100 nm) in vitro for 3 days. Interestingly, Nano ZSA‐51D dramatically expanded HSPCs (LSK) from 18.1% to 76.3% (Figure 2g), whereas vehicle‐treated groups exhibited the differentiation of HSPCs from LSK to LK populations. This observation suggests that STING activation can directly expand HSPCs at stem and multipotent progenitor states. Notably, Nano ZSA‐51D failed to expand HSPCs (LSK) from STING‐deficient (C57BL/6J‐Sting1^gt/J) mice (Figure 2h), confirming that this effect is STING‐dependent.

Tumor‐derived signals are known to suppress interferon signaling in bone marrow progenitors, reprogramming their differentiation toward immunosuppressive MDSCs [30]. To determine whether Nano ZSA‐51D activates STING‐interferon pathway signaling, we performed mRNA sequencing on vehicle‐ and Nano ZSA‐51D‐treated HSPCs. Nano ZSA‐51D significantly upregulated STING‐induced cytokines (IFN‐β, TNF‐α, IFN‐γ, IL‐6) and a broad panel of interferon‐stimulated genes (ISGs: Irf1, Irf5, Irf7, Stat1, Stat2, Ifit1, and Isg15, etc.) (Figure 2i), demonstrating robust activation of STING–interferon signaling in HSPCs. Collectively, these results indicate that Nano ZSA‐51D reprograms the transcriptional landscape of HSPCs toward an antitumor immunostimulatory phenotype.

### 2.3 Nano ZSA‐51D Reprograms Neutrophils into CD14^+^ICAM‐1^+^ Subset via STING–NF‐κB–TNF‐α Signaling to Enhance Tumor Infiltration

Recent studies have shown that tumor‐infiltrating neutrophils with high expression of interferon‐stimulated genes (ISGs) and an immature/activated (CD101^−^CD14^+^) phenotype are associated with improved responses to immunotherapy [10, 15, 37, 38]. CD101 serves as a neutrophil maturation marker and whereas CD14 is the activation marker of pattern recognition receptor‐mediated immune responses [39, 40, 41, 42]. To investigate how Nano ZSA‐51D reprograms neutrophils to enhance tumor infiltration and antitumor activity, we analyzed neutrophil phenotypes in the bone marrow, peripheral blood, and tumors of MC‐38 tumor‐bearing mice 24 h after treatment (Figure S6A).

**FIGURE 3:**
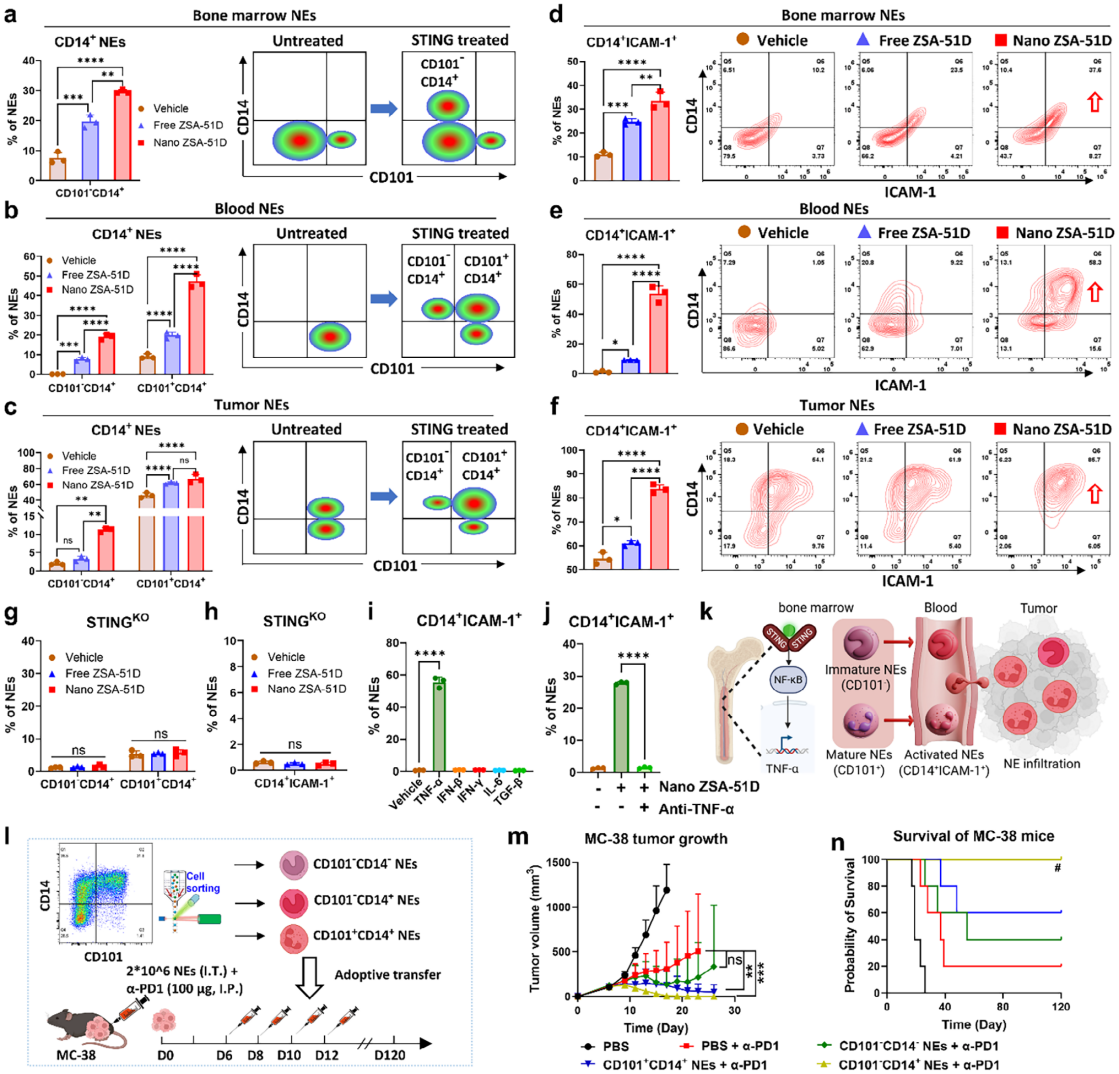
Nano ZSA‐51D reprograms neutrophils into CD14^+^ICAM‐1^+^ subset via STING–NF‐κB–TNF‐α signaling, driving complete colon tumor remission with α‐PD1 therapy. (a–c) Quantification (left) and schematic illustration (right) of CD101^−^CD14^+^ and CD101^+^CD14^+^ neutrophil (NE) subtypes in bone marrow (a), blood (b) and tumors (c) of MC‐38 tumor‐bearing C57BL/6J mice 24 h after systemic administration of free and Nano ZSA‐51D (1 mg/kg) (*n* = 3, mean ± SD). One‐way (a) or two‐way (b,c) ANOVA with Tukey's tests: **p < 0.01, ***p < 0.001, ****p < 0.0001; ns, not significant. (d–f) Quantification (left) and representative flow cytometry plots (right) of CD14^+^ICAM‐1^+^ neutrophil subtypes in bone marrow (d), blood (e), and tumors (f) after systemic administration of free and Nano ZSA‐51D (1 mg/kg) (*n* = 3, mean ± SD). One‐way ANOVA with Tukey's tests: *p < 0.05, **p < 0.01, ***p < 0.001, ****p < 0.0001. (g,h) Quantification of CD101^−^CD14^+^, CD101^+^CD14^+^ (g) and CD14^+^ICAM‐1^+^ (h) neutrophils after overnight in vitro treatments with free and Nano ZSA‐51D (100 nm) in bone marrow cells from STING knockout mice (C57BL/6J‐Sting1gt/J) (*n* = 3, mean ± SD). One‐way (h) or two‐way (g) ANOVA with Tukey's tests: ns, not significant. (i) Quantification of CD14^+^ICAM‐1^+^ neutrophils following in vitro treatments of the STING pathway downstream cytokines (TNF‐α, IFN‐β, IFN‐γ, and IL‐6) for overnight. The immunosuppressive TGF‐β as control (*n* = 3, mean ± SD). One‐way ANOVA with Tukey's tests: ****p < 0.0001. (j) Quantification and changes of CD14^+^ICAM‐1^+^ neutrophils after overnight in vitro treatment with Nano ZSA‐51D (100 nm) and TNF‐α neutralization (10 µg/mL anti‐TNF‐α) (*n* = 3, mean ± SD). One‐way ANOVA with Tukey's tests: ****p < 0.0001. (k) Schematic illustration of STING activation‐induced reprogramming of immature (CD101^−^) and mature (CD101^+^) bone marrow neutrophils into activated CD14^+^ICAM‐1^+^ subsets with enhanced tumor infiltration. Schematic created with BioRender.com. (l) Schematic experiment of neutrophil adoptive transfer. CD101^−^CD14^−^, CD101^−^CD14^+^ and CD101^+^CD14^+^ neutrophils (NEs) were sorted from bone marrow cells pretreated overnight with Nano ZSA‐51D (100 nm), then intratumorally injected into MC‐38 tumor‐bearing mice alongside intraperitoneal α‐PD1 (100 µg/mouse). (m, n) Tumor growth curves (m) and Kaplan‐Meier survival curves (n) of MC‐38 tumor‐bearing mice after adoptive transfer of CD101^−^CD14^−^, CD101^−^CD14^+^ or CD101^+^CD14^+^ neutrophils in combination with α‐PD1 (100 µg/mice) treatments (*n* = 5, mean ± SD). (m) Two‐way ANOVA with Tukey's tests: **p < 0.01, ***p < 0.001; ns, not significant. (n) Log‐rank (Mantel–Cox) tests: #*p* < 0.05 vs PBS + α‐PD1.

In the bone marrow of vehicle‐treated mice, most neutrophils were immature/inactivated (CD101^−^CD14^−^). Nano ZSA‐51D increased immature/activated (CD101^−^CD14^+^) neutrophils from 7.5% to 29.6%, which was higher than free ZSA‐51D (Figure 3a; Figure S6B). In the blood, Nano ZSA‐51D dramatically expanded both immature/activated (CD101^−^CD14^+^) neutrophils from 0.08% to 20%, and mature/activated (CD101^+^CD14^+^) neutrophils from 9.1% to 47.2%, which were both substantially higher than free ZSA‐51D (Figure 3b; Figure S6C). It suggests that Nano ZSA‐51D robustly activates both immature and mature neutrophils into CD14^+^ phenotype.

Within tumors, activated CD14^+^ neutrophils were preferentially recruited following Nano ZSA‐51D treatment, resulting in enhanced neutrophil infiltration and antitumor activity. Specifically, the proportion of immature/activated (CD101^−^CD14^+^) neutrophils increased from 2.0% to 11.2%, and mature/activated (CD101^+^CD14^+^) neutrophils from 46.1% to 66.8% (Figure 3c; Figure S6D). In contrast, free ZSA‐51D induced fewer CD14^+^ neutrophils in bone marrow and blood, resulting in limited tumor infiltration (Figure 3a–c). In addition, Nano ZSA‐51D induced strong ICAM‐1 (CD54) expression on CD14^+^ neutrophils in bone marrow and blood circulation, promoting CD14^+^ICAM‐1^+^ neutrophil infiltration into tumor (Figure 3d–f) [43, 44]. These data suggest that Nano ZSA‐51D reprograms both immature (CD101^−^) and mature (CD101^+^) neutrophils into activated CD14^+^ICAM‐1^+^ subsets that enhance infiltration into tumors.

To confirm the role of STING activation in reprogramming CD14^+^ICAM‐1^+^ neutrophils, bone marrow cells from wild‐type (WT) and STING‐ knockout (C57BL/6J‐Sting1gt/J) mice were treated with free or Nano ZSA‐51D (100 nm) in vitro. Nano ZSA‐51D significantly increased CD101^−^CD14^+^, CD101^+^CD14^+^, and CD14^+^ICAM‐1^+^ populations in WT mice, but not in STING‐deficient mice (Figure 3g,h; Figure S7), confirming that neutrophil reprogramming requires STING signaling.

To further identify the downstream mediators of STING signaling, we treated the isolated bone marrow neutrophils with TNF‐α, IFN‐β, IFN‐γ, and IL‐6. TGF‐β was used as an immunosuppressive signaling. Among these, only TNF‐α induced the CD101^−^CD14^+^, CD101^+^CD14^+^ and CD14^+^ICAM‐1^+^ subsets (Figure 3i; Figure S8A–D). Neutralization of TNF‐α completely abolished Nano ZSA‐51D–induced CD14^+^ICAM‐1^+^ reprogramming (Figure 3j; Figure S8E). These findings suggest that Nano ZSA‐51D reprograms immature and mature neutrophils into CD14^+^ICAM‐1^+^ subsets via STING‐NF‐κB‐TNF‐α signaling, thereby enhancing their tumor infiltration (Figure 3k).

### 2.4 Nano ZSA‐51D‐Reprogrammed Neutrophils Synergize With α‐PD1 Therapy to Drive Complete Colon Tumor Remission

To determine whether Nano ZSA‐51D‐reprogrammed bone marrow neutrophils possess antitumor activity, we isolated three neutrophil subsets from bone marrow cells treated with Nano ZSA‐51D (100 nm) in vitro: immature/inactivated (CD101^−^CD14^−^), immature/activated (CD101^−^CD14^+^) and mature/activated (CD101^+^CD14^+^). Each subset was adoptively transferred intratumorally (I.T.) into MC‐38 tumors in combination with α‐PD1 therapy (Figure 3l).

Remarkably, the combination of CD101^−^CD14^+^ neutrophil transfer and α‐PD1 achieved 100% complete tumor remission (100% CR) without recurrence, resulting in 100% long‐term survival (Figure 3m,n; Figure S9). The CD101^+^CD14^+^ subset also significantly enhanced therapeutic efficacy, achieving a 60% CR rate, whereas CD101^−^CD14^−^ neutrophils showed no appreciable improvement compared with α‐PD1 monotherapy. These findings demonstrate that Nano ZSA‐51D‐reprogrammed CD14^+^ neutrophils possess potent antitumor properties that synergize with α‐PD1 therapy.

To investigate whether CD101^−^CD14^+^ neutrophils establish durable immune protection, mice that achieved complete remission were rechallenged with MC‐38 tumor cells 120 days post‐treatment (Figure S10A). All rechallenged mice completely resisted tumor regrowth (Figure S10B), indicating the establishment of long‐term antitumor immune memory.

Further analysis of memory T cells at 180 days post‐rechallenge revealed a significant increase in CD8^+^ T central memory (TCM) cells, with a 1.53‐, 2.40‐, and 1.84‐fold increase in spleen, lymph nodes, and blood compared with naïve controls (Figure S10C,E,F). CD4^+^ T effector memory (TEM) cells also increased across all tissues, whereas CD4^+^ TCM cells remained unchanged (Figure S10D,F,H). These results demonstrate that adoptive transfer of Nano ZSA‐51D–reprogrammed CD101^−^CD14^+^ neutrophils with α‐PD1 therapy establishes durable CD8^+^ T cell memory, driving complete tumor remission and long‐lasting tumor immunity.

### 2.5 Nano ZSA‐51D Reprograms Neutrophils to Enhance MHC I–Mediated Antigen Presentation

**FIGURE 4:**
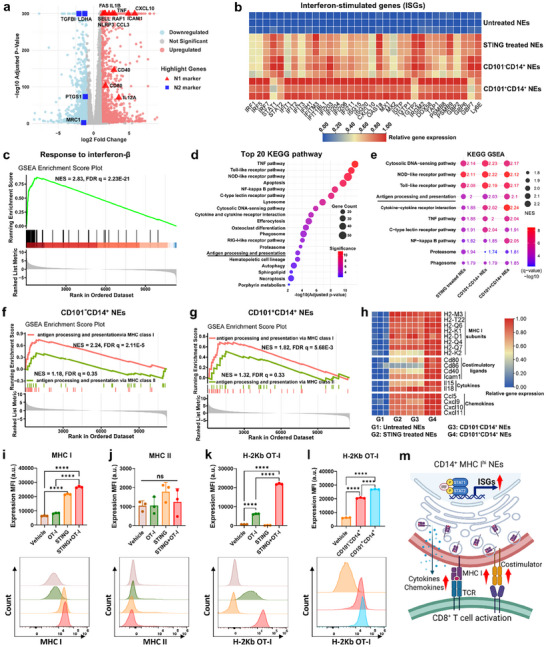
Nano ZSA‐51D reprograms neutrophils to enhance MHC I–Mediated antigen presentation. (a) Volcano plot of differentially expressed gene analysis between vehicle and Nano ZSA‐51D treated neutrophils. Significance threshold for P adjusted value was 0.01 and foldchange was 2. The antitumor (N1, red triangle) and protumor (N2, blue square) neutrophil markers were highlighted. (b) Heatmap of relative interferon‐stimulated gene (ISG) expression in Nano ZSA‐51D‐treated neutrophils (NEs) and their CD101^−^CD14^+^ and CD101^+^CD14^+^ subtypes, compared to untreated neutrophils. (c) Gene Ontology (GO) GESA enrichment score plots for biological process that response to interferon‐beta (IFN‐β) in Nano ZSA‐51D treated neutrophils. (d) Top 20 KEGG pathway enrichment analysis based on the differentially expressed genes (DEGs) in neutrophils from Nano ZSA‐51D‐treated and vehicle‐treated groups, ranked by adjusted p‐values. (e) Top 10 upregulated pathways in Nano ZSA‐51D‐treated neutrophils and their CD101^−^CD14^+^ and CD101^+^CD14^+^ subtypes, identified by KEGG Gene Set Enrichment Analysis (GESA). Normalized enrichment score (NES > 1.5) indicates significant upregulation. FDR q‐value (q < 0.05) denotes statistical significance after false discovery rate adjustment. (f, g) Gene Ontology (GO) GESA enrichment score plots for antigen processing and presentation via MHC class I and II in CD101^−^CD14^+^ (f) and CD101^+^CD14^+^ (g) neutrophils. Normalized enrichment score (NES > 1.5) indicates significant upregulation. FDR q‐value (q < 0.05) denotes statistical significance after false discovery rate adjustment. (h) Heatmap of relative gene expression of MHC class I subunits, costimulatory ligands, T cell‐activating cytokines and T cell‐recruiting chemokines in Nano ZSA‐51D‐treated neutrophils and their CD101^−^CD14^+^ and CD101^+^CD14^+^ subtypes. (i, j) Protein expression of MHC class I (i) and II (j) molecules on bone marrow neutrophils following treatments with OT‐I or/and Nano ZSA‐51D (*n* = 3, mean ± SD). One‐way ANOVA with Tukey's tests: ****p < 0.0001; ns, not significant. (k) Presentation of OT‐I peptide via MHC class I (H‐2K^b^‐OT‐I complex) on neutrophils following treatments with OT‐I or/and Nano ZSA‐51D (*n* = 3, mean ± SD). One‐way ANOVA with Tukey's tests: ****p < 0.0001. (l) Presentation of OT‐I peptide via MHC class I (H‐2K^b^‐OT‐I complex) on CD101^−^CD14^+^ and CD101^+^CD14^+^ neutrophils following OT‐I treatment (*n* = 3, mean ± SD). One‐way ANOVA with Tukey's tests: ****p < 0.0001. (m) Schematic illustration of antigen presentation by ISG‐expressing CD14^+^MHC I^hi^ neutrophils via MHC class I to activate CD8^+^ T cells. Schematic created with BioRender.com.

To elucidate how Nano ZSA‐51D‐reprogrmmed neutrophils contribute to antitumor immunity, we performed bulk mRNA sequencing on sorted total neutrophils and their subtypes from bone marrow cells following Nano ZSA‐51D (100 nm) treatment in vitro. Differential gene expression analysis revealed upregulation of antitumor (N1) markers and downregulation of protumor (N2) markers, indicating a shift toward an N1‐like neutrophil phenotype [22, 45, 46] (Figure 4a).

Downregulation of interferon signaling is a key driver of immunosuppressive phenotype in PMN‐MDSCs [32, 33]. In contrast, Nano ZSA‐51D treatment markedly upregulated interferon‐stimulated genes (ISGs) in neutrophils (Figure 4b). Gene Ontology (GO) Gene set enrichment analysis (GSEA) revealed strong enrichment of the response to interferon‐β pathway (NES = 2.83, FDR q = 2.23E‐21) (Figure 4c), demonstrating that STING activation by Nano ZSA‐51D drives IFN‐β–mediated interferon signaling in neutrophils.

KEGG analysis further revealed that the innate immune signaling pathways were highly enriched and upregulated, including Cytosolic DNA‐sensing, NOD‐like receptor, toll‐like receptor and C‐type lectin receptor pathways, along with their downstream TNF and NF‐κB cascades (Figure 4d,e; Figure S11A,B). These innate pathways endow neutrophils with enhanced capacity to sense pathogen‐associated molecular patterns (PAMPs) and damage‐associated molecular patterns (DAMPs) within TME [47, 48, 49, 50].

A particularly striking finding was the significant upregulation of antigen processing and presentation pathway in Nano ZSA‐51D‐reprogrammed immature/activated (CD101^−^CD14^+^) (NES = 2.03, FDR q = 3.49E‐5) and mature/activated (CD101^+^CD14^+^) (NES = 2.1, FDR q = 2.24E‐6) neutrophils (Figure 4e; Figure S11C). GO GSEA analysis further confirmed strong upregulation of MHC class I‐mediated antigen presentation (Figure 4f,g).

Consistently, Nano ZSA‐51D markedly increased expression of MHC I subunits, costimulatory molecules, cytokines and chemokines in neutrophils that promote T cell activation and recruitment, further reinforcing their antigen‐presenting potential (Figure 4h). In contrast, MHC II subunit expression remained unchanged (Figure S11D).

Flow cytometry analysis further confirmed that Nano ZSA‐51D significantly elevated surface MHC I expression on neutrophils (Figure 4i), but not MHC II (Figure 4j), and enhanced MHC I presentation of OT‐I peptide (SSIINFEKL) by 3.6‐fold (Figure 4k). Both immature/activated (CD101^−^CD14^+^) and mature/activated (CD101^+^CD14^+^) neutrophils displayed superior MHC I antigen presenting capacity compared to vehicle‐treated controls (Figure 4l). These results suggest that Nano ZSA‐51D reprograms neutrophils to enhance MHC I‐mediated antigen‐presentation, thereby equipping them to effectively prime CD8^+^ T cell immunity (Figure 4m).

### 2.6 Nano ZSA‐51D‐Reprogrammed Neutrophils Prime Antigen‐Specific CD8^+^ T Cell Responses

**FIGURE 5:**
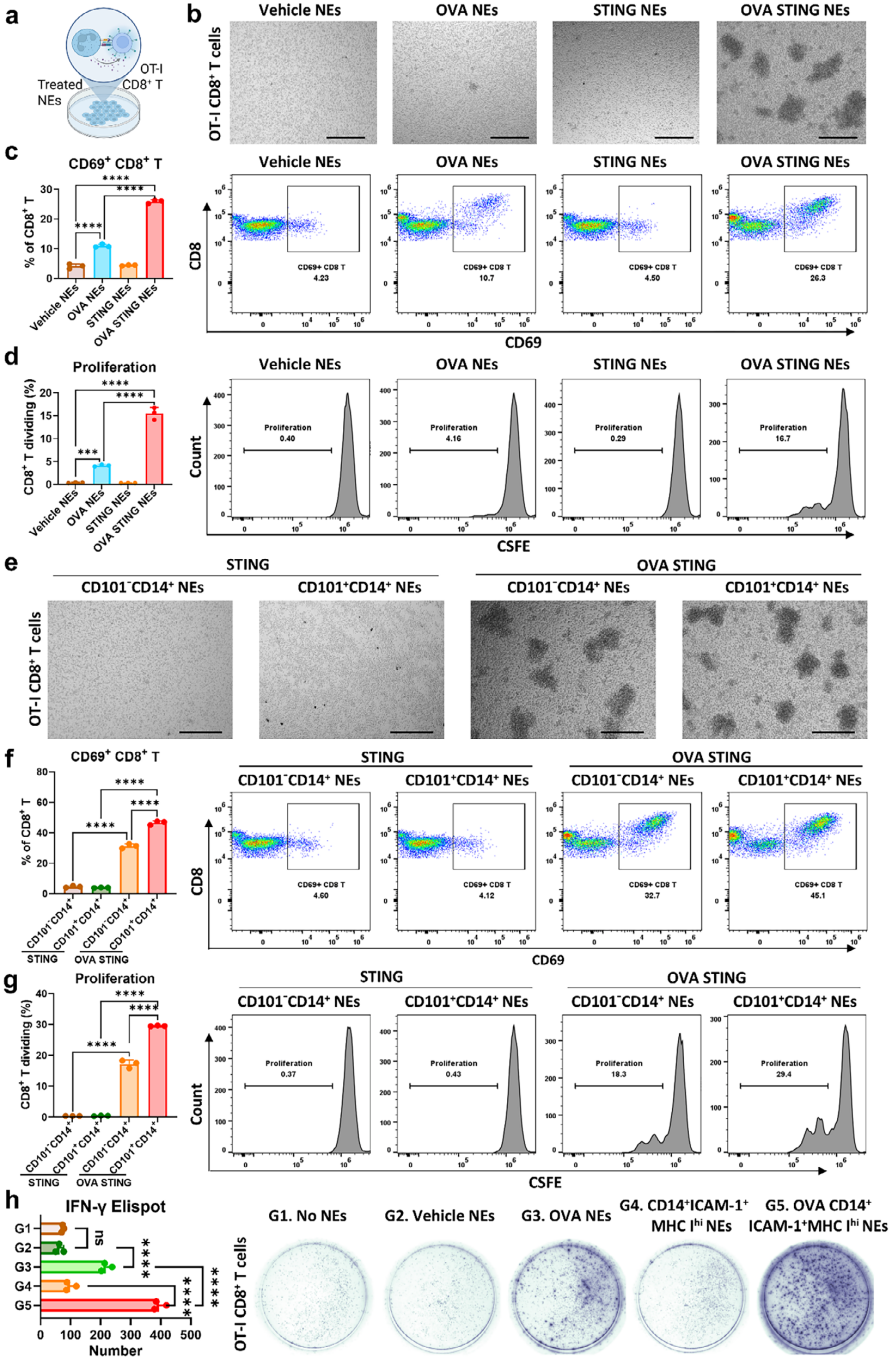
Nano ZSA‐51D‐reprogrammed neutrophils prime antigen‐specific CD8^+^ T cell responses. (a) Experimental schematic showing the co‐incubation of neutrophils (NEs) with OT‐I CD8^+^ T cells. Neutrophils were sorted from bone marrow pretreated overnight with OVA (0.2 mg/mL) and/or STING (Nano ZSA‐51D, 100 nm). (b) Bright‐field microscopy images of OT‐I CD8^+^ T cells after co‐incubation with differently treated bone marrow neutrophils (NEs) for 48 h. Scale bars: 300 µm. (c) Quantification (left) and representative flow cytometry plots (right) of CD69^+^ OT‐I CD8^+^ T cells after co‐incubation with differently treated neutrophils for 48 h (*n* = 3, mean ± SD). One‐way ANOVA with Tukey's tests: ****p < 0.0001. (d) Quantification (left) and representative flow cytometry plots (right) of OT‐I CD8^+^ T cell proliferation by CSFE staining after co‐incubation with differently treated neutrophils for 48 h (*n* = 3, mean ± SD). One‐way ANOVA with Tukey's tests: ***p < 0.001, ****p < 0.0001. (e) Bright‐field microscopy images of OT‐I CD8^+^ T cells after co‐incubation with differently treated CD101^−^CD14^+^ and CD101^+^CD14^+^ neutrophils for 48 h. CD101^−^CD14^+^ and CD101^+^CD14^+^ neutrophils were sorted from bone marrow pretreated overnight with STING (Nano ZSA‐51D) and OVA. Scale bars: 300 µm. (f) Quantification (left) and representative flow cytometry plots (right) of CD69^+^ OT‐I CD8^+^ T cell after co‐incubation with OVA treated CD101^−^CD14^+^ and CD101^+^CD14^+^ neutrophils for 48 h (*n* = 3, mean ± SD). One‐way ANOVA with Tukey's tests: ****p < 0.0001. (g) Quantification (left) and representative flow cytometry plots (right) of OT‐I CD8^+^ T cell proliferation by CSFE staining after co‐incubation with OVA treated CD101^−^CD14^+^ and CD101^+^CD14^+^ neutrophils for 48 h (*n* = 3, mean ± SD). One‐way ANOVA with Tukey's tests: ****p < 0.0001. (h) ELISPOT quantification (left) and representative images (right) of OT‐I CD8^+^ T cells following 24 h co‐incubation with neutrophils subjected to the indicated treatments (*n* = 3, mean ± SD). One‐way ANOVA with Tukey's tests: ****p < 0.0001.

To determine whether Nano ZSA‐51D‐reprogrammed neutrophils can prime antigen‐specific CD8^+^ T cell responses through MHC I‐mediated antigen presentation, we performed co‐culture assays using OT‐I CD8^+^ T cells from transgenic OT‐I mice. Neutrophils were sorted from bone marrow cells that pre‐treated with ovalbumin (OVA, 0.2 mg/mL) and Nano ZSA‐51D (100 nm) and then co‐incubated with OT‐I CD8^+^ T cells for 48 h (Figure 5a).

Strikingly, OVA and Nano ZSA‐51D‐treated neutrophils induced extensive OT‐I CD8^+^ T cell clustering (Figure 5b), indicating robust recruitment and activation. Flow cytometry further confirmed potent CD8^+^ T cell activation, as indicated by the significant increase of CD69, CD25 and CD137 (4‐1BB) expression (Figure 5c; Figure S12A,B), and markedly enhanced CD8^+^ T cell proliferation (Figure 5d). These data suggest Nano ZSA‐51D‐reprogrammed neutrophils effectively activate CD8^+^ T cells via enhanced MHC I antigen presentation.

We next assessed the antigen‐presenting capacity of distinct activated neutrophil subsets. Immature/activated (CD101^−^CD14^+^) and mature/activated (CD101^+^CD14^+^) neutrophils were sorted from OVA and Nano ZSA‐51D‐treated bone marrow and co‐incubated with OT‐I CD8^+^ T cells for 48 h. Both subsets induced pronounced CD8^+^ T cell clustering (Figure 5e), upregulated CD69, CD25, and CD137 (4‐1BB) (Figure 5f; Figure S12C,D) and significantly promoted CD8^+^ T cell proliferation (Figure 5g). Notably, the CD14^+^ICAM‐1^+^MHC I^hi^ neutrophils elicited antigen‐dependent IFN‐γ production by OT‐I CD8^+^ T cells (Figure 5h). We also examined the capacity of STING‐activated neutrophils to stimulate OT‐II CD4^+^ T cells in the presence of OVA antigen. Consistent with their low MHC II expression, CD4^+^ T cell activation remained minimal (Figure S13). Collectively, these results demonstrate that Nano ZSA‐51D‐reprogrammed immature and mature CD14^+^ neutrophils possess potent antigen‐presenting activity capable of priming antigen‐specific CD8^+^ T‐cell activation and proliferation.

### 2.7 Nano ZSA‐51D Synergizes with α‐PD1 to Achieve Potent Efficacy in Colon and Pancreatic Cancers through Neutrophil‐ and CD8^+^ T Cell‐Dependent Mechanisms

**FIGURE 6:**
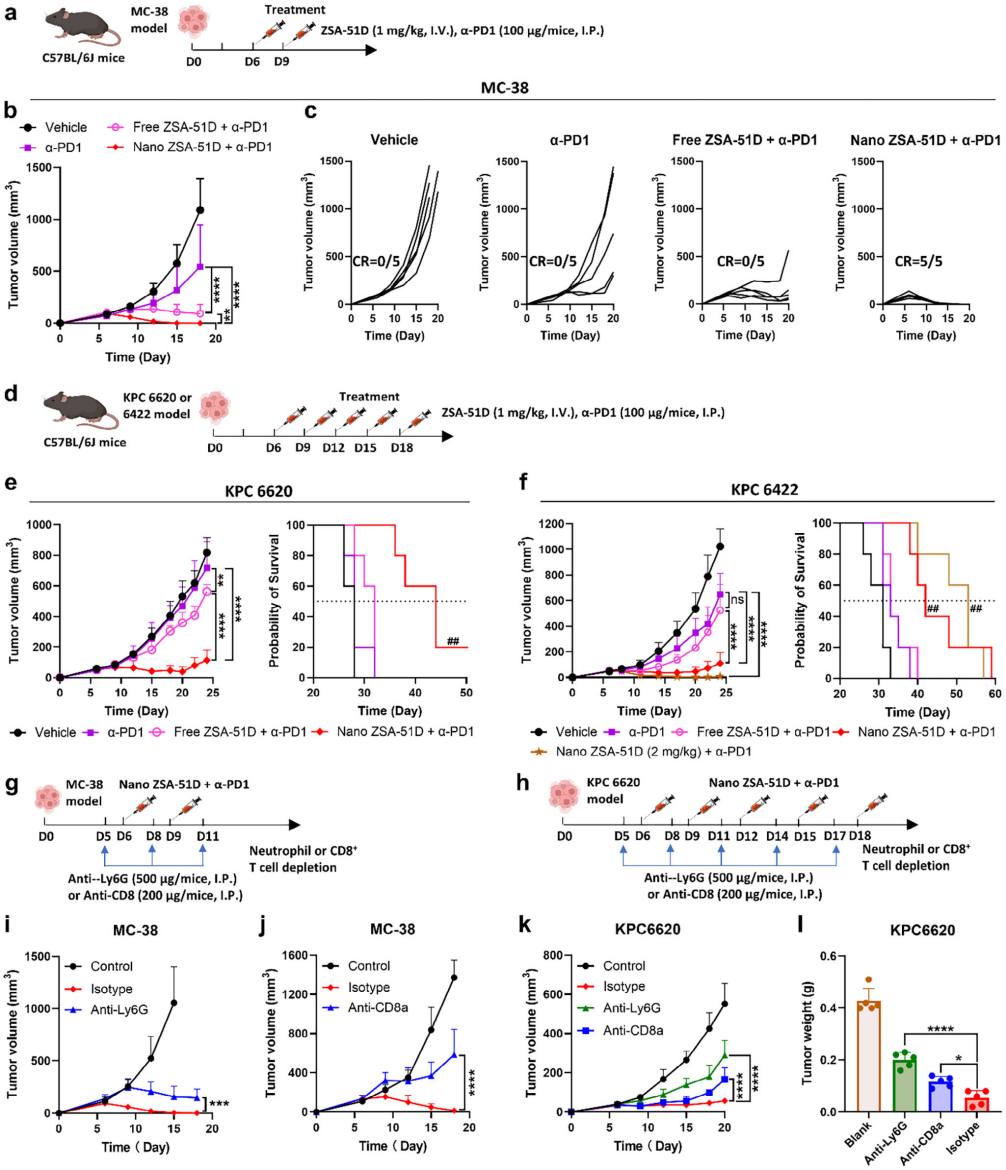
Nano ZSA‐51D synergizes with α‐PD1 to achieve potent efficacy in colon and pancreatic cancers through neutrophil‐ and CD8^+^ T cell‐dependent mechanisms. (a) Schematic Illustration of the MC‐38 colon cancer model establishment in C57BL/6J mice and treatment regimen in tumor‐bearing mice. (b‐c) Tumor growth curves (b) and individual tumor growth trajectories (c) of MC‐38 colon cancer model following the indicated treatment regimens (*n* = 5, mean ± SD). Two‐way ANOVA with Sidak's tests: **p < 0.01, ****p < 0.0001. (d) Schematic Illustration of the KPC 6620 and KPC 6422 pancreatic cancer model establishment in C57BL/6J mice and treatment regimen in tumor‐bearing mice. (e‐f) Tumor growth curves (left) and Kaplan‐Meier survival curves (right) of KPC 6620 (e) and KPC 6422 (f) pancreatic cancer model following the indicated treatments (*n* = 5, mean ± SD). Tumor growth was analyzed using two‐way ANOVA with Tukey's tests: **p < 0.01, ****p < 0.0001; ns: not significant. Survival was analyzed by log‐rank (Mantel–Cox) test: ##*p* < 0.01 vs α‐PD1 therapy. (g‐h) Schematic illustration of the indicated treatment regimens with neutrophil or CD8^+^ T‐cell depletion in the MC‐38 colon (g) and KPC 6620 pancreatic cancer (h) models. Anti‐Ly6G (500 µg) or anti‐CD8a (200 µg) antibodies were administered intraperitoneally 1 day before each treatment of Nano ZSA‐51D (1 mg/kg, I.V.) combined with α‐PD1 (100 µg, I.P.). (i‐k) Tumor growth curves of the MC‐38 colon (i, j) and KPC 6620 pancreatic (k) cancer models following Nano ZSA‐51D and α‐PD1 combination treatment with neutrophil or CD8^+^ T‐cell depletion (*n* = 5, mean ± SD). Two‐way ANOVA with Tukey's tests: ***p < 0.001, ****p < 0.0001. (l) Tumor weights of the KPC 6620 pancreatic (k) cancer models on day 20 following Nano ZSA‐51D and α‐PD1 combination treatment with neutrophil or CD8^+^ T‐cell depletion (*n* = 5, mean ± SD). One‐way ANOVA with Tukey's tests: *p < 0.05, ****p < 0.0001.

We next evaluated the therapeutic efficacy of Nano ZSA‐51D in combination with α‐PD1 therapy in MC‐38 colon cancer model (Figure 6a). Notably, Nano ZSA‐51D combined with α‐PD1 achieved 100% complete tumor remission (100% CR) without recurrence, leading to 100% long‐term survival after two doses (Figure 6b,c). In contrast, free ZSA‐51D combined with α‐PD1 significantly delayed tumor growth but failed to induce complete remission.

We further evaluated the efficacy of this combination in the KPC 6620 and KPC 6422 pancreatic cancer models, which are typically resistant to α‐PD1 therapy and derived from the KPC (Kras^G12D, Trp53^R172H, Pdx1‐Cre) genetically engineered mouse model (Figure 6d) [51]. In both models, Nano ZSA‐51D combined with α‐PD1 significantly reduced tumor burden and extend median survival compared with α‐PD1 monotherapy, whereas free ZSA‐51D provided no additional benefit (Figure 6e,f; Figure S14). Increasing the Nano ZSA‐51D dose to 2 mg/kg further enhanced tumor regression in KPC 6422 mice, extending median survival by approximately three weeks relative to α‐PD1 alone (Figure 6f; Figure S14). These results demonstrate that Nano ZSA‐51D effectively sensitizes otherwise resistant tumors to α‐PD1 therapy.

To elucidate the mechanism underlying this synergy, we depleted the neutrophils or CD8^+^ T cells using specific antibodies in MC‐38 and KPC6620 models (Figure 6g,h). Depletion of either neutrophils or CD8^+^ T cells markedly impaired the antitumor efficacy of Nano ZSA‐51D in combination with α‐PD1 therapy (Figure 6i–l), indicating that both neutrophils and CD8^+^ T cells are essential mediators of this combination's antitumor activity.

### 2.8 Nano ZSA‐51D‐Induced Neutrophil Infiltration Remodels TME to Promote CD8^+^ T Cell Immunity and Long‐Term Antitumor Memory

**FIGURE 7:**
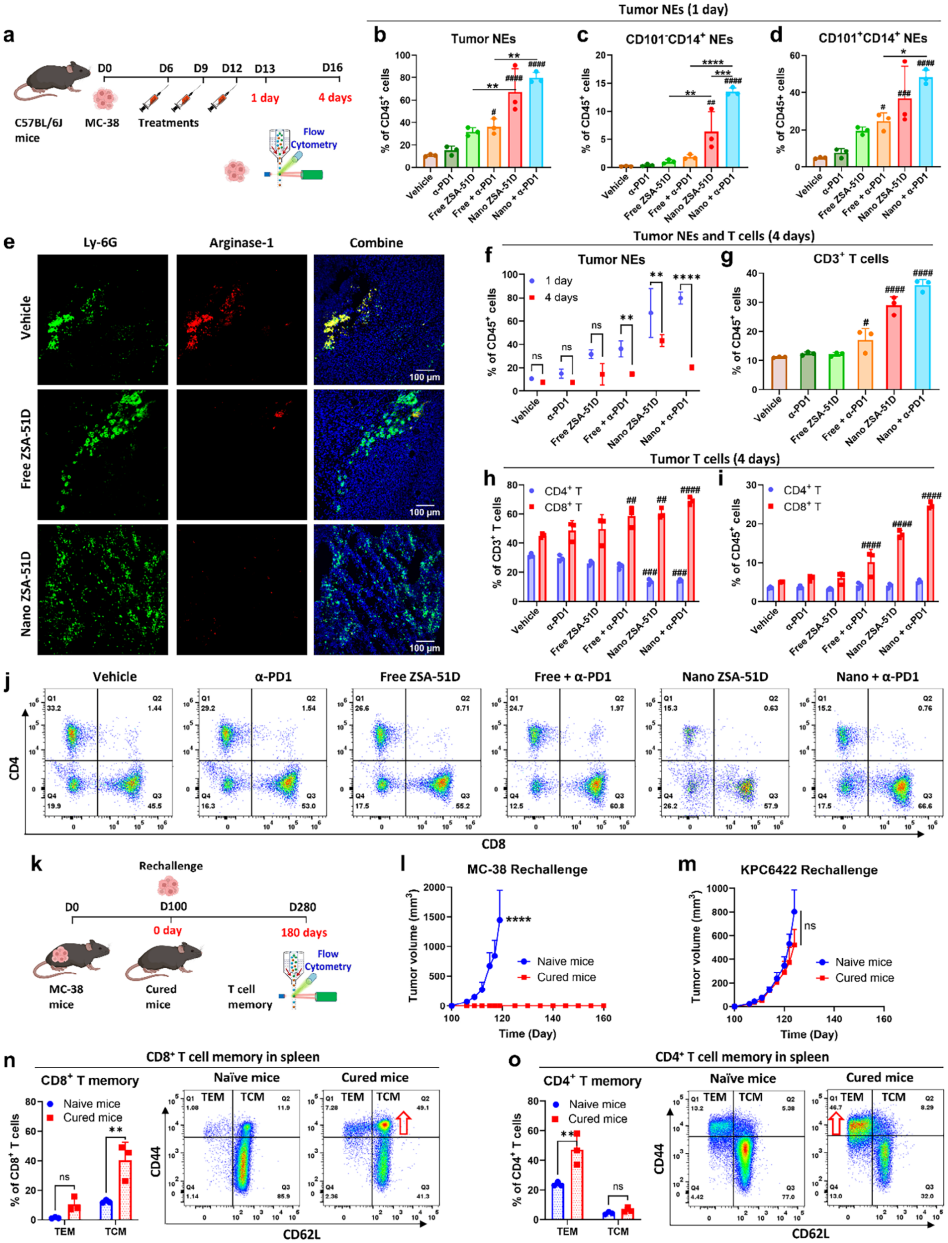
Nano ZSA‐51D‐induced neutrophil infiltration remodels TME to promote CD8^+^ T cell immunity and long‐term antitumor memory. (a) Schematic illustrating the treatment schedule and flow cytometry analysis of tumor‐infiltrating immune cells in MC‐38 tumor‐bearing C57BL/6J mice at 1 day and 4 days post‐treatment. (b‐d) Quantification of total tumor‐infiltrating neutrophils (NEs) (b), CD101^−^CD14^+^ (c) and CD101^+^CD14^+^ (d) NEs within CD45^+^ immune cells at 1‐day post‐treatment (*n* = 3, mean ± SD). One‐way ANOVA with Tukey's tests (*), or Dunnett's tests for comparisons versus the vehicle group (#): *p < 0.05, **p < 0.01, ***p < 0.001, ****p < 0.0001; #*p* < 0.05, ##*p* < 0.01, ###*p* < 0.001, ####*p* < 0.0001 vs. vehicle group. (e) Immunofluorescence analysis of neutrophil infiltration in MC‐38 tumors 1 day after treatment with vehicle, free or Nano ZSA‐51D. Scale bars, 100 mm. (f) Quantification and comparison of tumor‐infiltrating neutrophils within CD45^+^ immune cells at 1 day and 4 days post‐treatment (*n* = 3, mean ± SD). Two‐way ANOVA with Sidak's tests: **p < 0.01, ****p < 0.0001. (g‐i) Quantification of CD3^+^ T cells within CD45^+^ immune cells (g), CD4^+^ and CD8^+^ T cells within CD3^+^ T cells (h) or within CD45^+^ immune cells (i) at 4 days post‐treatment (*n* = 3, mean ± SD). (g) One‐way or (h, i) two‐way ANOVA with Dunnett's tests for comparisons versus the vehicle group: #*p* < 0.05, ##*p* < 0.01, ###*p* < 0.001, ####*p* < 0.0001. (j) Representative flow cytometry plots of tumor‐infiltrating CD4^+^ and CD8^+^ T cells within CD3^+^ T cells at 4 days post‐treatment. (k) Schematic of MC‐38 tumor cell rechallenge in mice cured with Nano ZSA‐51D and α‐PD1 combination therapy at 100 days post‐treatment. Long‐term T cell memory was evaluated by flow cytometry at 180 day post‐rechallenge. (l, m) Tumor growth curves following rechallenge with MC‐38 (l) or KPC 6422 (m) tumor cell in naïve C57BL/6J mice and cured mice at 100 days post‐treatment (*n* = 5, mean ± SD). Two‐way ANOVA with Sidak's tests: ****p < 0.0001; ns, not significant. (n, o) Quantification (left) and representative flow cytometry plots (right) of CD8^+^ (n) and CD4^+^ (o) effector memory (TEM: CD62L^−^CD44^+^) and central memory (TCM: CD62L^+^CD44^+^) T cells in the spleen of naïve C57BL/6J mice and cured mice at 180‐day post‐rechallenge (*n* = 3, mean ± SD). Two‐way ANOVA with Sidak's tests: **p < 0.01; ns, not significant.

To determine whether Nano ZSA‐51D‐induced neutrophils drive CD8^+^ T cell antitumor responses in vivo, we analyzed tumor‐infiltrating immune cells in MC‐38 tumors at 1‐ and 4‐day post‐treatment (Figure 7a).

At 1‐day post‐treatment, Nano ZSA‐51D combined with α‐PD1 dramatically increased tumor‐infiltrating neutrophils from 10.6% to 79.7% of CD45^+^ immune cells, compared to vehicle‐treated groups (Figure 7b; Figure S15A). Specifically, the immature/activated (CD101^−^CD14^+^) neutrophils expanded from 0.22% to 13.5% (Figure 7c; Figure S15B), and the mature/activated (CD101^+^CD14^+^) neutrophils expanded from 5% to 48.2% (Figure 7d; Figure S15B). These Nano ZSA‑51D–induced neutrophils infiltrated throughout MC‑38 tumors rather than being restricted to the tumor periphery (Figure 7e). Neutrophils from vehicle‐treated tumors exhibited high arginase‐1 expression, a hallmark of PMN‐MDSC–mediated immunosuppression. In contrast, arginase‐1 expression was markedly reduced in neutrophils from mice treated with free or Nano ZSA‐51D, indicating attenuation of the PMN‐MDSC–like phenotype. Concurrently, macrophages decreased from 59.7% to 9.1% of CD45^+^ cells (Figure S15C,D). A similar trend was observed in the KPC 6620 pancreatic cancer model, where treatment increased neutrophil infiltration from 31.9% to 63.3% (Figure S16A,C) and reduced macrophages from 55.1% to 21.7% (Figure S16B,D).

By 4 days post‐treatment, tumor‐infiltrating neutrophils declined, with the most substantial reduction observed from 79.7% to 20.4% in the Nano ZSA‐51D with α‐PD1 group (Figure 7f; Figure S17A). This reduction is consistent with the transient expansion of neutrophils following Nano ZSA‐51D treatment (Figure S18), where neutrophils rapidly increase within the first 24 h and gradually return toward baseline by day 3, accompanied by phenotypic changes. In parallel, tumor‐infiltrating T cells increased markedly, with the most dramatic increase from 11.1% to 35.9% of CD45^+^ immune cells in Nano ZSA‐51D with α‐PD1 group (Figure 7g; Figure S17B). Further analysis revealed a selective expansion of CD8^+^ T cells, while CD4^+^ T cell frequencies remained unchanged among CD45^+^ immune cells (Figure 7h–j). These results suggest that early infiltration of activated CD14^+^ neutrophils following Nano ZSA‐51D treatment promotes subsequent CD8^+^ T cell responses within TME.

To determine whether Nano ZSA‐51D with α‐PD1 therapy induces durable antitumor immunity, we rechallenged cured MC‐38 mice with MC‐38 or KPC6422 tumor cells 100 days after treatment (Figure 7k). Remarkably, cured mice completely rejected MC‐38 tumors but not KPC6422 tumors (Figure 7l,m), demonstrating tumor‐specific immune memory.

To further characterize the immune memory response, we also analyzed CD8^+^ and CD4^+^ T cell memory subsets in the spleen, lymph nodes and blood of cured MC‐38 mice 180 days after tumor rechallenge (Figure 7k). Flow cytometry analysis revealed a significant increase in CD8^+^ T central memory (TCM) cells in cured MC‐38 mice, with a 3.3‐, 4.4‐ and 4.5‐fold increase in spleen, lymph nodes and blood compared to naïve controls (Figure 7n; Figure S19A,C). While CD4^+^ TCM cells showed no significant changes (Figure 7o; Figure S19B,D). These results further confirm that Nano ZSA‐51D with α‐PD1 therapy generates the long term CD8^+^ T cell memory, which contributes to complete tumor remission and long‐term tumor immunity.

## 3 Conclusion

Although the immunosuppressive role of PMN‐MDSCs in cancer has been extensively studied over the past decade, developing strategies to overcome neutrophil‐mediated immune suppression remains challenging [1, 2, 9]. Neutrophils exhibit remarkable plasticity and context‐dependent functions, rapidly adapting to tumor‐derived signals, which complicates efforts to define their roles and develop effective interventions against PMN‐MDSC‐mediated immune suppression [5, 6, 7, 8].

Recent evidence suggests that the immunosuppressive functions of PMN‐MDSCs can be programmed early at the hematopoietic stem and progenitor cell (HSPC) stage by cancer‐derived signals [25, 26, 27, 28, 29, 30]. Given that downregulated interferon signaling underlies PMN‐MDSC development [32, 33], we found that activating the STING–interferon axis can reprogram HSPCs toward antitumor neutrophils for improved cancer immunotherapy. In this study, we developed an albumin‐bound STING agonist (Nano ZSA‐51D), which expands HSPCs and reprograms them to generate antitumor neutrophils with enhanced MHC I antigen presentation, thereby promoting CD8^+^ T cell‐mediated antitumor immunity and sensitizing tumors to α‐PD1 immunotherapy. Nano ZSA‐51D combined with α‐PD1 achieved potent therapeutic efficacy in colon and pancreatic cancer models through neutrophil‐ and CD8^+^ T‐cell–dependent mechanisms.

While previous studies have focused mainly on STING‐driven activation of dendritic cells and macrophages, our findings broaden this paradigm by reprograming HSPCs to antitumor neutrophils as key effector cells in STING‐based immunotherapy. We show that Nano ZSA‐51D activates STING‐interferon signaling in HSPCs, expands their population, and reprograms them toward granulocyte‐monocyte progenitors (GMPs), thereby expanding the neutrophil lineage. This suggests that STING signaling in the bone marrow is not merely an immune‐sensing pathway but also a regulator of hematopoietic fate of HSPCs [52]. In addition, Nano ZSA‐51D reprograms the transcriptional landscape of HSPCs toward an interferon‐simulated immune phenotype, thereby promoting differentiation into antitumor neutrophils rather than immunosuppressive PMN‐MDSCs. Nano ZSA‐51D may also work directly on peripheral and tumor‐infiltrating neutrophils, contributing to the TME remodeling by attenuating PMN‐MDSC–associated immunosuppressive features.

This study also reveals a previously underappreciated antitumor role of neutrophils in STING‐mediated cancer immunotherapy and elucidates the underlying mechanisms [53, 54, 55]. We demonstrate that Nano ZSA‐51D reprogrammed immature (CD101^−^) and mature (CD101^+^) neutrophils into CD14^+^ICAM‐1^+^ subsets via STING–NF‐κB–TNF‐α signaling. These CD14^+^ICAM‐1^+^ neutrophils infiltrate tumors efficiently and synergize with α‐PD1 therapy. Notably, adoptive transfer of Nano ZSA‐51D‐reprogrmamed immature CD14^+^ neutrophils combined with α‐PD1 led to 100% complete tumor remission and established long‐term CD8^+^ T cell central memory, highlighting their potent antitumor capacity. CD14 functions as a co‐receptor for pattern‐recognition receptors, activating innate immunity responses to pathogens by binding PAMPs and DAMPs [41, 56, 57, 58]. While neutrophil ICAM‐1 (CD54) enhances neutrophil adhesion, migration, and co‐stimulation of T cells [43, 44, 59, 60, 61]. Together, these molecules facilitate the tumor infiltration and antitumor activity of Nano ZSA‐51D–reprogrammed neutrophils.

Transcriptomic and immune profiling further reveal that Nano ZSA‐51D activates robust interferon signaling and upregulates MHC I antigen‐presentation pathways in CD14^+^ neutrophils, including MHC I molecules, costimulatory ligands, cytokines and chemokines, thereby supporting antigen‐specific CD8^+^ T cell‐mediated antitumor immunity. These features resemble ICAM‐1^hi^CD14^+^MHC I^hi^ APC‐like hybrid neutrophils recently identified in early‐stage human lung cancer, which exhibits antigen‐presenting properties and promotes T‐cell immunity [37, 38, 62]. In contrast, CD11b^+^CD14^−^ MDSCs suppress CD8^+^ T cell activation, driving immune evasion [63]. Our data demonstrate that Nano ZSA‐51D induces CD14^+^ICAM‐1^+^MHC I^hi^ neutrophils, acquiring antitumor properties through enhanced MHC I‐mediated antigen presentation to prime CD8^+^ T cell responses that bridges innate and adaptive immunity.

Despite the promise of STING agonists as cancer therapeutics, the clinical efficacy of cyclic dinucleotide (CDN)–based agents have been limited [64, 65, 66]. CDN‐based STING agonists suffer from rapid enzymatic degradation and poor cellular permeability due to their hydrophilic and anionic nature, restricting their use to intratumoral administration. To overcome these limitations, nanoparticle‐formulated CDNs and non‐CDN STING agonists have been developed to improve pharmacokinetics and enable systemic delivery [66, 67, 68, 69, 70, 71, 72]. The observed bone marrow enrichment of Nano ZSA‐51D may reflect physiological targeting driven by albumin biology, marrow sinusoidal permeability, or preferential uptake by myeloid‐lineage cells, which result in functionally relevant STING activation within bone marrow [73, 74]. Given the complexity of bone marrow trafficking and niche interactions, future studies employing cell‐specific tracking, receptor blocking, or genetic models will be required to fully elucidate the molecular basis of bone marrow enrichment. We also evaluated the toxicity profile of Nano ZSA‐51D by using single and multiple doses (5 doses, 1 mg/kg, I.V.). Complete blood cell count, liver functions, histopathological assessments and hemolysis tests revealed no significant toxicity (Figures S20 and S21), indicating that Nano ZSA‐51D is well tolerated. Future studies will evaluate the efficacy and safety of Nano ZSA‐51D in humanized immune system or patient‐derived xenograft models to further support its translational potential.

Collectively, these findings establish Nano ZSA‐51D as a novel STING agonist strategy to reprogram HSPCs toward antitumor neutrophils. These reprogrammed neutrophils infiltrate tumors, enhance MHC I antigen presentation, and prime robust CD8^+^ T‐cell responses. Combination with α‐PD1 therapy achieves potent anticancer efficacy in both colon and pancreatic cancer models through neutrophil‐ and CD8^+^ T cell‐dependent mechanisms. These results highlight the potential of early interventions at HSPC stage to rewire neutrophil fate for effective immunotherapy.

## 4 Experimental Section/Methods

### 4.1 Cell Cultures

All cells were maintained at 37°C in a humidified incubator (5% CO_2_). THP‐1‐Blue ISG cells (InvivoGen) were cultured in RPMI 1640 with 10% heat‐inactivated FBS and 100 µg/mL normocin. LSK cells from WT C57BL/6J mice were maintained in StemSpan medium with 50 ng/mL mouse SCF (BioLegend). Bone marrow neutrophils from WT and STING KO mice (C57BL/6J‐Sting1gt/J) and CD8^+^ T cells from OT‐I mice were cultured in RPMI 1640 with 10% FBS. MC‐38 colon adenocarcinoma cells (Kerafast) were cultured in DMEM with 10% FBS and gentamycin. KPCY pancreatic cancer cells (KPC 6620 and KPC 6422; Kerafast) were cultured in DMEM with GlutaMAX, 10% FBS, and sodium pyruvate. Unless otherwise indicated, all media contained standard supplements (L‐glutamine, HEPES, sodium pyruvate, nonessential amino acids, and Pen/Strep). All cell lines were tested negative for mycoplasma contamination. Key reagents are listed in Table S1.

### 4.2 Mice and Tumor Models

All animal experiments were approved (PRO00011365) by the Institutional Animal Care and Use Committee (IACUC) of the University of Michigan and conducted in accordance with the National Institutes of Health (NIH) guidelines for the care and use of laboratory animals. WT C57BL/6J, STING KO mice (C57BL/6J‐Sting1gt/J), and OT‐I transgenic (C57BL/6‐Tg(TcraTcrb)1100Mjb/J) female mice (6–8 weeks old) were used.

For tumor implantation, 1 × 10^6^ MC38, KPC 6620, or KPC 6422 cells in 100 µL FBS‐free DMEM were injected subcutaneously into the right flank. All experiments were conducted with randomized group allocation and blind analysis to minimize experimental bias. Mice were randomized for treatment when tumors reached 50–100 mm^3^, and tumor growth was monitored every 2–3 days. Tumor volume = length × width^2^/2. Survival analysis was performed using Kaplan–Meier curves. Mice were euthanized and recorded as death events when tumor volumes exceeded 2000 mm^3^ for MC38 tumors or 1500 mm^3^ for KPC tumors, in accordance with predefined humane endpoints.

Humane endpoints were strictly followed in accordance with NIH guidelines and IACUC‐approved protocols. Mice were euthanized if end‐stage illness scores were reached, or if ulceration, impaired mobility, or signs of excessive distress were observed. All animals were closely monitored to ensure welfare.

### 4.3 Synthesis and Characterization of Dimeric STING Agonist ZSA‐51D

The dimeric STING agonist ZSA‐51D was synthesized from our previously reported monomeric agonist ZSA‐51 (Supplementary Methods and Figure S1) [31, 69]. Briefly, starting material **1** was reacted with MeMgBr in Et_2_O at 0°C to yield intermediate **2**, which was oxidized with PCC in DCM to give **3**. Intermediate **3** was coupled with methyl thioglycolate and DIPEA in DMA, followed by t‐BuOK treatment at 80°C to produce **4**. Compound **4** was amidated with methyl 3‐aminopropionate HCl or tert‐butyl 3‐aminopropionate HCl using HATU/DCM/DIPEA, yielding **5a/b**. Hydrogenation with Pd/C in ethanol afforded **6a/b**, which was silylated with TBDMSCl/imidazole/DMAP to form **7a/b**. Subsequent bromination (NBS/AIBN/CCl_4_, 80°C) produced **8a/b**, oxidized to **9a/b** with NMO, then to **10a/b** with PCC. Deprotection with TBAF/THF gave **11a/b**. Dimerization of 11a/b with 1,3‐dibromopropane in CH_3_CN/K_2_CO_3_/NaI at 80°C yielded **ZSA‐51D** (or **13b**). Further deprotection of 13b with TFA/DCM produced **ZSA‐52D**. The structure and purity were confirmed by ^1^H NMR and LC–MS.

### 4.4 hSTING Binding and THP1‐Blue ISG Cell Reporter Assay

Binding affinity for human STING was measured using an HTRF assay (Revvity). Test compounds (5 µL) were mixed with human STING WT protein (5 µL, 6×His‐tagged), followed by 10 µL of premixed d2 ligand and Tb cryptate‐conjugated antibody in 386‐well plates for 3 h at room temperature. HTRF signals were measured using a Synergy 2 microplate reader (BioTek).

For STING activation, THP‐1‐Blue ISG cells were treated with test compounds (100 nm) for 24 h. SEAP activity in supernatants was quantified using QUANTI‐Blue reagent (InvivoGen) at 620 nm. All assays were run in triplicate.

### 4.5 Nano ZSA‐51D Preparation

Nano ZSA‐51D was prepared as previously described [33]. Briefly, 10 mg ZSA‐51D in 1 mL chloroform was emulsified with 100 mg mouse serum albumin in 20 mL water using a rotor‐stator homogenizer. The emulsion was processed through six cycles at 20 000 psi with a high‐pressure homogenizer (4°C), and chloroform was removed under reduced pressure at 25°C. The resulting suspension was lyophilized and stored at −20°C. Particle size was measured by dynamic light scattering (DLS; Zetasizer Nano‐ZS, Malvern), with three measurements per sample and experiments performed in triplicate. TEM (Talos, STEM, 370 k×) was conducted following 2% phosphotungstic acid negative staining.

### 4.6 Western Blot Analysis

Bone marrow cells from C57BL/6 mice were treated with vehicle, free, or Nano ZSA‐51D (10 or 100 nm) for 4 h, lysed in RIPA buffer with protease and phosphatase inhibitors, sonicated, and centrifuged at 12 000 × g for 15 min at 4°C. Protein concentrations were determined by BCA assay. Equal amounts of protein were separated by SDS‐PAGE, transferred to PVDF membranes, blocked with 5% milk in TBST, and incubated with primary antibodies against STING, p‐STING (Ser365), IRF3, p‐IRF3 (Ser396), NF‐κB p65, and p‐NF‐κB p65 (Ser536), followed by HRP‐conjugated secondary antibodies. Signals were detected using ECL (Bio‐Rad) and imaged with a ChemiDoc MP system.

### 4.7 In Vivo Treatment Regimen

Mice were intravenously administered free or Nano ZSA‐51D (1 mg/kg) or vehicle (15 mg/kg mouse serum albumin). Free ZSA‐51D was dissolved in DMSO/PEG‐400 (1:4, v/v) and diluted 1:1 with PBS to 0.2 mg/mL for injection; Nano ZSA‐51D was directly resuspended in PBS at 0.2 mg/mL. For combination therapy, 100 µg α‐PD1 antibodies (Bio X Cell) were given intraperitoneally according to the experimental schedule. For the depletion of neutrophils or CD8^+^ T cells, mice with tumors were intraperitoneally administered anti‐Ly6G (1A8, 500 µg) or anti‐CD8a (YTS 169.4, 200 µg) antibodies 1 day before each treatment with Nano ZSA‐51D.

### 4.8 In Vivo PK Study

MC‐38 tumor‐bearing mice received free or Nano ZSA‐51D (1 mg/kg, i.v.). Blood and tissues were collected at 15 min, 2 h, and 7 h post‐injection. Plasma and homogenized tissues (tumor, bone marrow) were analyzed by LC–MS/MS (Triple Quad 5500, SCIEX) using analyte‐specific MRM transitions. Data were reported as ng/mL (plasma) or ng/g (tissue).

### 4.9 Single Cell Suspension Preparation and Flow Cytometry Analysis

Peripheral blood, bone marrow, and tumors were collected after STING agonist treatment. Blood (200 µL) was drawn from ophthalmic veins, and tumors and hind limb bones were harvested post‐euthanasia. Bone marrow was flushed with ice‐cold PBS + 1% FBS and 1 mM EDTA through a 70 µm strainer and centrifuged (350 × g, 5 min). Tumors (<1 g) were minced, digested in RPMI 1640 with 0.01%–0.1% collagenase/hyaluronidase and 0.15 mg/mL DNase I at 37°C for 30 min with shaking, filtered, and washed. All suspensions underwent RBC lysis (ACK buffer, 5 min, 4°C), washed, centrifuged (400 × g, 5 min), and resuspended at 1 × 10^7^ cells/mL. Cells were stained with Ghost Dye Violet 510 (30 min, 4°C), blocked with anti‐CD16/32 (10 min, 4°C), then stained with fluorochrome‐conjugated antibodies (Table S1) for 20 min at 4°C, washed, fixed (BioLegend), and analyzed on a Cytek Aurora cytometer; data were processed using FlowJo (Figure S22). For HSPC analysis, bone marrow single‐cell suspensions were enriched for lineage‐negative (Lin^−^) cells using a biotin‐conjugated lineage panel (BioLegend) and magnetic separation, then stained with fluorophore‐conjugated antibodies (Table S1) for flow cytometric analysis of HSPC subsets (Figure S23).

### 4.10 Cell Sorting by FACS

Neutrophils and subpopulations were isolated from bone marrow treated in vitro with vehicle or Nano ZSA‐51D (100 nm) using a Sony MA900 sorter with a 100 µm chip at 4°C. Cells were stained with CD11b, Ly6G, CD101, and CD14, and dead cells excluded with DAPI (5 µg/mL). Neutrophils (CD11b^+^Ly6G^+^) were further gated by CD101 and CD14 to yield CD101^−^CD14^−^, CD101^−^CD14^+^, and CD101^+^CD14^+^ populations. Sorted cells were used for in vitro assays, adoptive transfer, or bulk mRNA analysis. LSK cells (Lin^−^Sca‐1^+^c‐Kit^+^) were sorted from enriched bone marrow Lin^−^ cells.

### 4.11 LSK Culture and Treatment

Sorted LSK cells were cultured in StemSpan with 50 ng/mL SCF (BioLegend) at 2 × 10^4^ cells/well in 96‐well U‐bottom plates. Cells were treated with 100 nm Nano ZSA‐51D for 3 days, then collected, washed, and resuspended in PBS for flow cytometry or bulk mRNA‐seq analysis.

### 4.12 In Vitro Reprogramming of Bone Marrow Neutrophils

Bone marrow cells were isolated from femurs and tibiae of WT or STING KO mice (6–8 weeks). Cells were flushed with PBS + 1% FBS and 1 mm EDTA, centrifuged, RBC‐lysed, washed, and resuspended in complete RPMI 1640. Cells (5 × 10^6^/well) were seeded in 12‐well plates and treated overnight with 100 nm free or Nano ZSA‐51D, vehicle (30 µg/mL mouse serum albumin). In addition, the sorted bone marrow neutrophils were treated with 10 ng mL^−1^ cytokines (TNF‐α, IFN‐β, IFN‐γ, IL‐6, TGF‐β) for overnight. For TNF‐α neutralization, cells were pretreated with TNF‐α antibody (10 µg/mL) 2 h in advance of Nano ZSA‐51D treatment. After treatment, cells were collected, washed, and resuspended in PBS for flow cytometry (Figure S24).

### 4.13 Adoptive Transfer of Neutrophil Subsets

Sorted neutrophil subsets (CD101^−^CD14^−^, CD101^−^CD14^+^, CD101^+^CD14^+^; 2 × 10^6^ cells/mouse) from Nano ZSA‐51D–treated bone marrow was adoptively transferred intratumorally into MC‐38 tumors, and mice received intraperitoneal α‐PD1 (100 µg) every 2 days. Tumor growth and survival were monitored every 2–3 days.

### 4.14 Tumors Rechallenge and T Cell Memory Analysis

To assess long‐term immunity, cured mice were rechallenged 100–120 days post‐treatment with MC‐38 or KPC 6642 cells (5 × 10^6^ cells/mL, 100 µL) subcutaneously, alongside age‐matched naïve controls. Tumor growth was monitored every 2–3 days from day 6, and tumor volume was recorded. At 180 days post‐rechallenge, spleens, lymph nodes, and blood were collected to prepare single‐cell suspensions for flow cytometric analysis of CD4^+^ and CD8^+^ T cell memory (Figure S25).

### 4.15 Bulk mRNA‐Seq Analysis

Total RNA was extracted (RNeasy Plus, QIAGEN), and samples with RIN ≥ 8 were used. Poly(A) mRNA (250 ng) was isolated (NEBNext Poly(A) Magnetic Isolation), converted to cDNA libraries (NEBNext UltraExpress RNA Library Prep with dual indices), pooled, and sequenced on Illumina NovaSeq X 10B (150 bp paired end). Adapters and low‐quality bases were trimmed with Cutadapt, quality checked with FastQC, and contamination screened with Fastq Screen. Reads were aligned to GRCm38 (ENSEMBL) using STAR, quantified with RSEM, and metrics summarized with MultiQC. Differential expression was analyzed using DESeq2; genes with adjusted *p* < 0.05 and |log_2_FC| > 1 were considered significant. KEGG pathway ORA and GSEA using KEGG/GO gene sets were performed for functional enrichment.

### 4.16 Antigen Presentation by Neutrophils

Bone marrow cells from WT C57BL/6J mice were seeded at 1 × 10^6^ cells/well in 24‐well plates and treated overnight with OT‐I peptide (SIINFEKL, 50 µg/mL), Nano ZSA‐51D (100 nm), or vehicle (30 µg/mL mouse serum albumin) in complete RPMI 1640. Cells were collected, washed, and stained with antibodies. MHC I expression and OT‐I peptide presentation (H‐2Kb/SIINFEKL) on neutrophils and subsets were analyzed by flow cytometry.

### 4.17 OT‐I CD8^+^ T Cell Activation and Proliferation

OT‐I CD8^+^ T cells were isolated (EasySep CD8^+^ kit, STEMCELL), labeled with CellTrace Violet, and washed. Bone marrow neutrophils or subsets (CD101^−^CD14^+^, CD101^+^CD14^+^) pretreated with OVA (200 µg/mL), Nano ZSA‐51D (100 nm), or vehicle were sorted and co‐cultured with labeled T cells at a 2:1 neutrophil/T cell ratio for 48 h. T cells alone served as controls. Cells were imaged by bright‐field microscopy (EVOS M5000) and analyzed by flow cytometry for activation markers (CD69, CD25, CD137) and proliferation via CellTrace dilution.

### 4.18 IFN‐γ ELISPOT Assay

Bone marrow neutrophils or subset (CD14^+^ICAM‐1^+^MHC I^^hi^) pretreated with vehicle or Nano ZSA‐51D (100 nm) were sorted and plated in IFN‐γ ELISPOT plates with or without OVA (200 µg/mL). OT‐I CD8^+^ T cells were then added at a 2:1 neutrophil/T cell ratio and co‐cultured for 24 h at 37°C with 5% CO_2_. T cells alone served as controls. After incubation, plates were processed according to the manufacturer's instructions. IFN‐γ–producing cells were visualized as spot‐forming units and quantified using an ELISPOT reader.

### 4.19 Immunofluorescence and Confocal Imaging

Frozen tumor sections (10 µm) were fixed in 4% PFA/PBS for 10 min, blocked with 2% BSA, and stained AF700 anti‐mouse Ly‐6G for surface, then permeabilized (0.1% Triton X‐100) and PE anti‐mouse Arginase‐1 for intracellular, with DAPI nuclear counterstain. Images were acquired on a Nikon W1‐SoRa confocal (405, 561, 640 nm) and processed in NIS‐Elements/Fiji with identical settings across groups.

### 4.20 In Vivo Toxicity Assessment

Mice (*n* = 3) received vehicle, free, or Nano ZSA‐51D (1 mg/kg, i.v.) every 3 days for five doses. Blood was collected for CBC and liver function tests, and organs (heart, liver, spleen, lung, kidney, intestine) were examined histologically (ULAM, University of Michigan). Mouse hemolysis assay was performed by incubating mouse red blood cells with Nano ZSA‐51D. PBS and 0.1% Triton X‐100 were used as negative and positive controls, respectively. Absorbance was measured at 540 nm, and hemolysis was calculated as: Hemolysis (%) = (OD_sample − OD_PBS) / (OD_Triton − OD_PBS) × 100.

### 4.21 Statistical Analysis

Data are presented as mean ± standard deviation (SD). Sample sizes (n), defined as biologically independent samples, are indicated in the figure legends. No data was excluded from the analysis. Data was assessed for outliers using GraphPad Prism, and no outliers were removed unless explicitly stated. No data transformation or normalization was performed unless otherwise indicated.

Statistical analyses were performed using GraphPad Prism 10.3.1. Comparisons between two groups were conducted using unpaired two‐tailed Student's *t*‐tests. Comparisons among multiple groups were performed using one‐way or two‐way analysis of variance (ANOVA), followed by Tukey's test for pairwise comparisons among all groups, Dunnett's test for comparisons with a single control, or Sidak's test for predefined comparisons, as appropriate. Survival curves were analyzed using the Kaplan–Meier method, and statistical significance was determined by the log‐rank (Mantel–Cox) test. A *p* < 0.05 was considered significant. Statistical significance in figures is indicated as: *p* < 0.05 (*; #), *p* < 0.01 (**; ##), *p* < 0.001 (***; ###), *p* < 0.0001 (****; ####).

## Author Contributions

J.T., W.G., and D.S. conceived the project, developed the hypotheses, designed the experiments, and wrote the manuscript; J.T. performed most experiments and analyzed data, with assistance from all authors; H.Z. conducted the chemical synthesis. C.L. provided support for bioinformatic analysis. H.W. and F.K. assisted with the in vivo flow cytometry studies. Q.L., M.H., and B.W. assisted with the in vivo pharmacokinetic studies. Z.L. tested the STING binding and cellular activity in vitro. K.S. took the TEM image. D.S. supervised the project and acquired funding.

## Conflicts of Interest

University of Michigan has filed a patent based on these studies where some authors are listed as inventors. The authors declare no potential conflicts of interest.

## Supporting information




**Supporting File**: advs74790‐sup‐0001‐SuppMat.docx.

## Data Availability

All data supporting the findings of this study are available within the article and its Supporting Information or from the corresponding author upon reasonable request.
