# Supplementary material for: Albumin‐Bound STING Agonist Reprograms HSPCs to Antitumor Neutrophils Enhancing CD8+ T Cell Immunity
Source: Adv Sci (Weinh). 2026 Mar 19;13(29):e23603. doi: 10.1002/advs.202523603 (PMC13205613; doi:10.1002/advs.202523603)
Supplement: Supplementary file 1 — Supporting File: advs74790‐sup‐0001‐SuppMat.docx. [file ADVS-13-e23603-s001.docx]

**Supporting information**

Albumin-Bound STING Agonist Reprograms HSPCs to Antitumor Neutrophils Enhancing CD8⁺ T Cell Immunity

Jinsong Tao*, Hong-Yi Zhao, Chengyi Li, Hanning Wen, Fang Ke, Qiuxia Li, Miao He, Bo Wen, Zhongwei Liu, Kai Sun, Wei Gao*, Duxin Sun*

*Correspondence: [jinsongt@umich.edu](mailto:jinsongt@umich.edu) (J.T.), [wgao21@central.uh.edu](mailto:wgao21@central.uh.edu) (W.G.), [duxins@umich.edu](mailto:duxins@umich.edu) (D.S.)

**Table of Contents**

**Supplementary Methods.** Synthesis and characterization of ZSA-51D and ZSA-52D

**Figure S1.** Characterization of ZSA-51D and ZSA-52D.

**Figure S2.** Representative TEM image of Nano ZSA-51D.

**Figure S3.** Pharmacokinetic profiles of Nano ZSA-51D.

**Figure S4.** Nano ZSA-51D activates STING signaling pathway in bone marrow cells.

**Figure S5.** Nano ZSA-51D expands HSPCs and skews differentiation toward GMP Cells.

**Figure S6.** Nano ZSA-51D reprograms neutrophils into activated CD14⁺ subsets.

**Figure S7.** Nano ZSA-51D-mediated reprogramming of CD14^+^ICAM-1^+^ neutrophils is STING-dependent.

**Figure S8.** Nano ZSA-51D reprograms bone marrow neutrophils into CD14⁺ICAM-1^+^ subsets via STING–NF-κB–TNF-α signaling.

**Figure S9.** Antitumor efficacy after adoptive transfer of neutrophil subtypes with α-PD1 therapy.

**Figure S10.** Adoptive transfer of CD101^-^CD14⁺ neutrophils with α-PD1 therapy generates long-term CD8^+^ T cell memory.

**Figure S11.** Transcriptomic analysis of Nano ZSA-51D-reprogrammed bone marrow neutrophils.

**Figure S12.** Nano ZSA-51D-reprogrammed neutrophils prime CD8^+^ T cell responses.

**Figure S13.** Nano ZSA-51D-reprogrammed neutrophils displayed limited capacity to stimulate OT-II CD4⁺ T cells.

**Figure S14.** Antitumor efficacy of Nano ZSA-51D in KPC 6620 and 6422 pancreatic cancers.

**Figure S15.** Infiltration of neutrophils and macrophages in MC-38 at 1-day post-treatment.

**Figure S16.** Infiltration of neutrophils and macrophages in KPC 6620 at 1-day post-treatment.

**Figure S17.** Infiltration of neutrophils and T cells in MC-38 tumor at 4-day post-treatment.

**Figure S18.** Dynamic kinetics of circulating neutrophils following STING agonist treatment.

**Figure S19.** Memory T cell responses in cured mice by Nano ZSA-51D with α-PD1 therapy.

**Figure S20.** Nano ZSA-51D showed minimal toxicity following single or multiple-dose treatment.

**Figure S21.** Mouse hemolysis assay of Nano ZSA-51D.

**Figure S22.** Flow cytometric gating strategies of tumor infiltrating immune cells.

**Figure S23.** Flow cytometric gating strategies of HSPCs in bone marrow.

**Figure S24.** Flow cytometric gating strategies of neutrophil subsets.

**Figure S25.** Flow cytometric gating strategies of T cell memory subsets.

**Table S1.** Key reagent or resource table

**Supplementary Methods**

**Chemistry**

**1. Synthesis and characterization of ZSA-51D and ZSA-52D**

**Scheme S1. Synthetic scheme of ZSA-51D and ZSA-52D.**

**Synthesis of intermediate 2**

**1** (2.003 g, 6.23 mmol) was dissolved in anhydrous THF (20 mL). The solution was cooled to 0°C before MeMgBr (4 mL, 3 M in diethyl ether) was added dropwise. The mixture allowed to warm to room temperature and stirred overnight. Saturated NH_4_Cl (aq, 5 mL) was added dropwise with the cooling of an ice bath to quench the reaction followed by the addition of water (20 mL). The water phase was extracted by EA (3×20 mL). The organic phase was washed with saturated brine, dried with anhydrous Na_2_SO_4_, and evaporated under reduced pressure to give yellow oil which was used directly in the next step.

1-(5-(benzyloxy)-2-bromo-4-methoxyphenyl)ethan-1-ol (**2**)

Yellow oil, 80% yield, MS: calcd for C_16_H_18_BrO_3_ (M+H), 337.0; found, 337.3 (^79^Br), 339.2 (^81^Br).

**Synthesis of intermediate 3**

PCC (2.430 g, 11.28 mmol) was added to the solution of **2** (1.902 g, 5.64 mmol) in DCM (15 mL). The mixture was stirred at room temperature overnight. The solvent was removed under reduced pressure. The residue was isolated using flash chromatography (hexanes:EA = 10:1~5:1) to give desired product.

1-(5-(benzyloxy)-2-bromo-4-methoxyphenyl)ethan-1-one (**3**)

Yellow solid, 76% yield, MS: calcd for C_16_H_16_BrO_3_ (M+H), 335.0; found, 335.3 (^79^Br), 337.3 (^81^Br).

**Synthesis of intermediate 4**

To a flask were added **3** (1.790 g, 5.34 mmol), DMA (10 mL), DIPEA (5.34, 930 µL) and methyl thioglycolate (955 µL, 10.68 mmol). The mixture was stirred at room temperature under nitrogen atmosphere for 40 min. Then, *t*-BuOK (8.01, 0.898 g) was added. The resulted mixture was heated at 80°C under nitrogen atmosphere for 14 h. The solvent was removed under vacuum. The residue was dispersed into water (15 mL), extracted by DCM (3×15 mL). The solid formed in the process of extraction was filtered. Then, the pH of the water phase was adjusted to 2 using HCl (aq, 3 M). The precipitate was filtered and washed with water. This filter cake was combined with the previous filter cake and dried to give gray solid.

5-(benzyloxy)-6-methoxy-3-methylbenzo[*b*]thiophene-2-carboxylic acid (**4**)

Gray solid, 23% yield, MS: calcd for C_18_H_17_O_4_S (M+H), 329.1; found, 329.2.

**Synthesis of intermediate 5a~b**

To a flask were added **4** (0.105 g, 0.32 mmol), methyl 3-aminopropionate hydrochloride (58.1 mg, 0.42 mmol), HATU (0.182 g, 0.48 mmol), DCM (2 mL) and DIPEA (160 µL, 0.93 mmol). The mixture was stirred at room temperature for 2 h. Then mixture was diluted with DCM (50 mL), washed with water (3×15 mL), and evaporated under reduced pressure. The residue was triturated in the mixed solvent of EtOH (1 mL) and H_2_O (1 mL). The precipitate was filtered, washed with EtOH:H_2_O (1:1), and dried to give **5a**.

Methyl 3-(5-(benzyloxy)-6-methoxy-3-methylbenzo[*b*]thiophene-2-carboxamido)propanoate (**5a**)

Yellow solid, 55% yield, MS: calcd for C_22_H_24_NO_5_S (M+H), 414.1; found, 414.2.

*Tert*-butyl 3-(5-(benzyloxy)-6-methoxy-3-methylbenzo[*b*]thiophene-2-carboxamido)propanoate (**5b**)

Yellow solid, 72% yield, MS: calcd for C_25_H_30_NO_5_S (M+H), 456.2; found, 456.2.

**Synthesis of intermediate 6a~b**

To a flask were added **5a** (0.402 g, 0.97 mmol), Pd/C (5%, 80.1 mg) and EtOH (5 mL). The mixture was heated at 65°C under hydrogen gas atmosphere for 16 h before it was filtered through a pad of celite and washed with DCM:MeOH. The filtrate was evaporated under reduced pressure to give **6a** without further purification.

Methyl 3-(5-hydroxy-6-methoxy-3-methylbenzo[*b*]thiophene-2-carboxamido)propanoate (**6a**)

Gray solid, 95% yield, MS: calcd for C_15_H_18_NO_5_S (M+H), 324.1; found, 324.2.

*Tert*-butyl 3-(5-hydroxy-6-methoxy-3-methylbenzo[*b*]thiophene-2-carboxamido)propanoate (**6b**)

Gray solid, 92% yield, MS: calcd for C_18_H_24_NO_5_S (M+H), 366.1; found, 366.2.

**Synthesis of intermediate 7a~b**

To the solution of **6a** (0.244 g, 0.71 mmol) in DCM (4 mL) were added TBDMSCl (0.213 g, 1.42 mmol), imidazole (0.145 g, 2.13 mmol) and DMAP (8.7 mg, 0.071 mmol). After 0.5 h stirring at room temperature, the solvent was removed. The residue was isolated using flash chromatography (hexanes:EA = 5:1~3:1) to give **7a**.

Methyl 3-(5-((*tert*-butyldimethylsilyl)oxy)-6-methoxy-3-methylbenzo[*b*]thiophene-2-carboxamido)propanoate (**7a**)

Yellow oil, 86% yield, MS: calcd for C_21_H_32_NO_5_SSi (M+H), 438.2; found, 438.4.

*Tert*-butyl 3-(5-((*tert*-butyldimethylsilyl)oxy)-6-methoxy-3-methylbenzo[*b*]thiophene-2-carboxamido)propanoate (**7b**)

Yellow oil, 81% yield, MS: calcd for C_24_H_38_NO_5_SSi (M+H), 480.2; found, 480.4.

**Synthesis of intermediate 8a~b**

To a flask were added **7a** (0.190 g, 0.43 mmol), NBS (0.116 g, 0.65 mmol), AIBN (14.1 mg, 0.086 mmol) and CCl_4_ (4 mL). The mixture was heated at 80°C for 2 h. The solvent was removed. The residue was isolated using flash chromatography (hexanes:EA = 3:1~2:1) to afford **8a**.

Methyl 3-(3-(bromomethyl)-5-((*tert*-butyldimethylsilyl)oxy)-6-methoxybenzo[*b*]thiophene-2-carboxamido)propanoate (**8a**)

Yellow oil, 73% yield, MS: calcd for C_21_H_31_BrNO_5_SSi (M+H), 516.1; found, 516.3 (^79^Br), 518.3 (^81^Br).

*Tert*-butyl 3-(3-(bromomethyl)-5-((tert-butyldimethylsilyl)oxy)-6-methoxybenzo[*b*]thiophene-2-carboxamido)propanoate (**8b**)

Yellow oil, 61% yield, MS: calcd for C_24_H_37_BrNO_5_SSi (M+H), 558.1; found, 558.3 (^79^Br), 560.3 (^81^Br).

**Synthesis of intermediate 9a~b**

To the solution of **8a** (0.164 g, 0.32 mmol) in CH_3_CN (2 mL) was added NMO (0.112 g, 0.96 mmol) with the cooling of an ice bath. After 10 min, the ice bath was removed, and the mixture was stirred at room temperature overnight. The solvent was removed under reduced pressure. The residue was isolated using flash chromatography (hexanes:EA = 2:1~1:1) to furnish crude **9a**.

Methyl 3-(7-((*tert*-butyldimethylsilyl)oxy)-1-hydroxy-6-methoxy-3-oxo-1,3-dihydro-2*H*-benzo[4,5]thieno[2,3-*c*]pyrrol-2-yl)propanoate (**9a**)

This product is not stable. Yellow oil, 81% crude yield, MS: calcd for C_21_H_30_NO_6_SSi (M+H), 452.1; found, 452.3.

*Tert*-butyl 3-(7-((tert-butyldimethylsilyl)oxy)-1-hydroxy-6-methoxy-3-oxo-1,3-dihydro-2*H*-benzo[4,5]thieno[2,3-*c*]pyrrol-2-yl)propanoate (**9b**)

This product is not stable. Yellow oil, 61% crude yield, MS: calcd for C_24_H_36_NO_6_SSi (M+H), 494.2; found, 494.3.

**Synthesis of intermediate 10a~b**

To the solution of **9a** (0.120 g, 0.26 mmol) in DCM (2 mL) was added PCC (0.112 g, 0.52 mmol). The mixture was stirred at room temperature for 16 h. The solvent was removed. The residue was isolated using flash chromatography (hexanes:EA = 5:1) to give **10a**.

Methyl 3-(7-((tert-butyldimethylsilyl)oxy)-6-methoxy-1,3-dioxo-1,3-dihydro-2*H*-benzo[4,5]thieno[2,3-*c*]pyrrol-2-yl)propanoate (**10a**)

Yellow solid, 62% yield, MS: calcd for C_21_H_28_NO_6_SSi (M+H), 450.1; found, 450.3.

*Tert*-butyl 3-(7-((tert-butyldimethylsilyl)oxy)-6-methoxy-1,3-dioxo-1,3-dihydro-2*H*-benzo[4,5]thieno[2,3-*c*]pyrrol-2-yl)propanoate (**10b**)

Yellow solid, 81% yield, MS: calcd for C_24_H_34_NO_6_SSi (M+H), 492.2; found, 492.3.

**Synthesis of intermediate 11a~b**

TBAF (0.25 mL, 1 M in THF) was added into the solution of **10a** (75.0 mg, 0.17 mmol) in THF (2 mL). The solution was stirred at room temperature for 0.5 h. The solvent was removed under reduced pressure. The residue was isolated using flash chromatography (hexanes:EA = 2:1~1:1~DCM:EA = 8:1) to afford **11a**.

Methyl 3-(7-hydroxy-6-methoxy-1,3-dioxo-1,3-dihydro-2*H*-benzo[4,5]thieno[2,3-*c*]pyrrol-2-yl)propanoate (**11a**)

Yellow solid, 64% yield, MS: calcd for C_15_H_14_NO_6_S (M+H), 336.1; found, 336.3.

*Tert*-butyl 3-(7-hydroxy-6-methoxy-1,3-dioxo-1,3-dihydro-2*H*-benzo[4,5]thieno[2,3-*c*]pyrrol-2-yl)propanoate (**11b**)

Yellow solid, 63% yield, MS: calcd for C_18_H_20_NO_6_S (M+H), 378.1; found, 378.3.

**Synthesis of target compound ZSA-51D and 13b**

To a flask were added **11a** (20.7 mg, 0.06 mmol), K_2_CO_3_ (16.6 mg, 0.12 mmol), catalytic amount of NaI, CH_3_CN (1 mL) and 1,3-dibromopropane (18 µL, 0.18 mmol). The mixture was heated at 80°C for 3 h. The solvent was removed under high vacuum to give crude **12a**. MS: calcd for C_18_H_19_BrNO_6_S (M+H), 456.1; found, 456.3 (^79^Br), 458.3 (^81^Br).

To the residue above were added **11a** (14.0 mg, 0.04 mmol), K_2_CO_3_ (16.6 mg, 0.12 mmol), catalytic amount of NaI and CH_3_CN (1 mL). The resulted mixture was heated at 80 °C for 4 h. The solvent was removed. The residue was isolated on a silica gel chromatography column (hexanes:EA = 2:1~1:1) to give product which was triturated in the mixed solvent of hexanes (0.5 mL) and EA (0.5 mL). The slurry was filtered, washed with hexanes:EA (1:1), and dried to furnish ZSA-51D.

Dimethyl 3,3'-((propane-1,3-diylbis(oxy))bis(6-methoxy-1,3-dioxo-1,3-dihydro-2*H*-benzo[4,5]thieno[2,3-*c*]pyrrole-7,2-diyl))dipropionate (ZSA-51D)

Yellow solid, 9% overall yield, ^1^H NMR (600 MHz, CDCl_3_) δ 7.58 (s, 2H), 7.31 (s, 2H), 4.39 (t, *J* = 6.0 Hz, 4H), 3.98 (d, *J* = 5.7 Hz, 10H), 3.72 (s, 6H), 2.75 (t, *J* = 7.2 Hz, 4H), 2.52 (p, *J* = 6.0 Hz, 2H). ^13^C NMR (150 MHz, CDCl_3_) δ 171.4 (2C), 164.3 (2C), 163.5 (2C), 151.2 (2C), 149.6 (2C), 141.3 (2C), 138.7 (2C), 137.9 (2C), 124.8 (2C), 105.2 (2C), 104.5 (2C), 65.6 (2C), 56.2 (2C), 51.9 (2C), 34.1 (2C), 33.2 (2C), 29.0. MS: calcd for C_33_H_31_N_2_O_12_S_2_ (M+H), 711.1; found, 711.3. HPLC: (70% acetonitrile, UV detector: 254 nm), 95.9% purity.

Di*tert*-butyl 3,3'-((propane-1,3-diylbis(oxy))bis(6-methoxy-1,3-dioxo-1,3-dihydro-2*H*-benzo[4,5]thieno[2,3-*c*]pyrrole-7,2-diyl))dipropionate (**13b**)

Yellow solid, 24% overall yield, MS: calcd for C_39_H_43_N_2_O_12_S_2_ (M+H), 795.2; found, 795.3.

**Synthesis of target compound ZSA-52D**

To a flask were added **13b** (10.7 mg, 0.013 mmol), TFA (200 µL) and DCM (0.5 mL). The mixture was stirred at room temperature for 3 h. The solvent was removed under airflow. The residue was triturated in EA (200 µL), filtered and dried to give ZSA-52D.

3,3'-((propane-1,3-diylbis(oxy))bis(6-methoxy-1,3-dioxo-1,3-dihydro-2*H*-benzo[4,5]thieno[2,3-*c*]pyrrole-7,2-diyl))dipropionic acid (ZSA-52D)

Yellow solid, 83% yield, ^1^H NMR (600 MHz, DMSO-*d*_6_) δ 12.37 (s, 2H), 7.83 (s, 2H), 7.41 (s, 2H), 4.28 (t, *J* = 6.3 Hz, 4H), 3.88 (s, 6H), 3.75 (t, *J* = 7.3 Hz, 4H), 2.60 (t, *J* = 7.3 Hz, 4H), 2.32 (p, *J* = 6.3 Hz, 2H). ^13^C NMR (151 MHz, DMSO) δ 172.6 (2C), 164.3 (2C), 163.6 (2C), 151.0 (2C), 149.6 (2C), 141.1 (2C), 138.9 (2C), 137.7 (2C), 124.3 (2C), 106.6 (2C), 104.7 (2C), 65.8 (2C), 56.5 (2C), 34.4 (2C), 33.2 (2C), 29.1. MS: calcd for C_31_H_27_N_2_O_12_S_2_ (M+H), 683.1; found, 683.2. HPLC: (70% acetonitrile, UV detector: 254 nm), 95.3% purity.


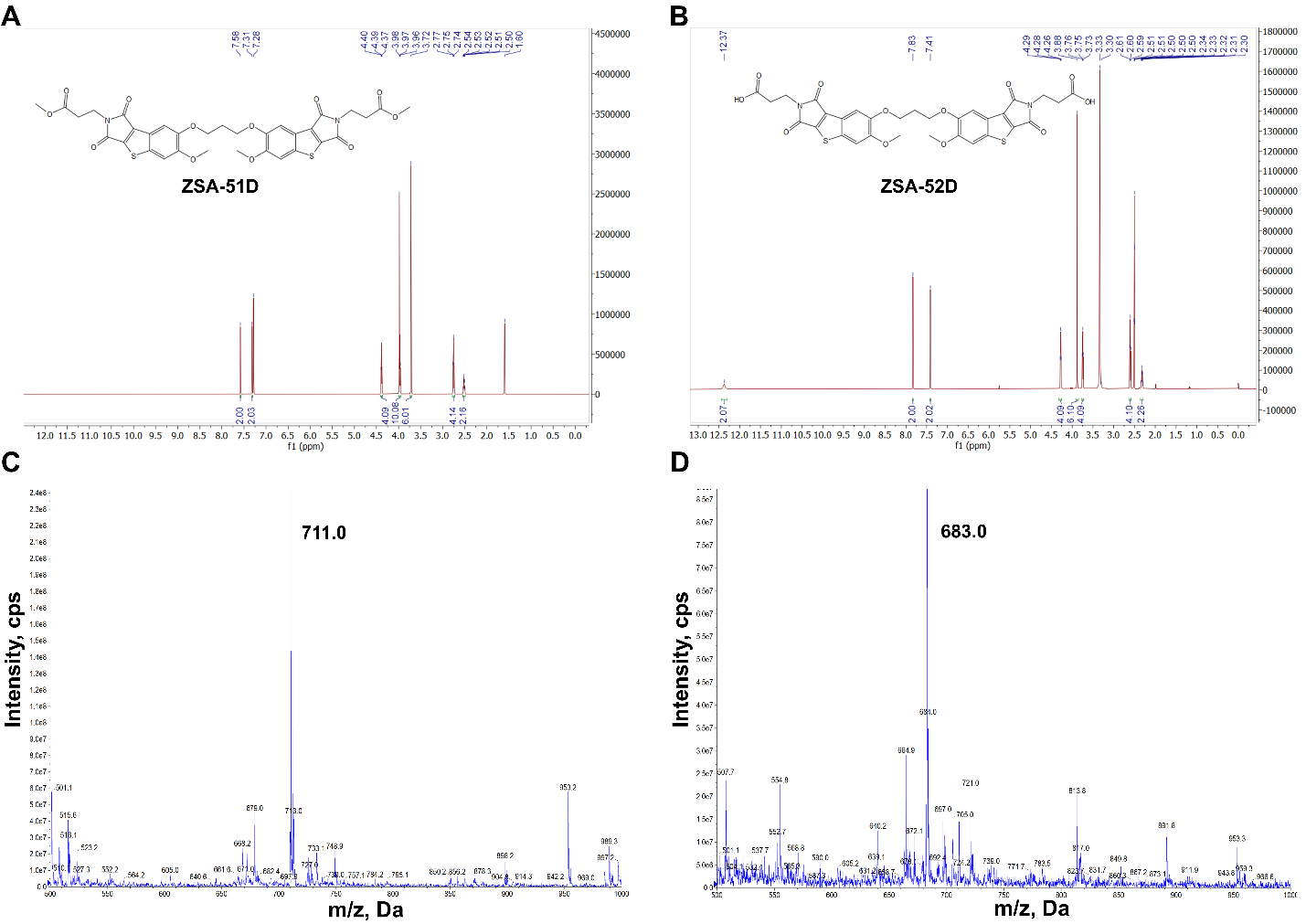


**Figure S1. Characterization of ZSA-51D and ZSA-52D.** (A, B) ^1^H NMR spectra of ZSA-51D (A) and ZSA-52D (B). (C, D) Mass spectra of ZSA-51D (C) and ZSA-51D (D).


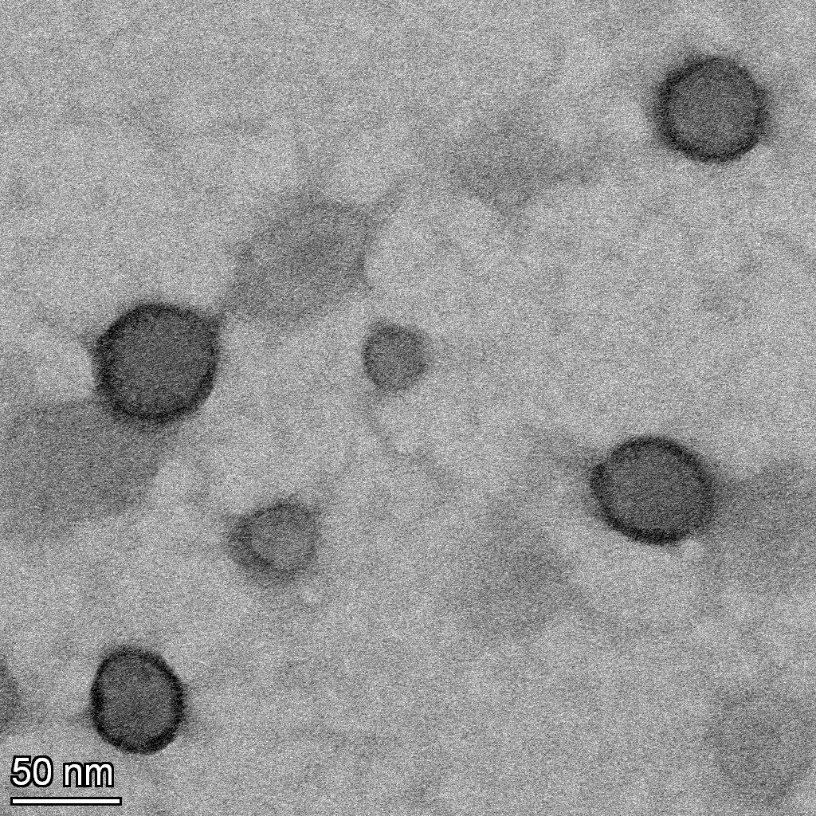


**Figure S2. Representative TEM image of Nano ZSA-51D.** The nanoparticles display uniform spherical morphology with a narrow size distribution. Scale bar, 50 nm.


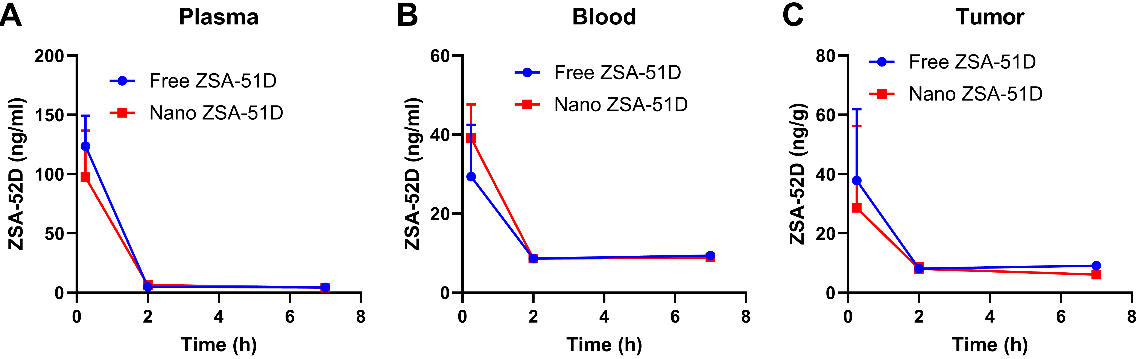


**Figure S3. Pharmacokinetic profiles of Nano ZSA-51D after systemically intravenous administration.** (A-C) Pharmacokinetic profiles of Free and Nano ZSA-51D (1 mg/kg, I.V.), showing the concentrations of ZSA-52D in plasma (A), blood (B), and tumor (C) at 15 minutes, 2 hours and 7 hours post-injection, quantified by LC-MS/MS (*n* = 3).


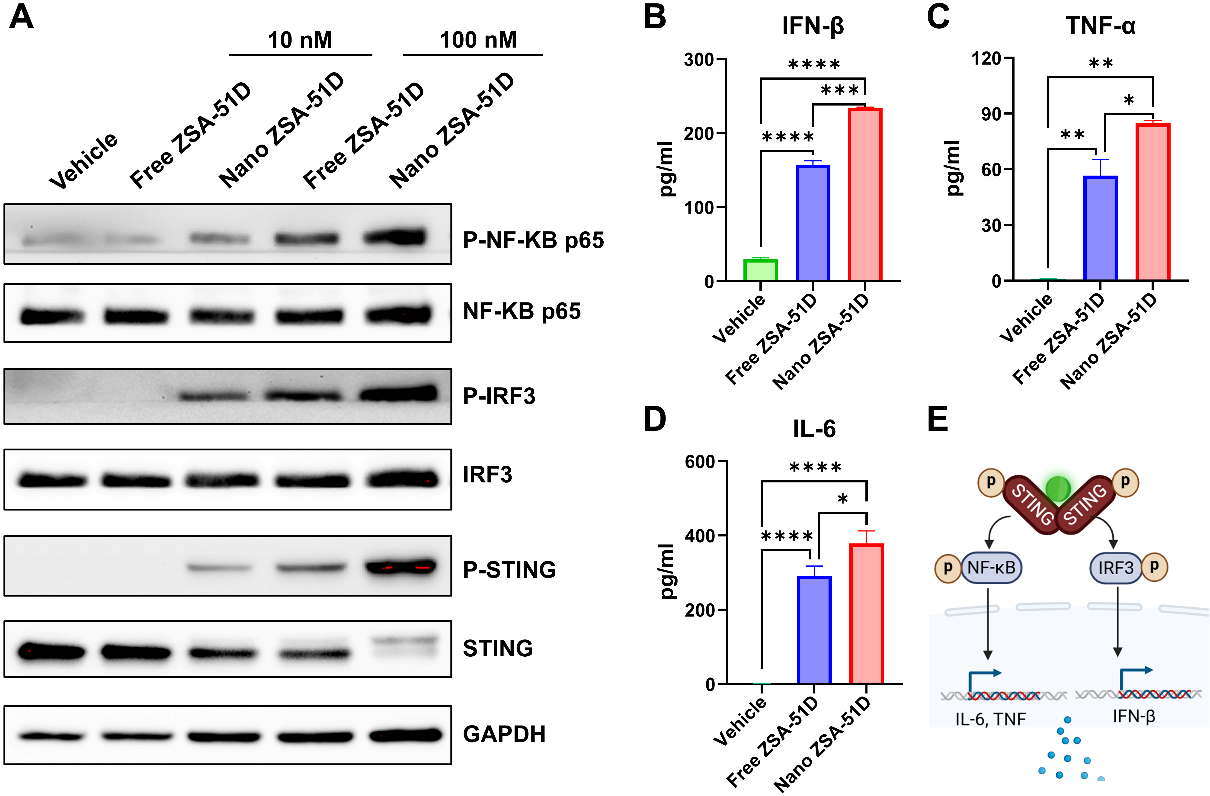


**Figure S4. Nano ZSA-51D activates STING signaling pathway in bone marrow cells.** (A) Western blot analysis of the activation of STING-IRF3 and STING-NF-κB pathway after free and Nano ZSA-51D (10 and 100 nm) in vitro treatments for 4 hours in bone marrow cells from C57BL/6J mice. (B-D) IFN-β (B), TNF-α (C) and IL-6 (D) secretion were measured using ELISA assay after free and Nano ZSA-51D (100 nm) treatments for 24 hours in bone marrow cells from C57BL/6J mice (*n* = 3, mean ± SD). One-way ANOVA with Tukey’s tests: **p* < 0.05, ***p* < 0.01, ****p* < 0.001, *****p* < 0.0001. (E) Schematic illustration showing Nano ZSA-51D activates the STING-IRF3 and STING-NF-κB pathways.


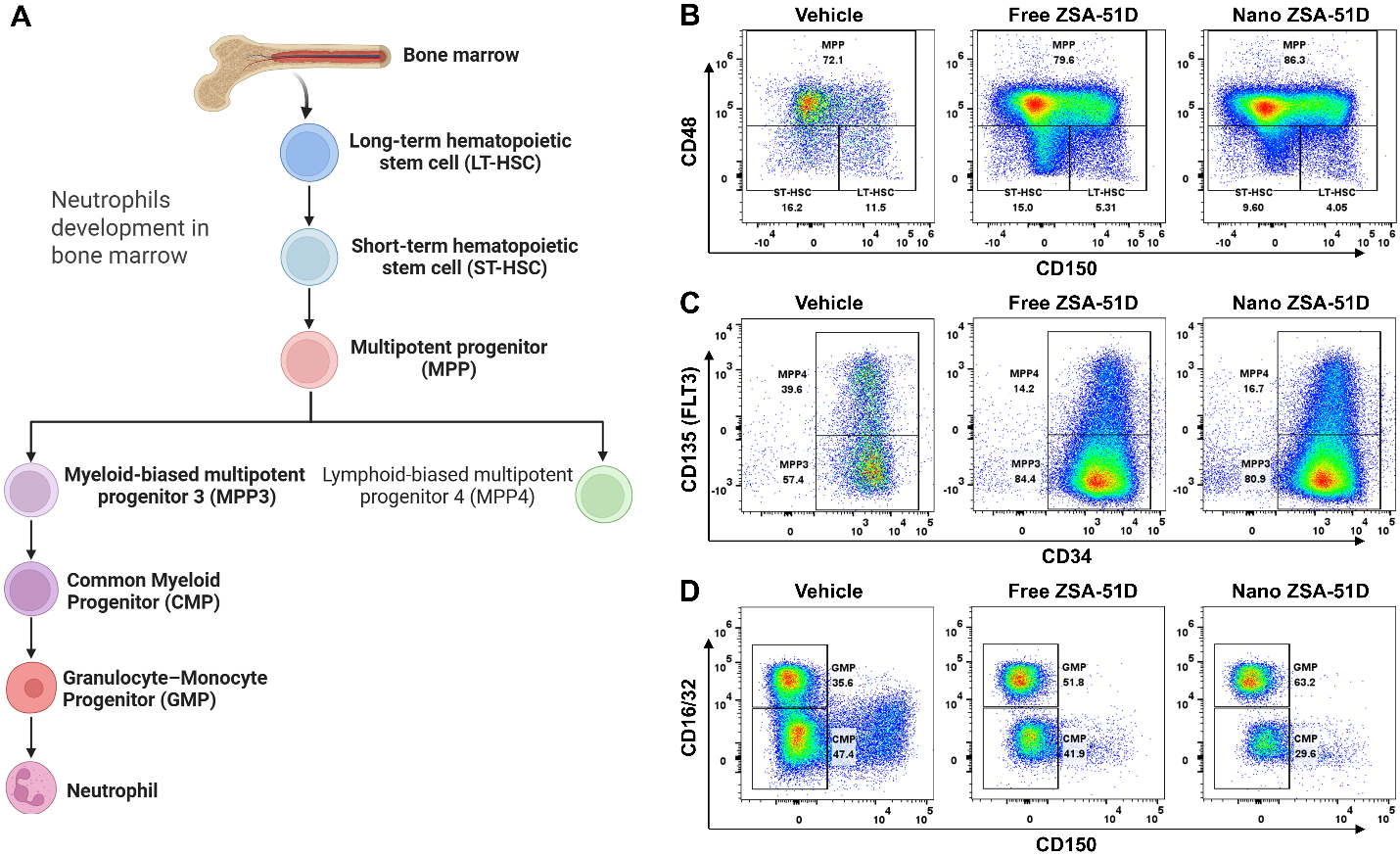


**Figure S5. Nano ZSA-51D expands HSPCs and skews differentiation toward GMP Cells.** (A) Schematic illustration showing the developmental trajectory of neutrophils differentiating from HSPCs in the bone marrow. (B-D) Representative flow cytometry plots of LT-HSC (CD150^+^CD48^-^), ST-HSC (CD150^-^CD48^-^) and MPP (CD150^-/+^CD48^+^) within LSK (Lin^-^Sca-1^+^c-Kit^+^) (B), MPP3 (CD34^+^CD135^-^) and MPP4 (CD34^+^CD135^+^) within MMP (Lin^-^Sca-1^+^c-Kit^+^CD150^-/+^CD48^+^) (C), GMP (CD150^-^CD16/32^+^) within LK (Lin^-^Sca-1^-^c-Kit^+^) (D) in bone marrow of MC-38 tumor bearing mice 24 hours post-treatment of free or Nano ZSA-51D (1 mg/kg, I.V.).


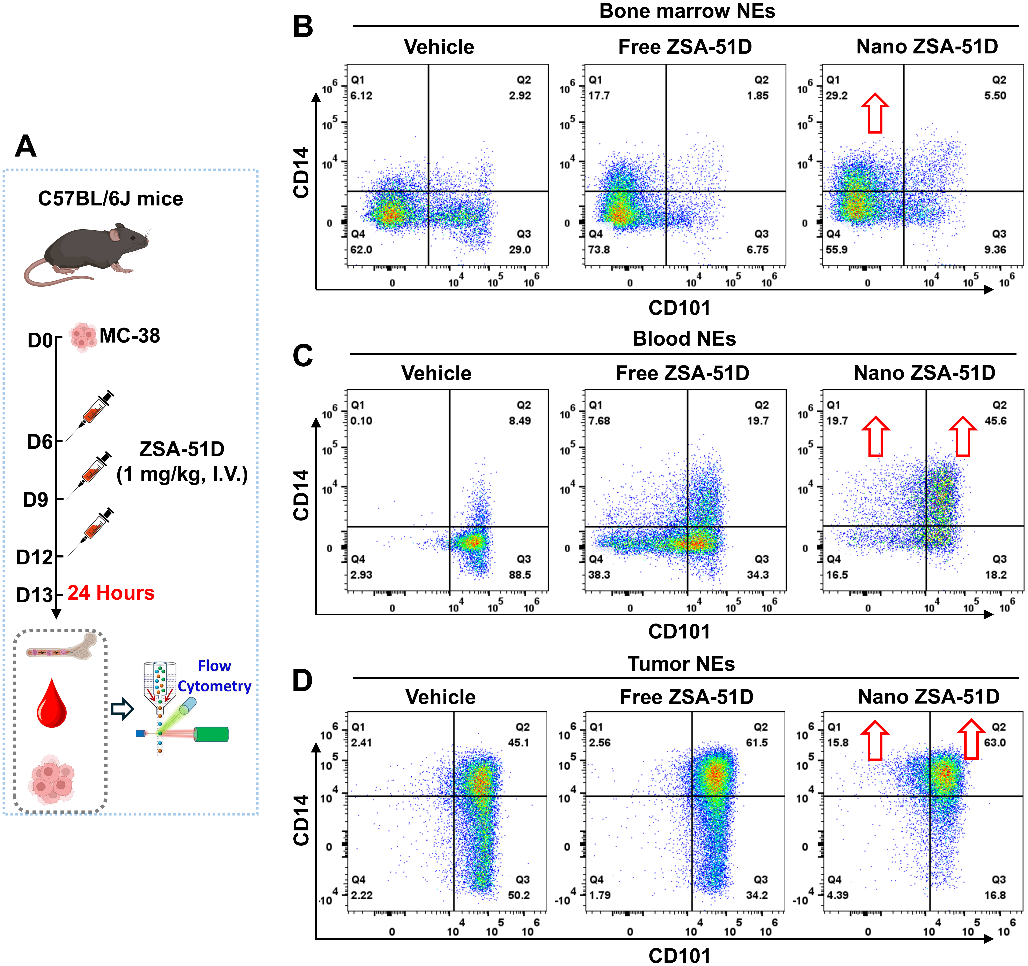


**Figure S6. Nano ZSA-51D reprograms immature (CD101^-^) and mature (CD101^+^) neutrophils into activated CD14⁺ subsets.** (A) Schematic of treatment schedule and flow cytometry analysis to assess neutrophil phenotypes in bone marrow blood and tumors of MC-38 tumor-bearing C57BL/6J mice at 24 hours post-treatment. (B-D) Representative flow cytometry plots of CD101^-^CD14^+^ and CD101^+^CD14^+^ neutrophil (NE) subtypes in bone marrow (B), blood (C) and tumors (D) of MC-38 tumor-bearing C57BL/6J mice 24 hours after systemic administration of free and Nano ZSA-51D (1 mg/kg).


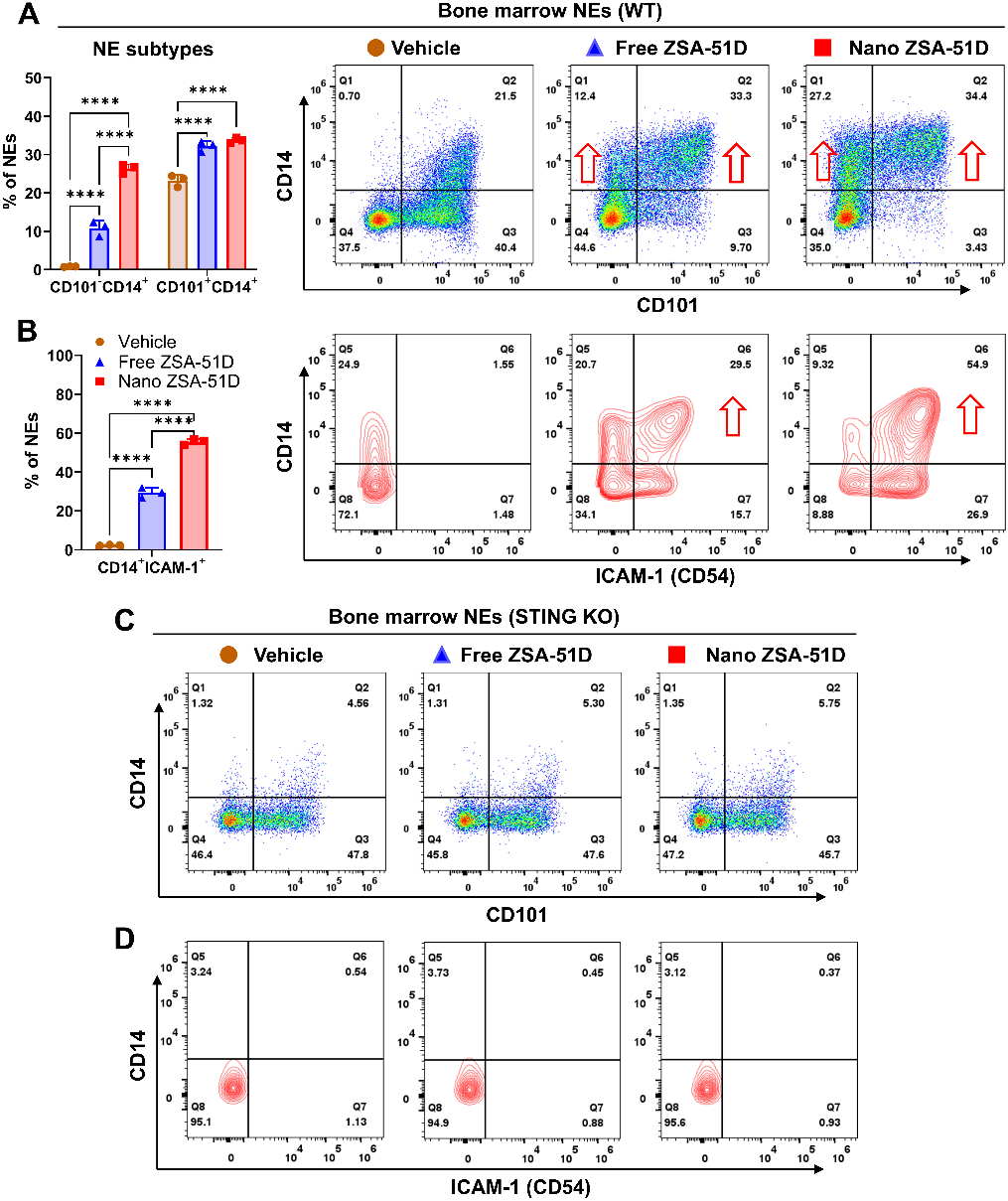


**Figure S7. Nano ZSA-51D-mediated reprogramming of CD14^+^ICAM-1^+^ neutrophils is STING-dependent.** (A-D) Quantification (left) and representative flow cytometry plots (right) of CD101^-^CD14^+^, CD101^+^CD14^+^ (A, C) and CD14^+^ICAM-1^+^ (B, D) neutrophils after overnight in vitro treatments with free and Nano ZSA-51D (100 nM) in bone marrow neutrophils from WT C57BL/6J mice (A, B) and STING KO mice (C57BL/6J-Sting1gt/J) (C, D) (*n* = 3, mean ± SD). One- (B) or two- (A) way ANOVA with Tukey’s tests: *****p* < 0.0001.


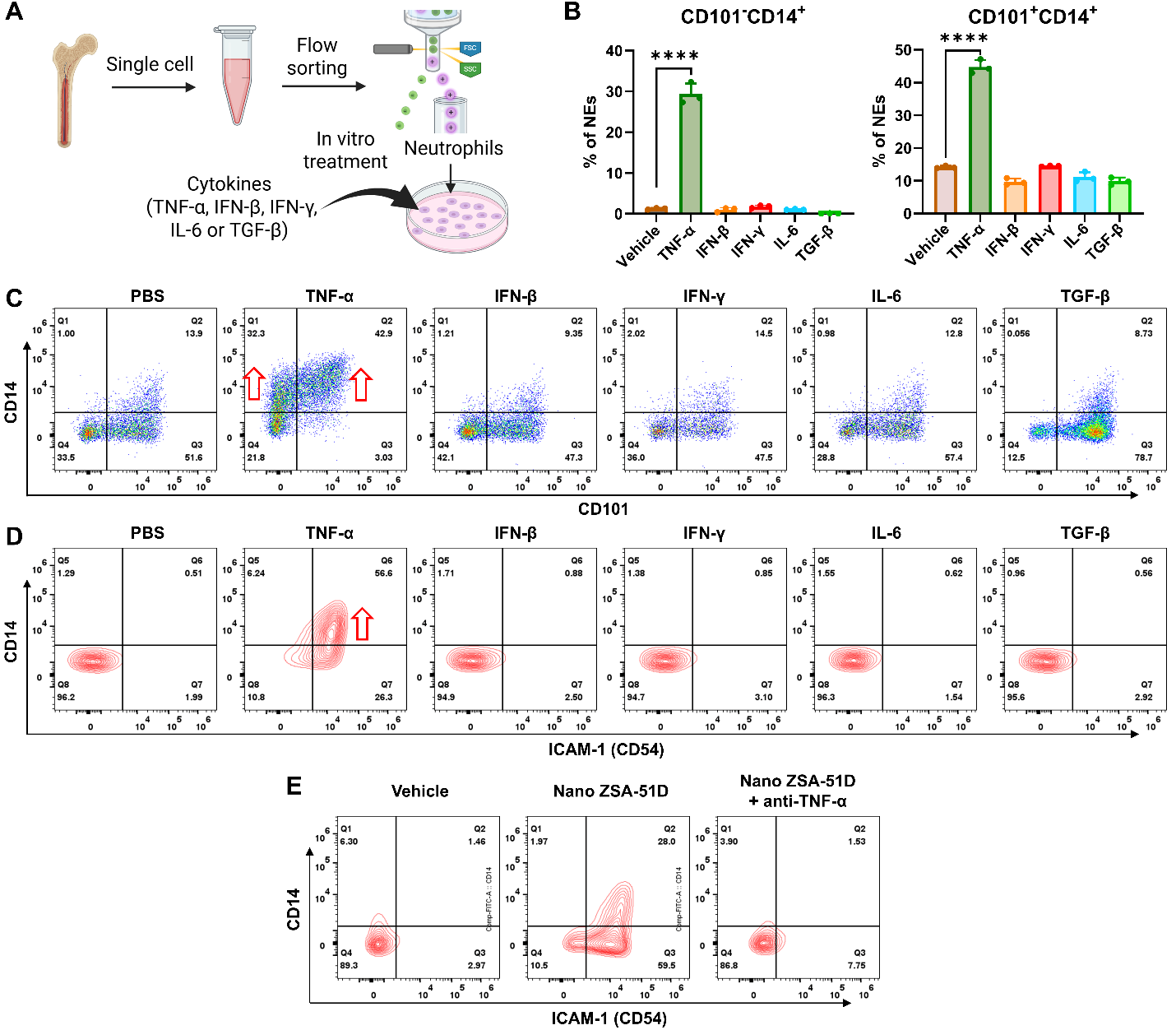


**Figure S8.** **Nano ZSA-51D** **reprograms bone marrow neutrophils into CD14⁺ICAM-1+ antitumor subsets via STING–NF-κB–TNF-α signaling.** (A) Experimental schematic showing the in vitro treatment of bone marrow neutrophils via cytokines (10 ng/mL). (B-D) Quantification (B) and representative flow cytometry plots (C, D) of CD101^-^CD14^+^, CD101^+^CD14^+^ (B, C) and CD14^+^ICAM-1^+^ (D) neutrophils following in vitro treatments of the STING pathway downstream cytokines (TNF-α, IFN-β, IFN-γ and IL-6) for overnight (*n* = 3, mean ± SD). One-way ANOVA with Tukey’s tests: *****p* < 0.0001. The immunosuppressive TGF-β as control. (E) Representative flow cytometry plots of CD14⁺ICAM-1⁺ neutrophils after overnight in vitro treatment with Nano ZSA-51D (100 nm) and TNF-α neutralization (10 μg/ml anti-TNF-α).


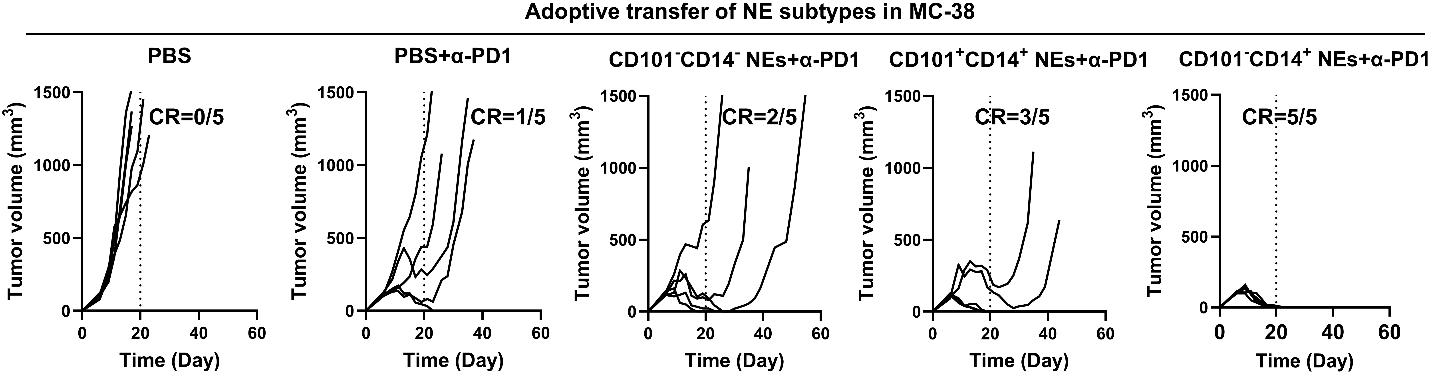


**Figure. S9 Antitumor efficacy after adoptive transfer of neutrophil subtypes with α-PD1 therapy.** Individual tumor growth curve of MC-38 tumor-bearing mice after adoptive transfer of CD101^-^CD14^-^, CD101^-^CD14^+^, or CD101^+^CD14^+^ neutrophils combined with α-PD1 treatment (n=5). CR, complete remission.


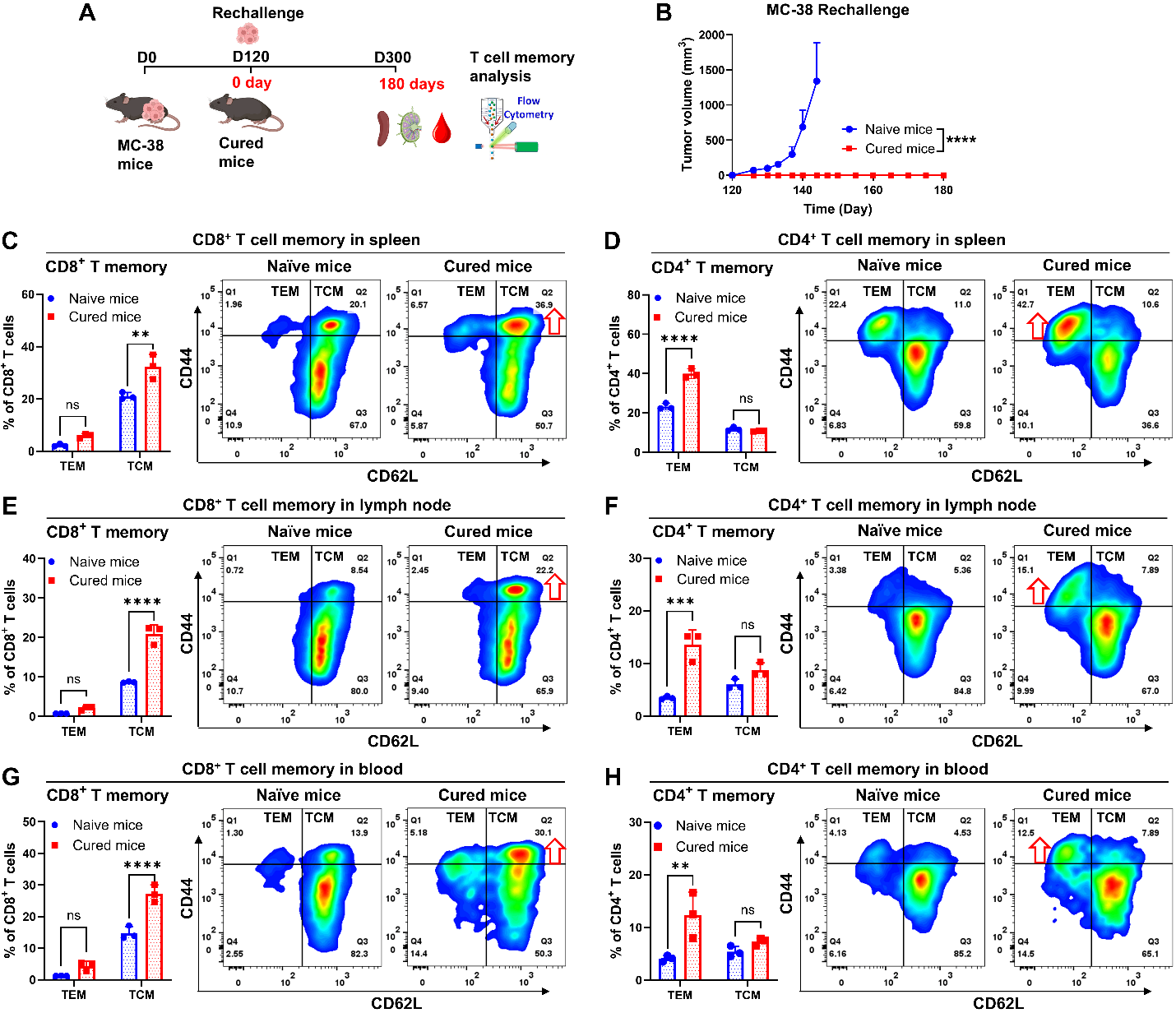


**Figure S10. Adoptive transfer of CD101^-^CD14⁺ neutrophils with α-PD1 therapy generates long-term CD8^+^ T cell memory.** (A) Schematic of MC-38 tumor cell rechallenge in mice cured with CD101-CD14+ neutrophils and α-PD1 combination therapy at 120 days post-treatment. Long-term T cell memory was evaluated by flow cytometry at 180 day post-rechallenge. (B) Tumor growth curves following MC-38 tumor cell rechallenge in naïve C57BL/6J mice and cured mice at 120 days post-treatment (*n*=5, mean ± SD). Two-way ANOVA with Sidak’s tests: *****p* < 0.0001. (C-H) Quantification (left) and representative flow cytometry plots (right) of CD8^+^ (C, E, G) and CD4^+^ (D, F, H) effector memory (TEM: CD62L^-^CD44^+^) and central memory (TCM: CD62L^+^CD44^+^) T cells in the spleen (C, D), lymph node (E, F) and blood (G, H) at 180-day post-rechallenge (n=5, mean ± SD). Two-way ANOVA with Sidak’s tests: ***p* < 0.01, ****p* < 0.001, *****p* < 0.0001, ns: not significant.


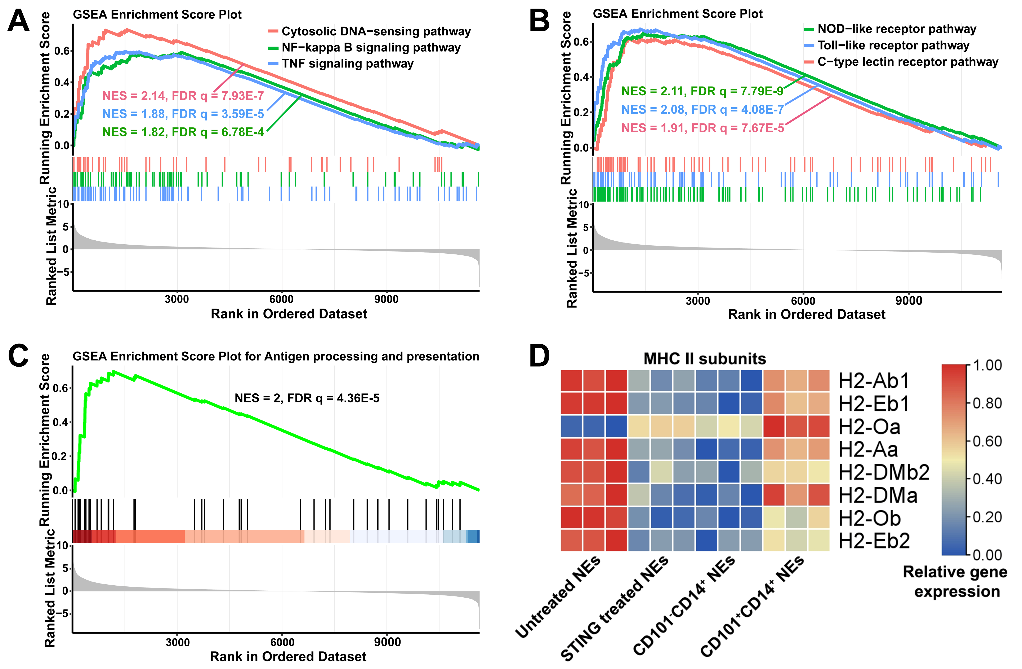


**Figure S11. Transcriptomic analysis of Nano ZSA-51D-reprogrammed bone marrow neutrophils.** (A-B) KEGG GESA enrichment score plots for cytosolic DNA-sensing, NF-kappa B, TNF signaling pathways (A) and NOD-like receptor, toll-like receptor, C-type lectin receptor pathways (B) in Nano ZSA-51D-treated bone marrow neutrophils. (C) KEGG GESA enrichment score plots for antigen processing and presentation pathway in Nano ZSA-51D-treated bone marrow neutrophils. (D) Heatmap of relative gene expression of MHC class II subunits in Nano ZSA-51D-treated neutrophils and their CD101^-^CD14^+^ and CD101^+^CD14^+^ subtypes.


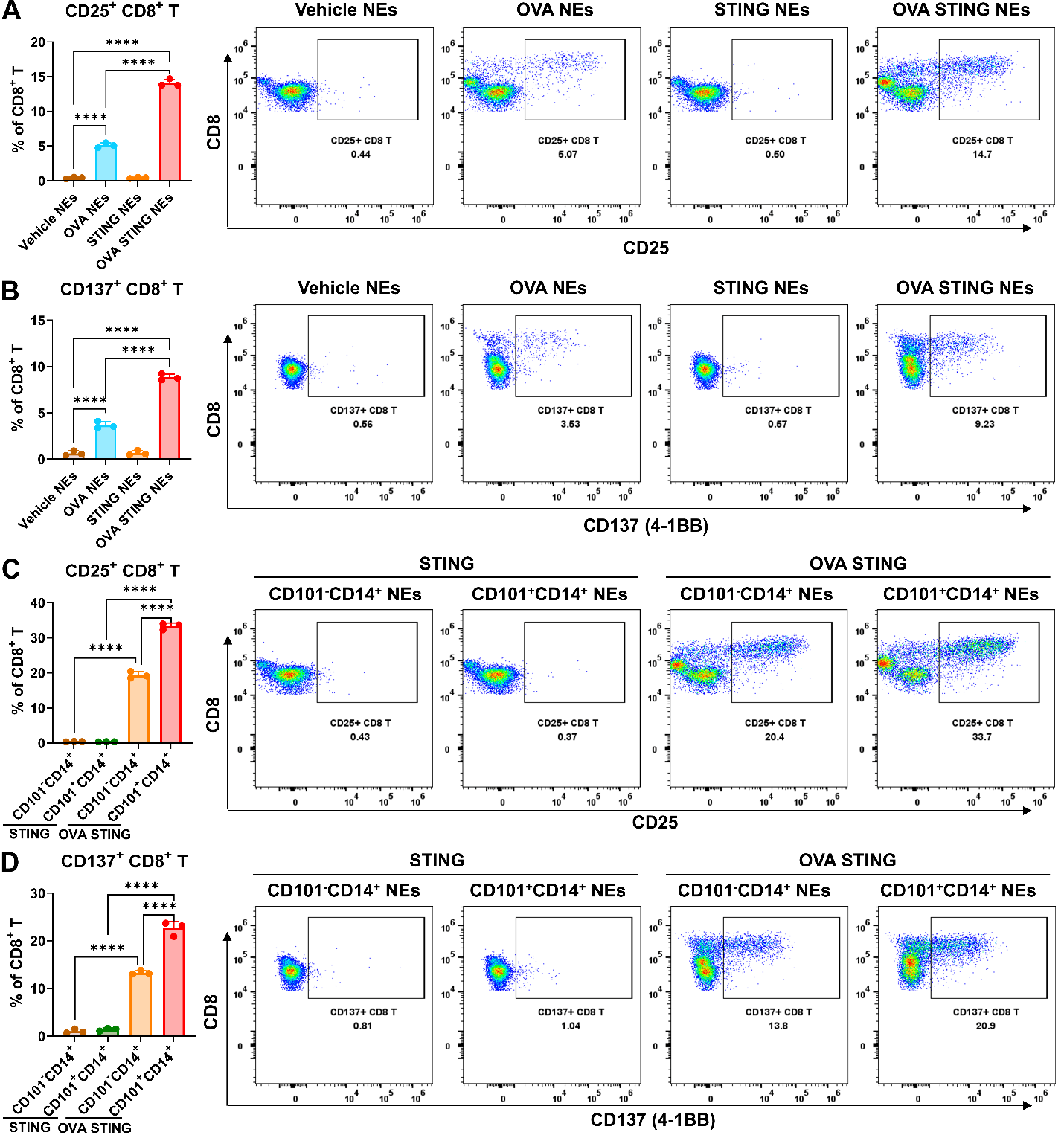


**Figure S12. Nano ZSA-51D-reprogrammed neutrophils prime antigen-specific CD8^+^ T cell responses.** (A, B) Quantification (left) and representative flow cytometry plots (right) of CD25^+^ (A) and CD137^+^ (B) OT-I CD8^+^ T cell activation after co-incubation with OVA or/and STING (Nano ZSA-51D) treated neutrophils (NEs) for 48 hours (*n* = 3, mean ± SD). *****p* < 0.0001. (C, D) Quantification (left) and representative flow cytometry plots (right) of CD25^+^ (C) and CD137^+^ (D) OT-I CD8^+^ T cell activation after co-incubation with OVA treated CD101^-^CD14^+^ and CD101^+^CD14^+^ neutrophils (NEs) for 48 hours (n=3, mean ± SD). One-way ANOVA with Tukey’s tests: *****p* < 0.0001.


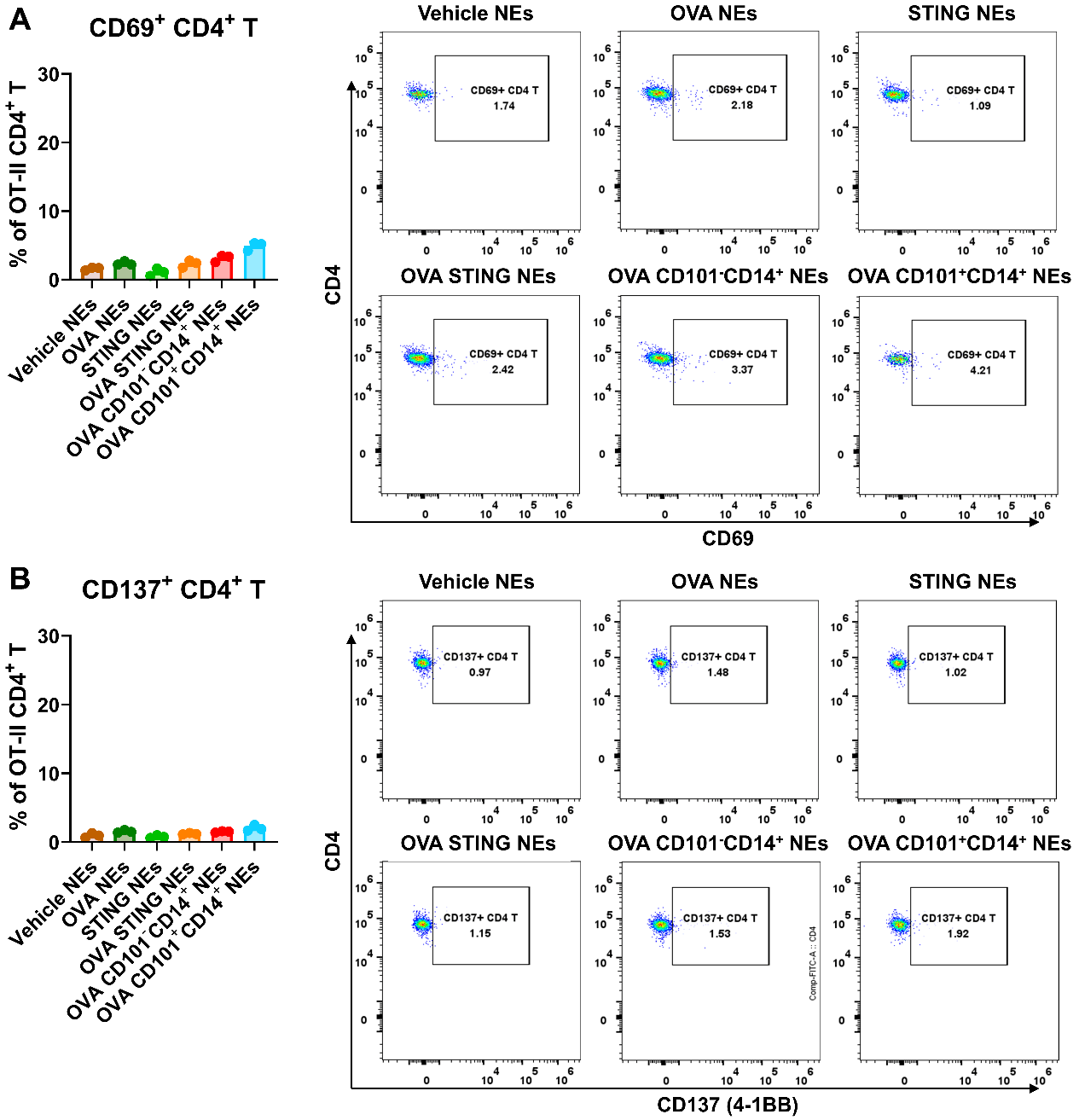


**Figure S13. Nano ZSA-51D-reprogrammed neutrophils displayed limited capacity to stimulate OT-II CD4⁺ T cells.** (A, B) Quantification (left) and representative flow cytometry plots (right) of CD69^+^ (A) and CD137^+^ (B) OT-II CD4^+^ T cell activation after co-incubation with OVA or/and STING (Nano ZSA-51D) treated neutrophils (NEs) for 48 hours (*n* = 3, mean ± SD).


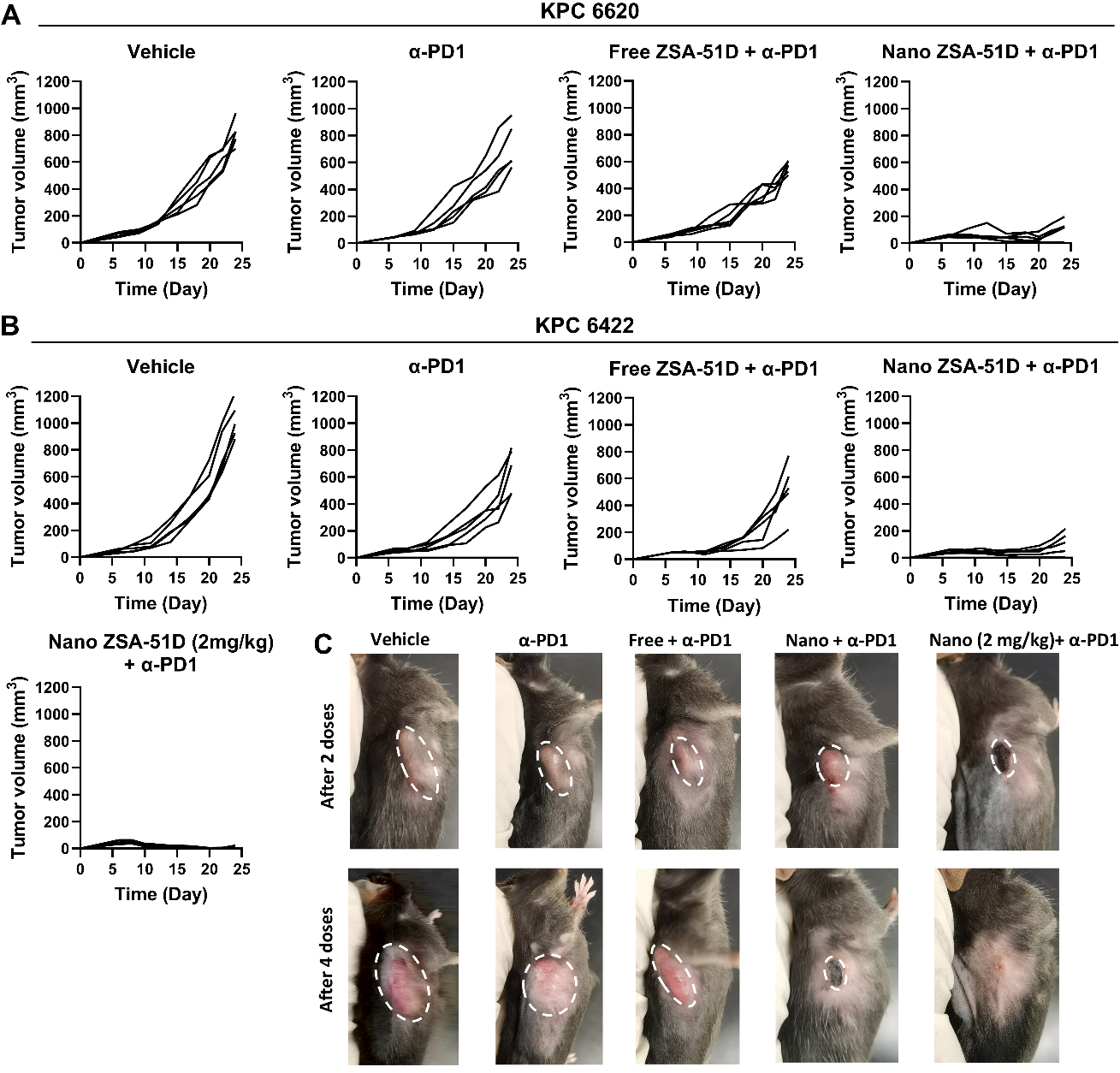


**Figure S14. Antitumor efficacy of Nano ZSA-51D combined with α-PD1 in KPC 6620 and 6422 pancreatic cancer models.** (A, B) Individual tumor growth trajectories of the KPC 6620 pancreatic cancer model following the indicated treatment regimens. (C) Representative tumor images of the KPC 6422 pancreatic cancer model at 2 and 4 doses post-treatment. The white dashed line indicates tumor margin.


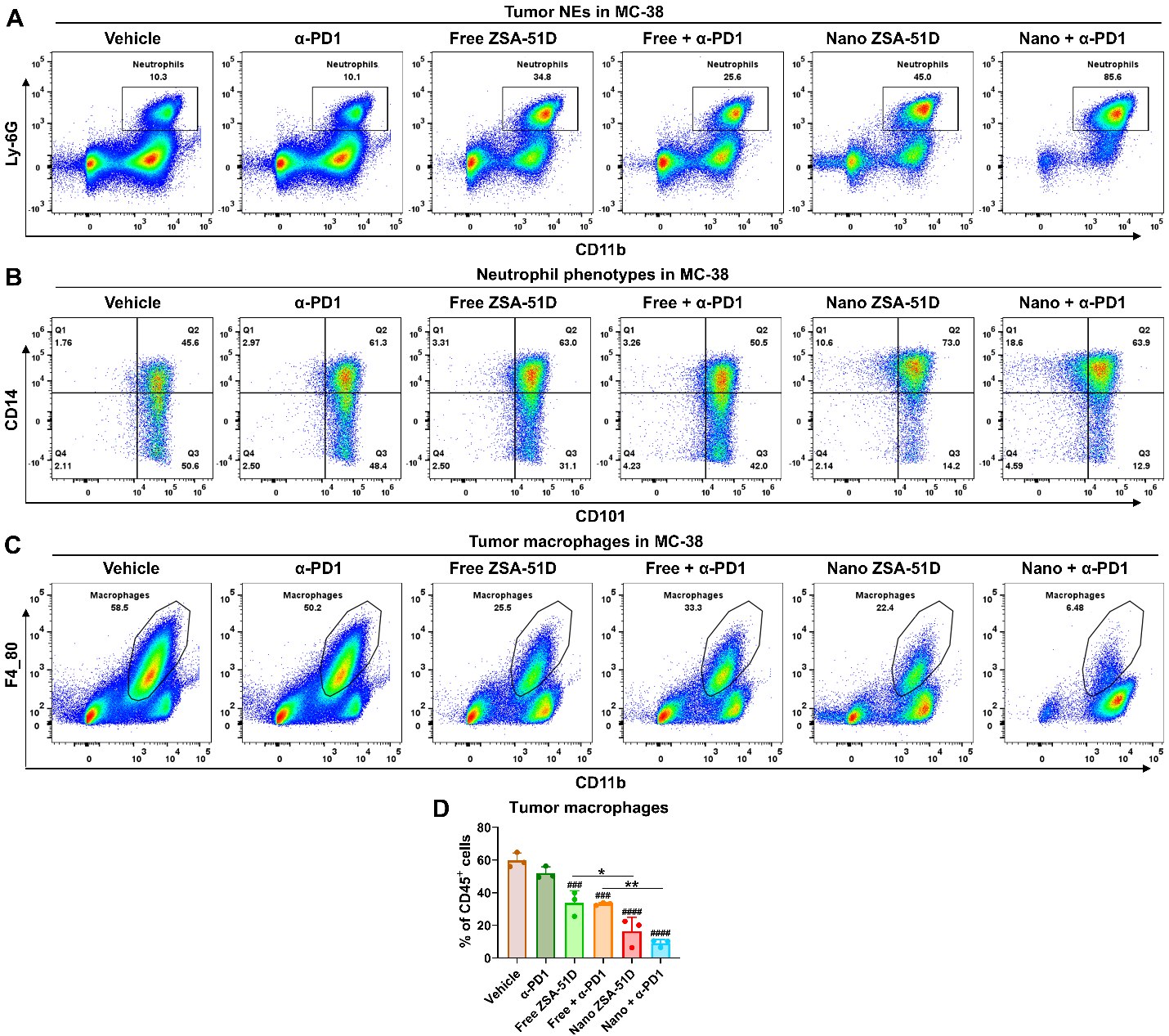


**Figure S15. Flow cytometric analysis of tumor-infiltrating neutrophils and macrophages at 1-day post-treatment in MC-38 tumor-bearing mice.** (A) Representative flow cytometry plots of tumor-infiltrating neutrophils (NEs) within CD45^+^ immune cells in MC-38 tumors at 1-day post-treatment. (B) Representative flow cytometry plots of CD101^-^CD14^+^ and CD101^+^CD14^+^ neutrophils in MC-38 tumors at 1-day post-treatment. (C-D) Representative flow cytometry plots (C) and quantification (D) of tumor-infiltrating macrophages within CD45^+^ immune cells in MC-38 tumors at 1-day post-treatment (*n* = 3, mean ± SD). One-way ANOVA with Tukey’s tests (*), or Dunnett’s tests for comparisons versus the vehicle group (#): **p* < 0.05, ***p* < 0.01; #*p* < 0.05, ###*p* < 0.001, ####*p* < 0.0001 vs. vehicle group.


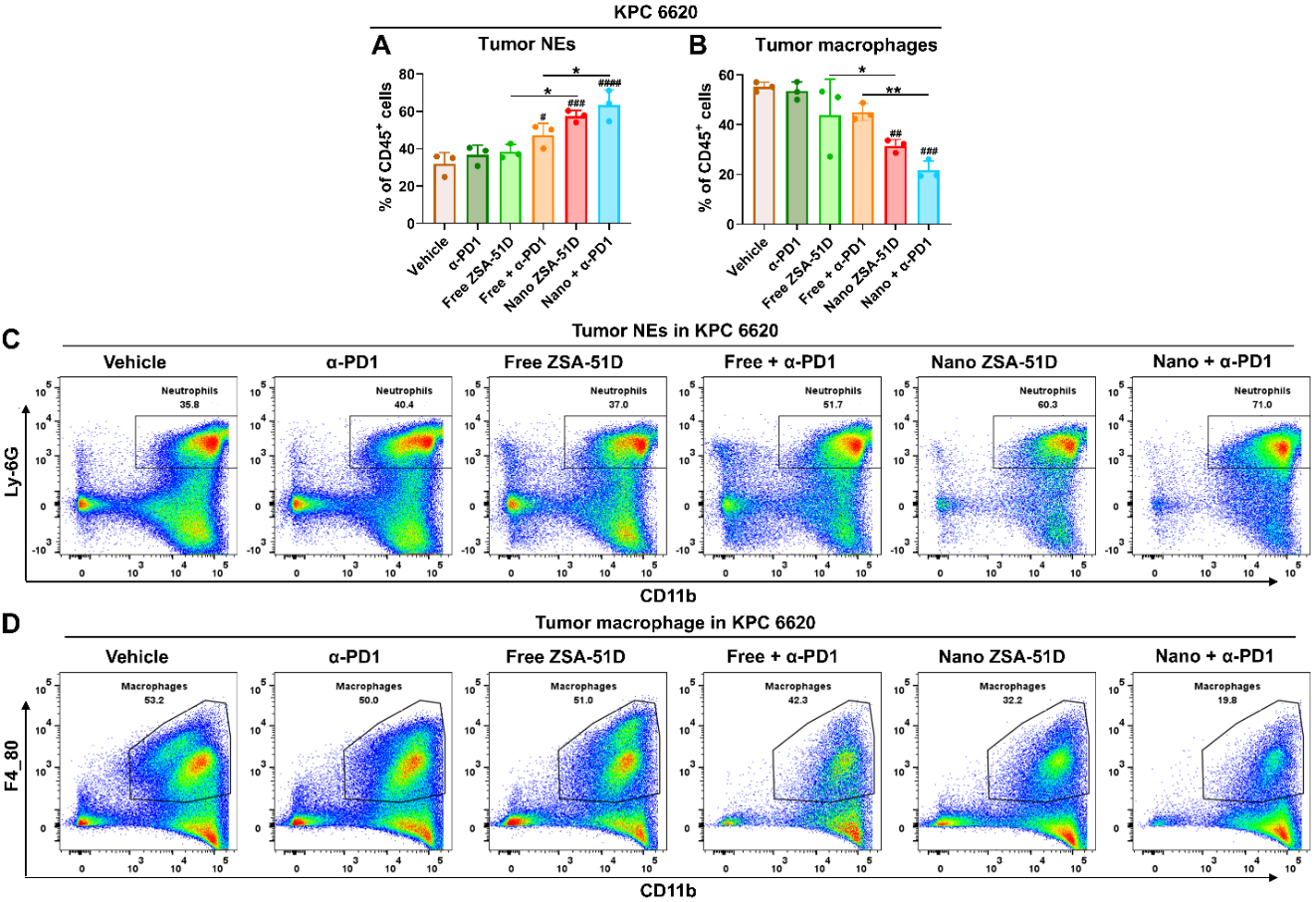


**Figure S16. Flow cytometric analysis of tumor-infiltrating neutrophils and macrophages at 1-day post-treatment in KPC 6620 tumor-bearing mice.** (A-D) Quantification (A, B) and representative flow cytometry plots (C, D) of tumor-infiltrating neutrophils (NEs) (A, C) and macrophages (B, D) within total CD45^+^ immune cells in KPC 6620 tumors at 1-day post-treatment (*n* = 3, mean ± SD). One-way ANOVA with Tukey’s tests (*), or Dunnett’s tests for comparisons versus the vehicle group (#): **p* < 0.05, ***p* < 0.01; #*p* < 0.05, ##*p* < 0.01, ###*p* < 0.001, ####*p* < 0.0001 vs. vehicle group. ns, not significant.


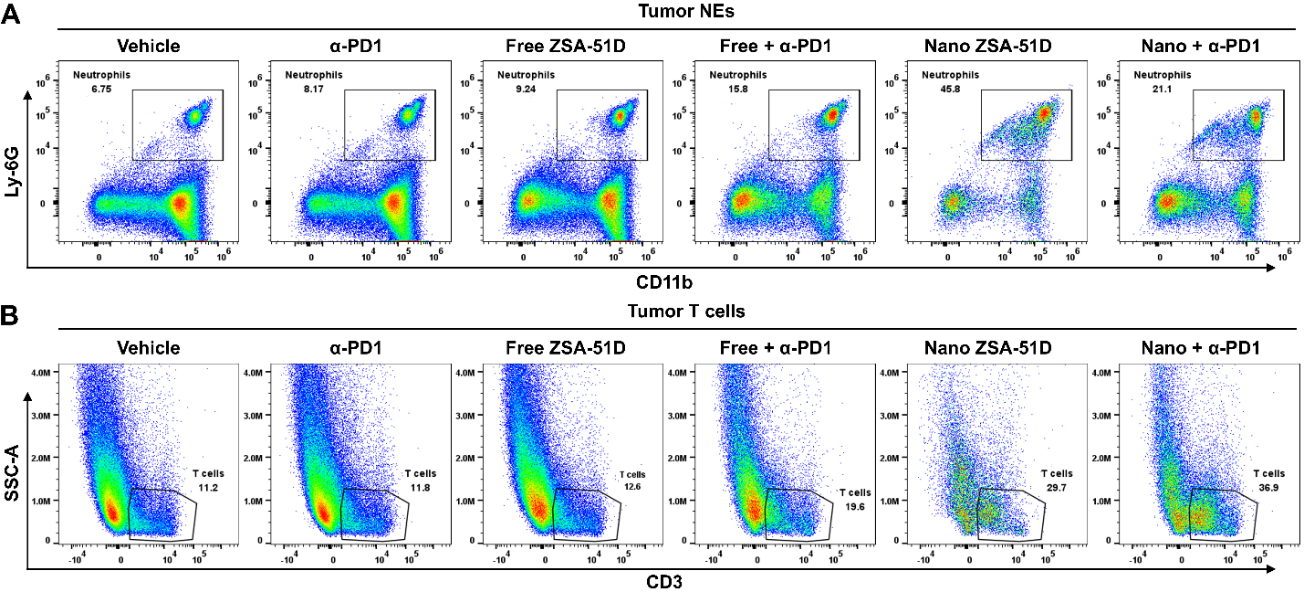


**Figure S17. Flow cytometric analysis of tumor-infiltrating neutrophils and T cells at 4-day post-treatment in MC-38 tumor-bearing mice.** (A, B) Representative flow cytometry plots showing tumor-infiltrating neutrophils (NEs) (A) and T cells (B) within total CD45^+^ immune cells in MC-38 tumors at 4-day post-treatment.


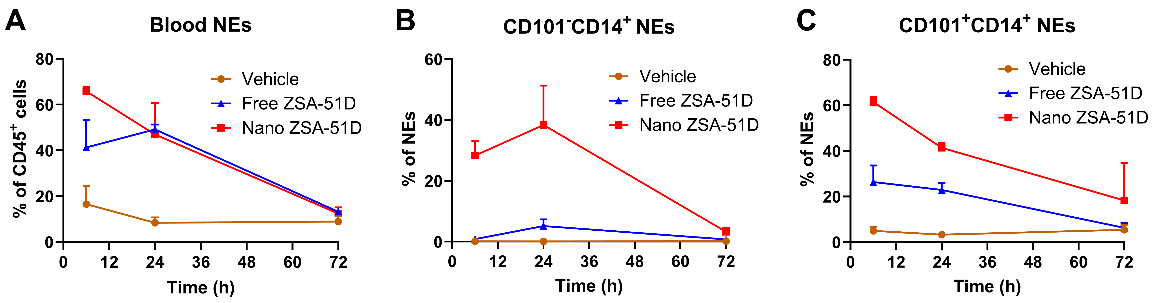


**Figure S18. Dynamic kinetics of circulating neutrophils following STING agonist treatment.** (A) Quantification of total blood neutrophils (NEs) among CD45^+^ immune cells at 6-, 24-, and 72- hour post-treatment of vehicle, free and Nano ZSA-51D (*n* = 3, mean ± SD). (B, C) Quantification of CD101^-^CD14^+^ (B) and CD101^+^CD14^+^ (C) NEs within total circulating neutrophils at 6-, 24-, and 72- hour post-treatment vehicle, free and Nano ZSA-51D (*n* = 3, mean ± SD).


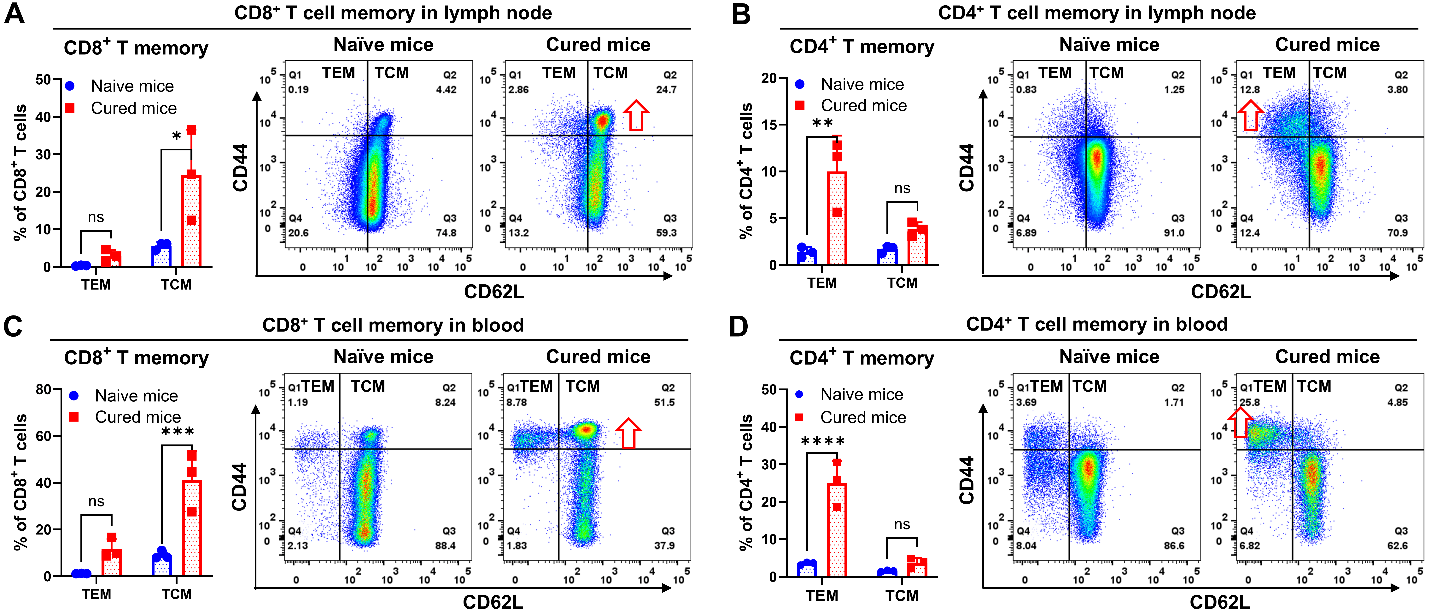


**Figure S19. Flow cytometric analysis of memory T cell responses in MC-38 cured mice by Nano ZSA-51D with α-PD1 therapy.** (A-D) Quantification (left) and representative flow cytometry plots (right) of CD8^+^ (A, C) and CD4^+^ (B, D) effector memory (TEM: CD62L-CD44+) and central memory (TCM: CD62L+CD44+) T cells in the lymph node (A, B) and blood (C, D) at 180-day post-rechallenge rechallenge (n=3, mean ± SD). Two-way ANOVA with Sidak’s tests: **p* < 0.05, ***p* < 0.01, ****p* < 0.001, ****p* < 0.001, ns: not significant.


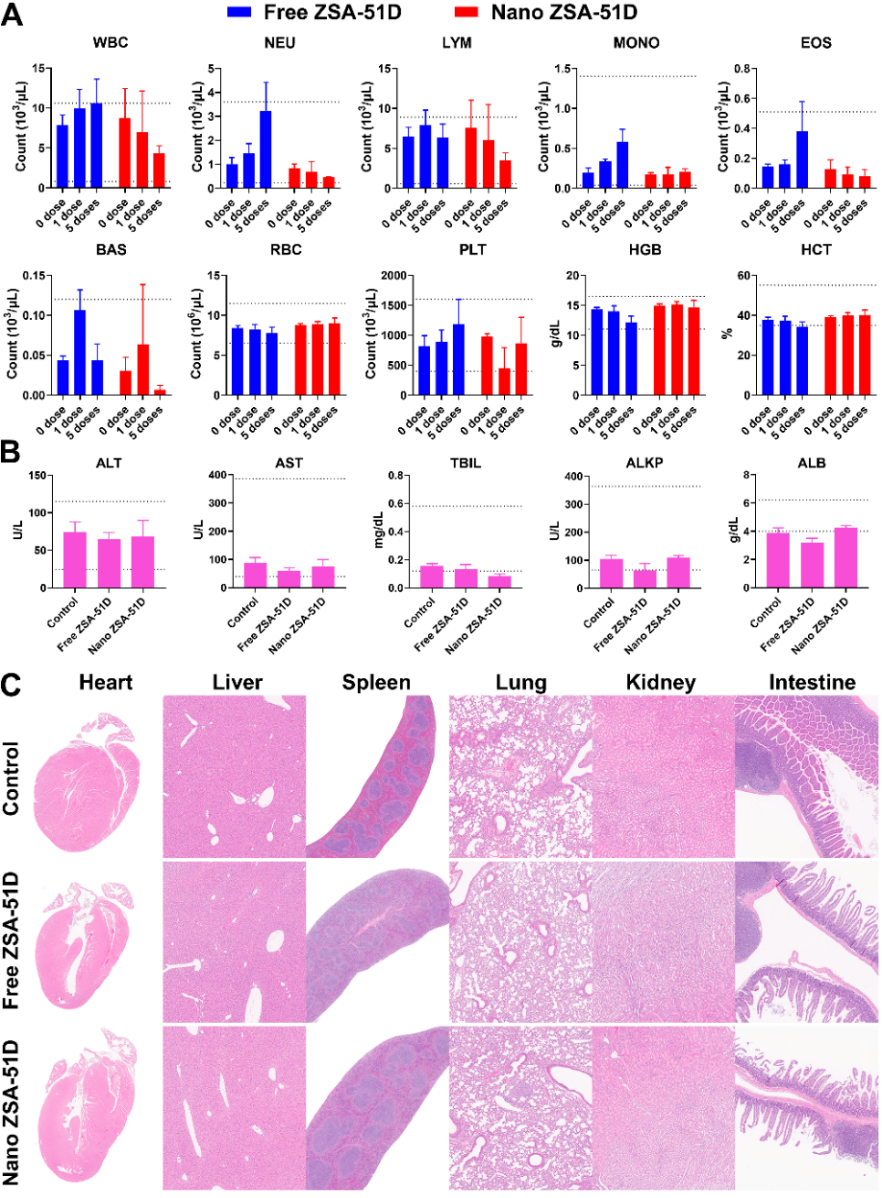


**Figure S20. Nano ZSA-51D showed minimal toxicity following single or multiple-dose treatment.** (A) Complete blood count, including white blood cells (WBC), neutrophils (NEU), lymphocytes (LYM), monocytes (MONO), eosinophils (EOS), basophils (BAS), red blood cells (RBC), platelets (PLT), hemoglobin (HGB) and hematocrit (HCT) in blood at 3 days after single or 5 doses of free or Nano ZSA-51D (1 mg/kg, I.V.) administered every 3 days. (B) Live function markers, including alanine aminotransferase (ALT), aspartate aminotransferase (AST), total bilirubin (TBIL), alkaline phosphatase (ALKP) and albumin (ALB) levels at 3 days after 5 doses of free or Nano ZSA-51D days (1 mg/kg, I.V.) administered every 3 days. (C) Histopathological analysis by hematoxylin and eosin (HE) staining of the heart, liver, spleen, lung, kidney, and intestine in C57BL/6J mice at 3 days after 5 doses of free or Nano ZSA-51D (1 mg/kg, I.V.) administered every 3 days.


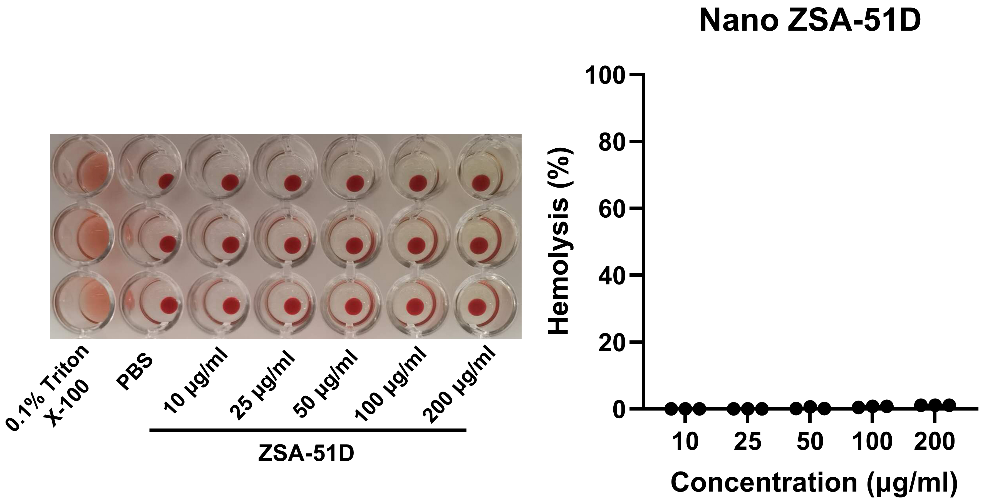


**Figure S21. Mouse hemolysis assay of Nano ZSA-51D**. PBS and 0.1% Triton X-100 were used as negative and positive controls, respectively.


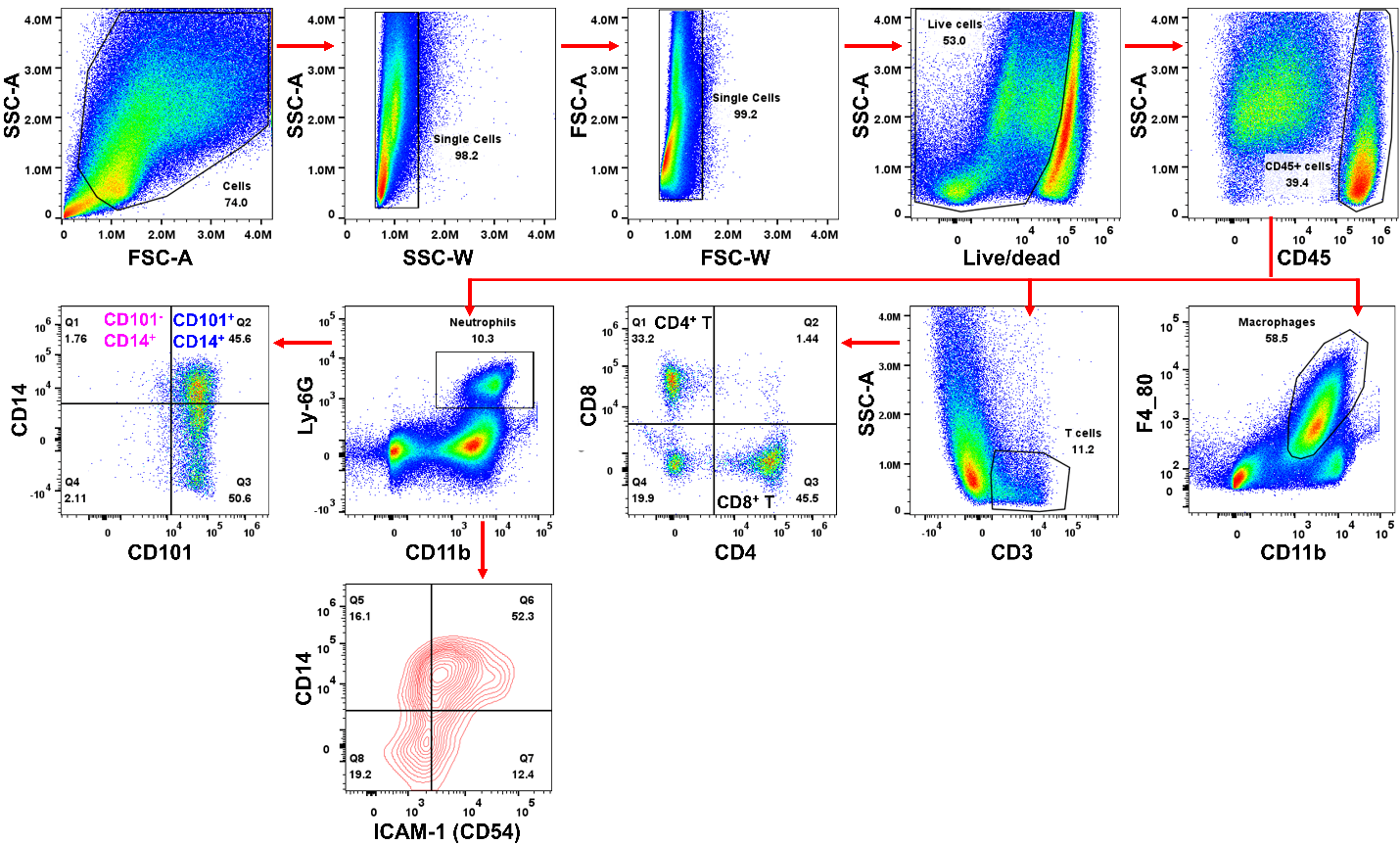


**Figure S22. Flow cytometric gating strategies of tumor infiltrating immune cells.** Gating strategies of tumor-infiltrating immune cells. Immune cell populations were defined as follows: neutrophils (CD45^+^CD11b^+^Ly6G^+^), macrophages (CD45^+^CD11b^+^F4_80^+^), total T cells (CD45^+^CD3^+^), CD4^+^ T cells (CD45^+^CD3^+^CD4^+^), and CD8^+^ T cells (CD45^+^CD3^+^CD8^+^). Neutrophil subsets were identified within the CD45^+^CD11b^+^Ly-6G^+^ gate based on CD101, CD14 and ICAM-1 expression.


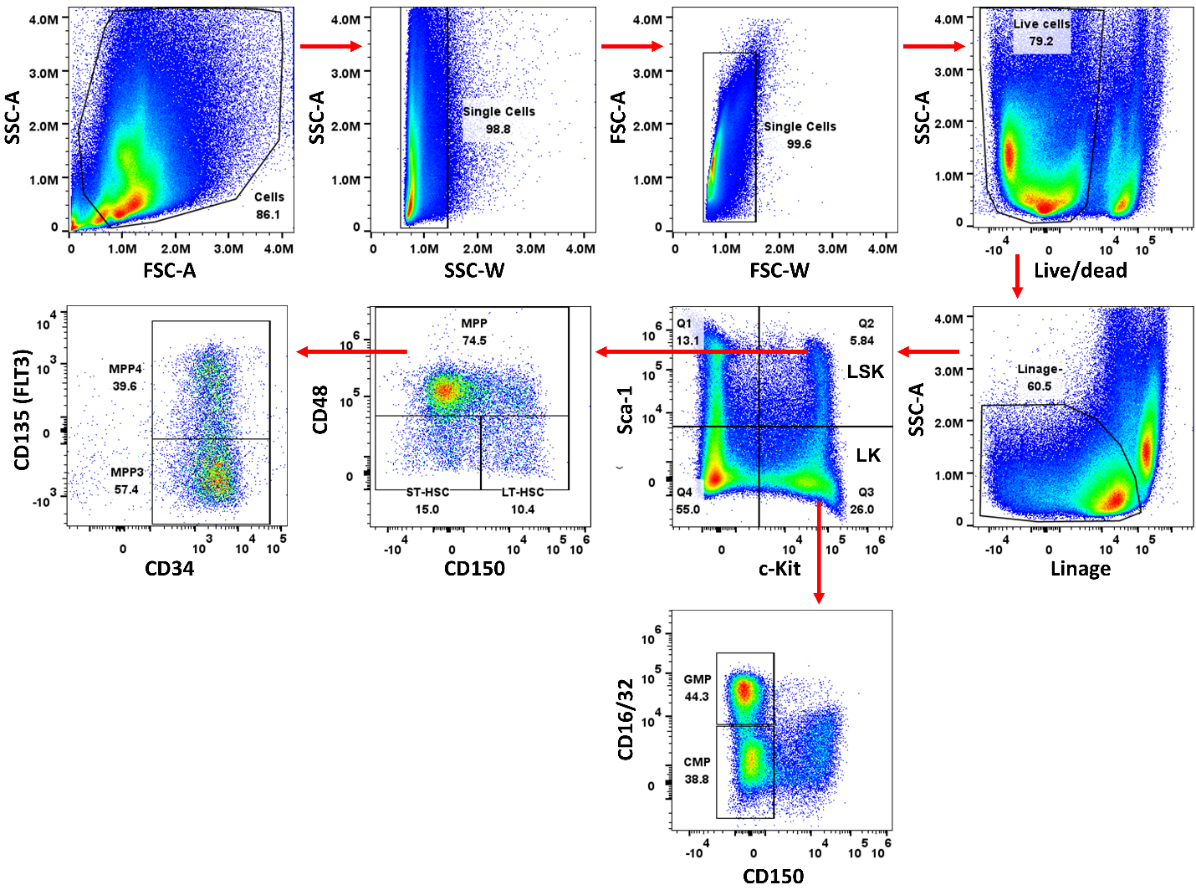


**Figure S23. Flow cytometric gating strategies of HSPCs in bone marrow.** HSPC components were defined as follows: LT-HSC (Lin^-^Sca-1^+^c-Kit^+^CD150^+^CD48^-^), ST-HSC (Lin^-^Sca-1^+^c-Kit^+^CD150^-^CD48^-^), MPP (Lin^-^Sca-1^+^c-Kit^+^CD150^-/+^CD48^+^), MPP3 (Lin^-^Sca-1^+^c-Kit^+^CD150^-/+^CD48^+^CD34^+^CD135^-^), MPP4 (Lin^-^Sca-1^+^c-Kit^+^CD150^-/+^CD48^+^CD34^+^CD135^+^), CMP (Lin^-^Sca-1^-^c-Kit^+^CD150^-^CD16/32^-^) and GMP (Lin^-^Sca-1^-^c-Kit^+^CD150^-^CD16/32^+^)


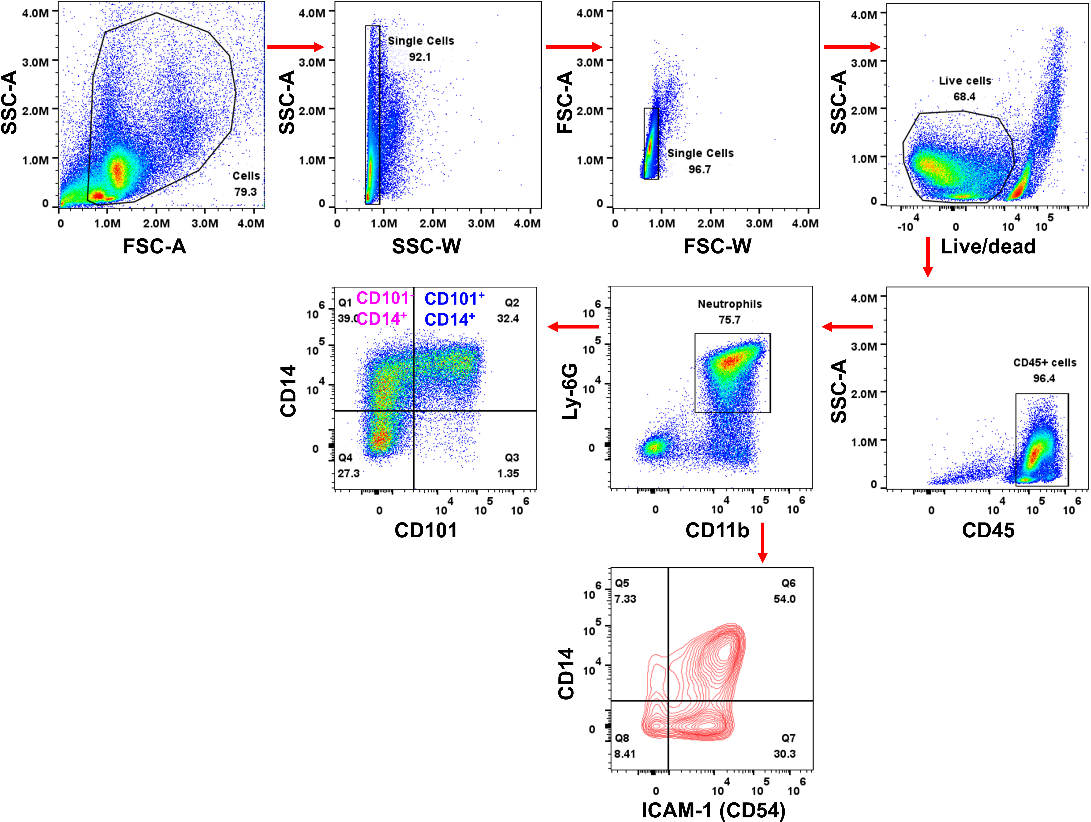


**Figure S24.** **Flow cytometric gating strategies of neutrophil subsets from STING agonist treated bone marrow.** Neutrophil subsets were identified within the CD45^+^CD11b^+^Ly-6G^+^ gate based on CD101, CD14 and ICAM-1 expression.


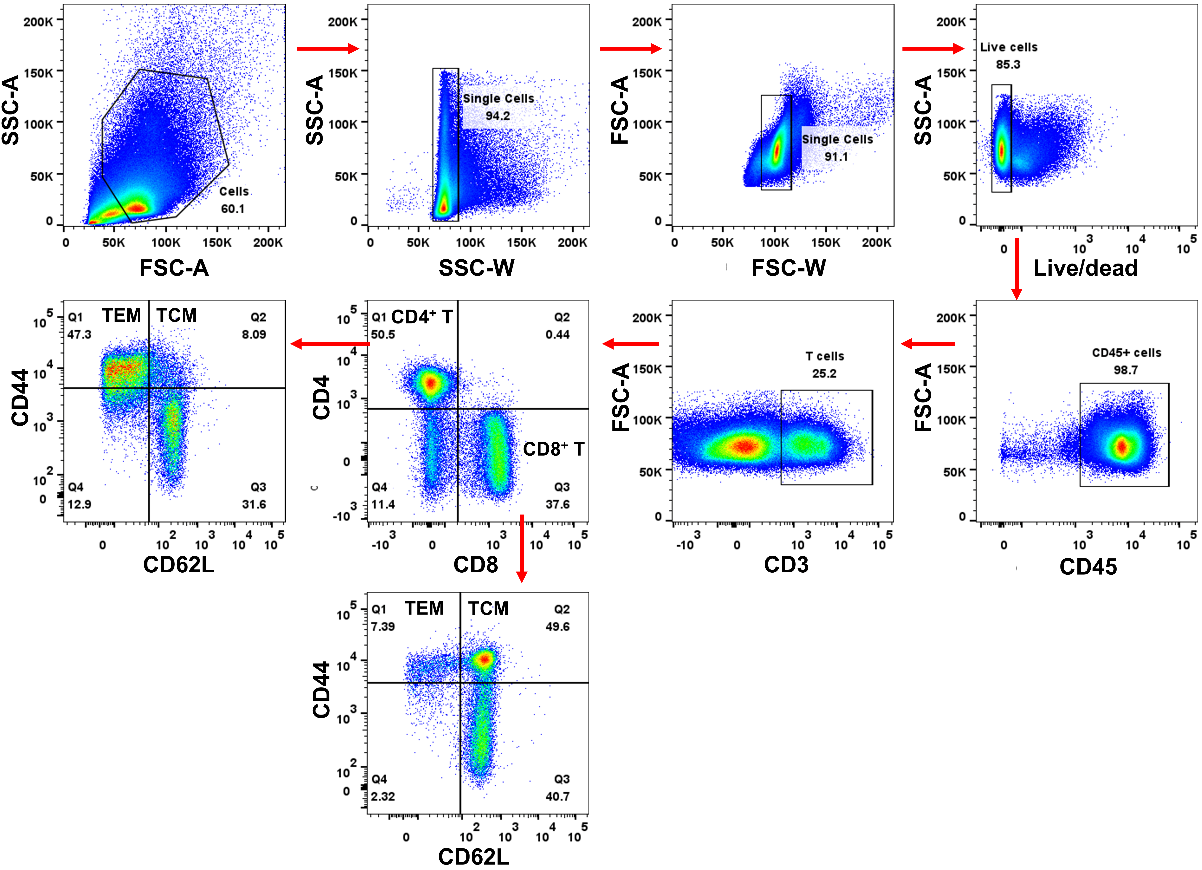


**Figure S25. Flow cytometric gating strategies of CD4^+^ and CD8^+^ T cell memory subsets.** The T cell memory populations were defined as follows: effector memory T cells (TEM, CD62L^-^CD44^+^) and central memory T cells (TCM, CD62L^+^CD44^+^).

**Table S1. Key reagent or resource table**

| **REAGENT or RESOURCE** | **SOURCE** | **IDENTIFIER** |
| --- | --- | --- |
| **Antibodies** | | |
| BV785 anti-mouse CD45 [30-F11] | Biolegend | Cat# 103149; RRID: AB_2564590 |
| AF594 anti-mouse CD11b [M1/70] | Biolegend | Cat# 101254; RRID: AB_2563231 |
| AF700 anti-mouse Ly-6G [1A8] | Biolegend | Cat# 127622; RRID: AB_10643269 |
| PE-Cy7 anti-mouse CD101 [Moushi101] | Thermo Scientific | Cat# 25-1011-82; RRID: AB_2573378 |
| FITC anti-mouse CD14 [Sa14-2] | Biolegend | Cat# 123308; RRID: AB_940580 |
| Pacific Blue anti-mouse CD54 [YN1/1.7.4] | Biolegend | Cat# 116116; RRID: AB_2121771 |
| PE anti-mouse H-2K^d^/H-2D^d^ (MHC I) [34-1-2S] | Biolegend | Cat# 114708; RRID: AB_313607 |
| BV421 anti-mouse I-A/I-E (MHC II) [M5/114.15.2] | Biolegend | Cat# 107607; RRID: AB_313322 |
| APC Anti-mouse H-2K^b^ bound to SIINFEKL [25-D1.16] | Biolegend | Cat# 141606; RRID: AB_11219595 |
| AF488 anti-mouse F4_80 [BM8] | BioLegend | Cat# 123120; RRID: AB_893479 |
| PE-Cy7 anti-mouse CD3 [17A2] | BioLegend | Cat# 100220; RRID: AB_1732057 |
| FITC anti-mouse CD4 [GK1.5] | BioLegend | Cat# 100406; RRID: AB_312691 |
| APC anti-mouse CD8a [53-6.7] | BioLegend | Cat# 100712; RRID: AB_312751 |
| BV605 anti-mouse CD19 [6D5] | BioLegend | Cat# 115540; RRID: AB_2563067 |
| PE anti-mouse CD69 [H1.2F3] | BioLegend | Cat# 104507; RRID: AB_313110 |
| BV785 anti-mouse CD25 [PC61] | BioLegend | Cat# 102051; RRID: AB_2564131 |
| PE-Cy7 anti-mouse CD137 (4-1BB) [17B5] | Thermo Scientific | Cat# 25-1371-80; RRID: AB_2573603 |
| APC-Fire750 anti-mouse CD4 [GK1.5] | BioLegend | Cat# 100460; RRID: AB_2566472 |
| AF594 anti-mouse CD8a [53-6.7] | BioLegend | Cat# 100758; RRID: AB_2563693 |
| APC anti-mouse CD44 [IM7] | BioLegend | Cat# 103012; RRID: AB_312957 |
| FITC anti-mouse CD62L [MEL-14] | BioLegend | Cat# 104405; RRID: AB_313114 |
| BV421 anti-mouse CD135 [A2F10] | BioLegend | Cat# 135313; RRID: AB_2107050 |
| APC-Cy7 anti-mouse CD34 [ | BioLegend | Cat# 128621; RRID: AB_2832465 |
| FITC anti-mouse CD16/32 [93] | BioLegend | Cat# 101305; RRID: AB_312804 |
| BV 785 anti-mouse CD150 [TC15-12F2.2] | BioLegend | Cat# 115937; RRID: AB_2565962 |
| PE anti-mouse CD48 [HM48-1] | BioLegend | Cat# 103405; RRID: AB_313020 |
| APC anti-mouse CD117 (c-kit) [2B8] | BioLegend | Cat# 105812; RRID: AB_313221 |
| PE-Cy7 anti-mouse Ly-6A/E (Sca-1) [D7] | BioLegend | Cat# 108114; RRID: AB_493596 |
| PE Anti-mouse Arginase-1 | BioLegend | Cat# 165803; RRID: AB_3068116 |
| Biotin anti-mouse Lineage Panel | BioLegend | Cat# 133307; RRID: AB_11124348 |
| TruStain FcX^TM^ PLUS anti-mouse CD16/32 [S17011E] | BioLegend | Cat# 156604; RRID: AB_2802064 |
| Phospho-STING (Ser365) (D8F4W) Rabbit mAb | Cell Signaling Technology | Cat# 72971S; RRID: AB_2799831 |
| STING (D2P2F) Rabbit mAb | Cell Signaling Technology | Cat# 13647S; RRID: AB_2799947 |
| Phospho-IRF 3 (Ser396) (4D4G) Rabbit mAb | Cell Signaling Technology | Cat# 4947S; |
| IRF 3 (D83B9) Rabbit mAb | Cell Signaling Technology | Cat# 4302S; RRID: AB_1904036 |
| Phospho-NF-κB p65 (Ser536) (93H1) Rabbit mAb | Cell Signaling Technology | Cat# 3033S; RRID: AB_331284 |
| NF-κB p65 (C22B4) Rabbit mAb | Cell Signaling Technology | Cat# 4764S; RRID: AB_823578 |
| HRP anti-Rabbit IgG Goat Secondary Antibody | Cell Signaling Technology | Cat# 7074S |
| Gapdh (D16H11) XP^®^ Rabbit mAb | Cell Signaling Technology | Cat# 5174S; RRID: AB_10622025 |
| Anti-mouse PD-1 [RMP1-14] | Bio X Cell | Cat# BE0146; RRID: AB_10949053 |
| Anti-mouse Ly-6G [1A8] | Bio X Cell | Cat# BP0075-1; RRID AB_2893871 |
| Anti-mouse CD8a [YTS 169.4] | Bio X Cell | Cat# BE0117; RRID AB_1569924 |
| **Chemical, peptide, and recombinant proteins** | | |
| QUANTI-Blue™ | InvivoGen | Cat# rep-qbs |
| Ovalbumin EndoFit™ | InvivoGen | Cat# vac-pova |
| Alexa Fluor® 594 Streptavidin | BioLegend | Cat# 405240 |
| Recombinant Mouse SCF | Biolegend | Cat# 579702 |
| Recombinant Mouse TNF-α | Biolegend | Cat# 575202 |
| Recombinant Mouse IFN-γ | Biolegend | Cat# 575302 |
| Recombinant Mouse IFN-β1 | Biolegend | Cat# 581302 |
| Recombinant Mouse IL-6 | Biolegend | Cat# 575702 |
| Recombinant Mouse TGF-β1 | R&D Systems | Cat# 7666-MB-005 |
| OT-I peptide (CSSSIINFEKL) | ChinaPeptides Co. | Cat# QU23001846 |
| Mouse serum albumin | Innovative Research | Cat# IMSALB1000MG |
| PEG-400 | Fisher Scientific | Cat# AC192230010 |
| Fixation Buffer | Biolegend | Cat# 420801 |
| InVivo Dilution Buffer | Bio X Cell | Cat# IP0070 |
| Collagenase/Hyaluronidase | STEMCELL | Cat# 07912 |
| DNase I | STEMCELL | Cat# 07900 |
| Red Blood Cell lysis buffer | Tonbo Biosciences | Cat# TNB-4300-L100 |
| Ghost Dye™ Violet 510 | Tonbo Biosciences | Cat# 13-0870-T100 |
| StemSpan^TM^ SFEM II | STEMCELL | Cat# 09605 |
| Halt Phosphatase Inhibitor Cocktail | Fisher Scientific | Cat# PI78420 |
| Halt Protease Inhibitor Cocktail | Fisher Scientific | Cat# PI78430 |
| Pierce RIPA Buffer | Fisher Scientific | Cat# PI89900 |
| **Critical commercial assays** | | |
| HTRF Human STING Binding Kit | Revvity | Cat# 64BDSTGPEG |
| Mouse TNF-α Quantikine ELISA Kit | R&D Systems | Cat# MTA00B-1 |
| Mouse IFN-β Quantikine ELISA Kit | R&D Systems | Cat# MIFNB0 |
| Mouse IL-6 DuoSet ELISA | R&D Systems | Cat# DY40605 |
| Mouse IFN-gamma ELISpot Kit | R&D Systems | Cat# EL485 |
| EasySepTM mouse CD8+ T cell isolation kit | STEMCELL | Cat# 19853 |
| Mojosort Streptavidin Nanobeads | BioLegend | Cat# 480016 |
| CellTrace™ Violet Cell Proliferation Kit | Thermo Scientific | Cat# C34571 |
| RNeasy Plus Mini Kit | QIAGEN | Cat# 74134 |
| Illumina NovaSeq X 10B platform | Illumina | Cat# 20085594 |
| **Experimental models: Cell lines** |  |  |
| THP-1-Blue™ ISG cells | InvivoGen | Cat# thp-isg |
| MC-38 cells | Kerafast | Cat# ENH204-FP; RRID: CVCL_B288 |
| KPC 6620 | Kerafast | Cat# EUP016-FP |
| KPC 6422 | Kerafast | Cat# EUP004-FP; RRID: CVCL_YM23 |
| **Experimental models: Organisms/strains** | | |
| C57BL/6J mice | Charles River Lab | Cat# 027; RRID: IMSR_CRL:027 |
| STING KO mice (C57BL/6J-Sting1gt/J) | Jackson Lab | Cat# 017537; RRID: IMSR_JAX:017537 |
| OT-I transgenic mice (Tg(TcraTcrb)1100Mjb/J) | Jackson Lab | Cat# 003831; RRID: IMSR_JAX:017537 |
| **Software and algorithms** | | |
| Graphpad Prism (v10.1.2) | GraphPad Prism software | <https://www.graphpad.com/>; RRID: SCR_002798 |
| FlowJo (v10.10.0) | BD Bioscience | <https://www.flowjo.com/solutions/flowjo>; RRID: SCR_008520 |
| RStudio (v4.4.1) | Posit, PBC | <https://posit.co/download/rstudio-desktop/>; RRID: SCR_000432 |
